# Supplementary material for: Genomic and panproteomic analysis of the development of infant immune responses to antigenically-diverse pneumococci
Source: Nat Commun. 2024 Jan 8;15:355. doi: 10.1038/s41467-023-44584-2 (PMC10774285; doi:10.1038/s41467-023-44584-2)
Supplement: Supplementary file 1 — Supplementary Information [file 41467_2023_44584_MOESM1_ESM.pdf]

Genomic and panproteomic analysis of the development of infant immune responses to antigenically-diverse pneumococci

Supplementary Materials

Supplementary Figures 1 – 68

Supplementary Tables 1 – 7

Supplementary Figures

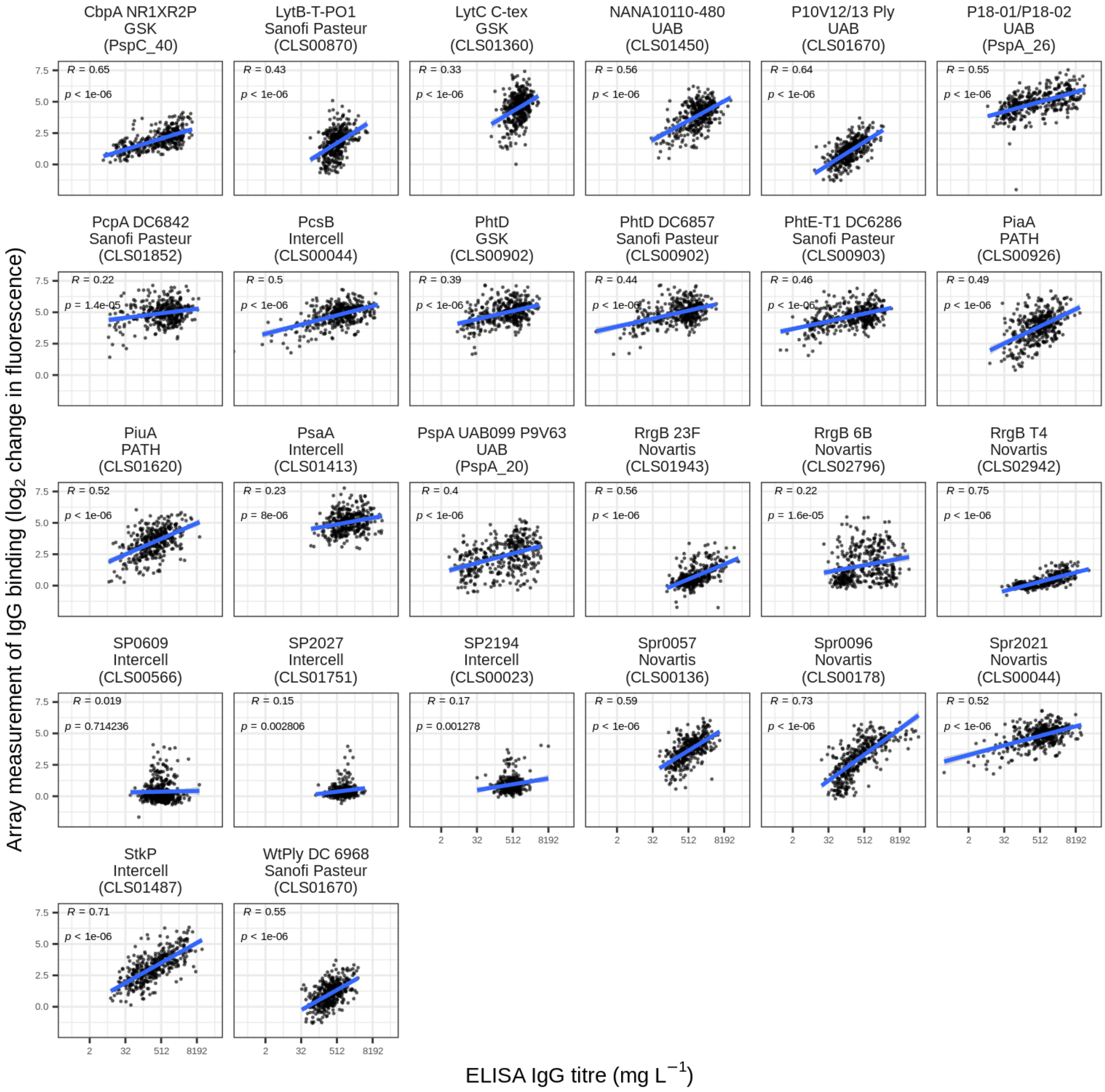

Supplementary Figure 1 Comparison of IgG binding estimates from the array and ELISA assays. Turner *et al* analysed the IgG responses to multiple candidate protein antigens supplied by vaccine developers using ELISA assays. The most similar protein on the array to each candidate antigen was identified, and the IgG responses compared in the set of serum samples that were used in both studies. Each plot is labelled with three pieces of information: the candidate antigen, the supplier, and the matched protein on the array. Each point represents a pair of measurements from the two technologies in a single individual at a specific timepoint. Linear models were fitted to the data following a base two logarithmic transformation of the ELISA data, to ensure it was scaled comparably to the array measurements. The significant positive correlation across most antigens validates the array measurements as being accurate representations of individuals' immune responses. Notably, the three exceptions (SP0609, SP2027 and SP2194) all originated from the same supplier. These deviations may be the consequence of proprietary modifications to these proteins, which were not strongly immunogenic in their native forms on the array.

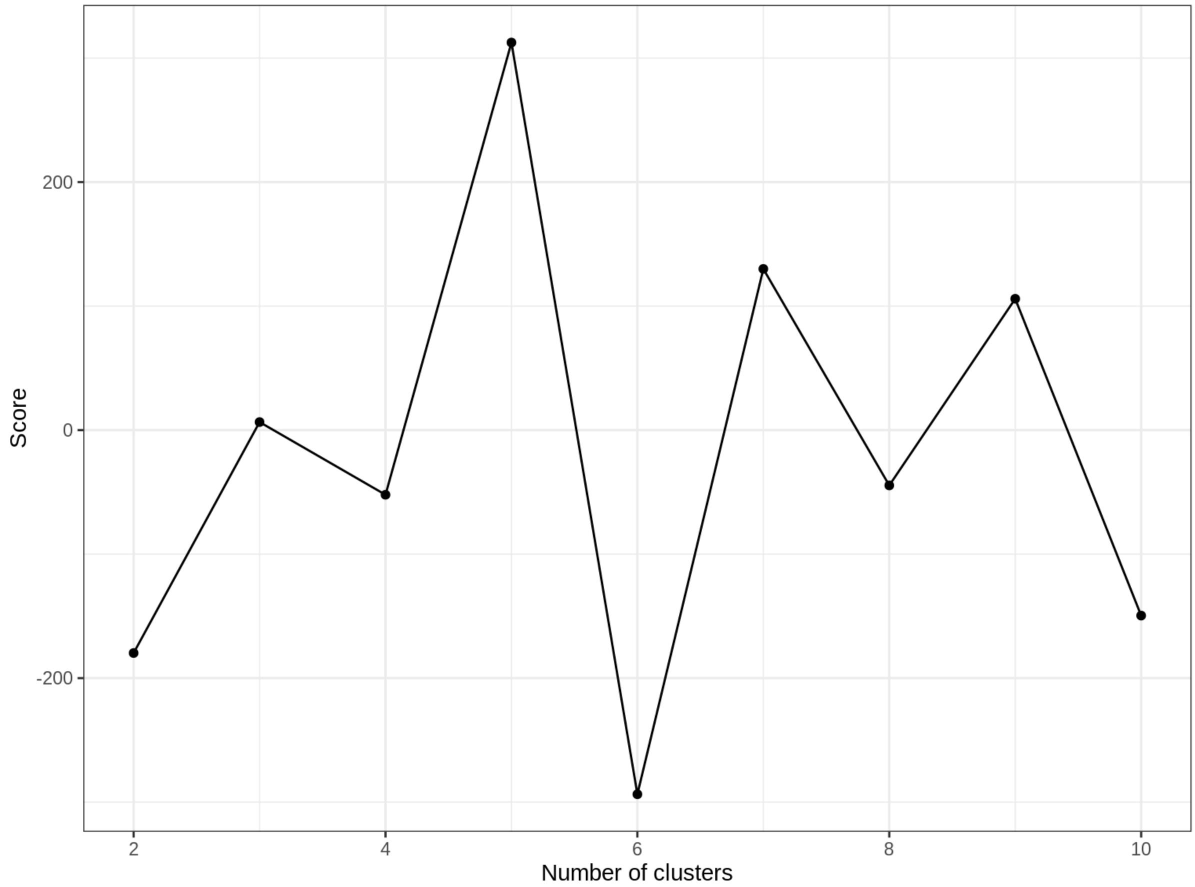

Supplementary Figure 2 Scoring of SIMLR clustering outputs inferred using different numbers of clusters. This comparison suggests the optimal number of clusters for categorising the datasets is six.

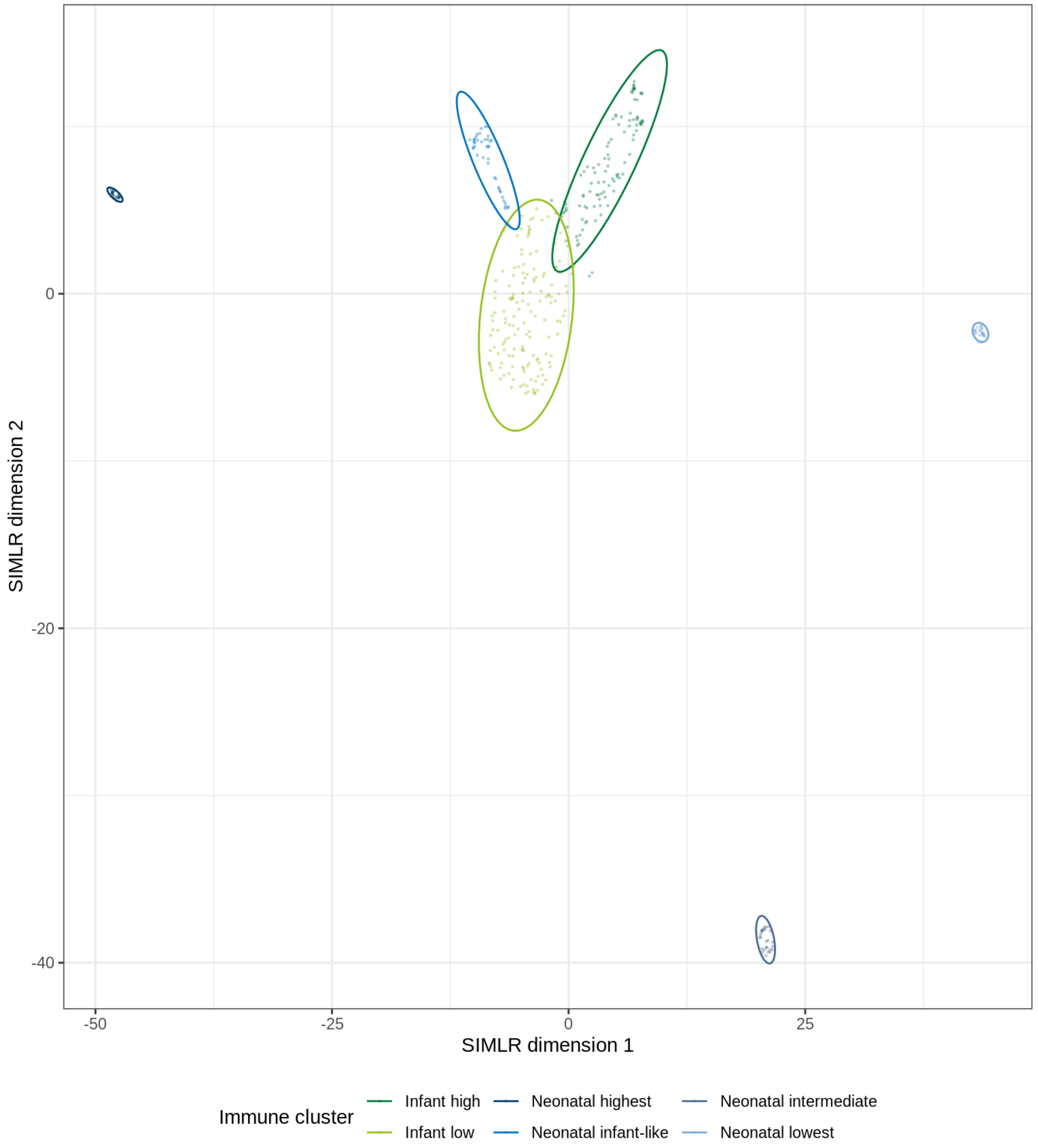

Supplementary Figure 3 Scatterplot showing the SIMLR projection of all serological datasets. Points are coloured according to the cluster to which they belong. The ellipses group together all points belonging to the same cluster.

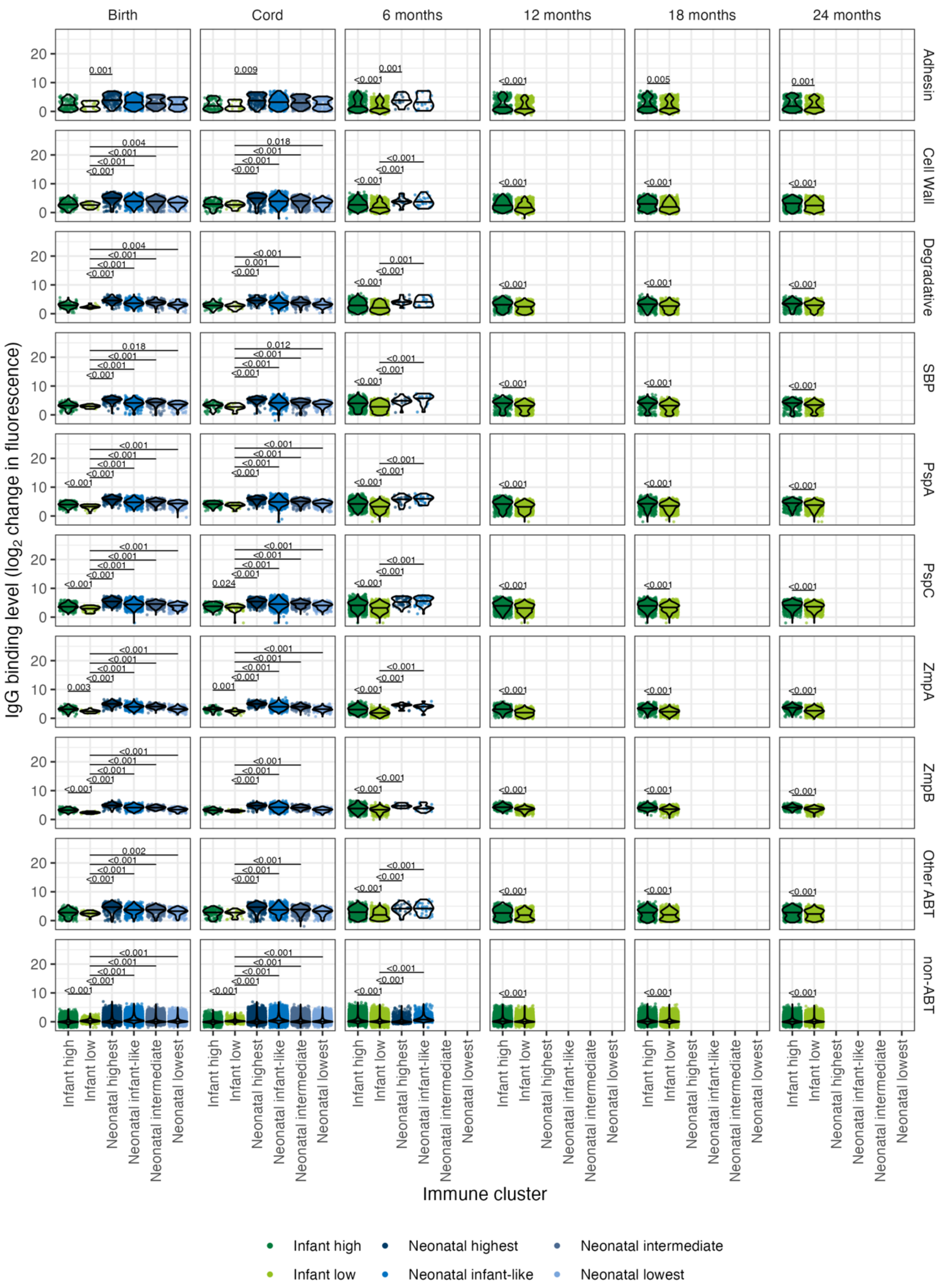
Birth
Cord
6 months
12 months
18 months
24 months
Adhesin
Cell Wall
Degradative
SBP
PspA
PspC
ZmpA
ZmpB
Other ABT
non-ABT
IgG binding level (log_2 change in fluorescence)
Infant high
Infant low
Neonatal highest
Neonatal infant-like
Neonatal intermediate
Neonatal lowest
Immune cluster

Supplementary Figure 4 Violin plots showing the differences in IgG binding between samples assigned to different clusters. Each row of plots corresponds to a different functional antigen class. Each column of plots corresponds to a different sampling age. Each point corresponds to a measurement of IgG binding to a protein in an individual at a particular age. The horizontal line within each violin shows the median. The significance of differences between the measurements in each plot was calculated using a Wilcoxon rank sum test, with a Holm-Bonferroni correction for multiple testing across all panels. These pairwise tests for significant differences used the infant low cluster as the reference set against which the other clusters were compared.

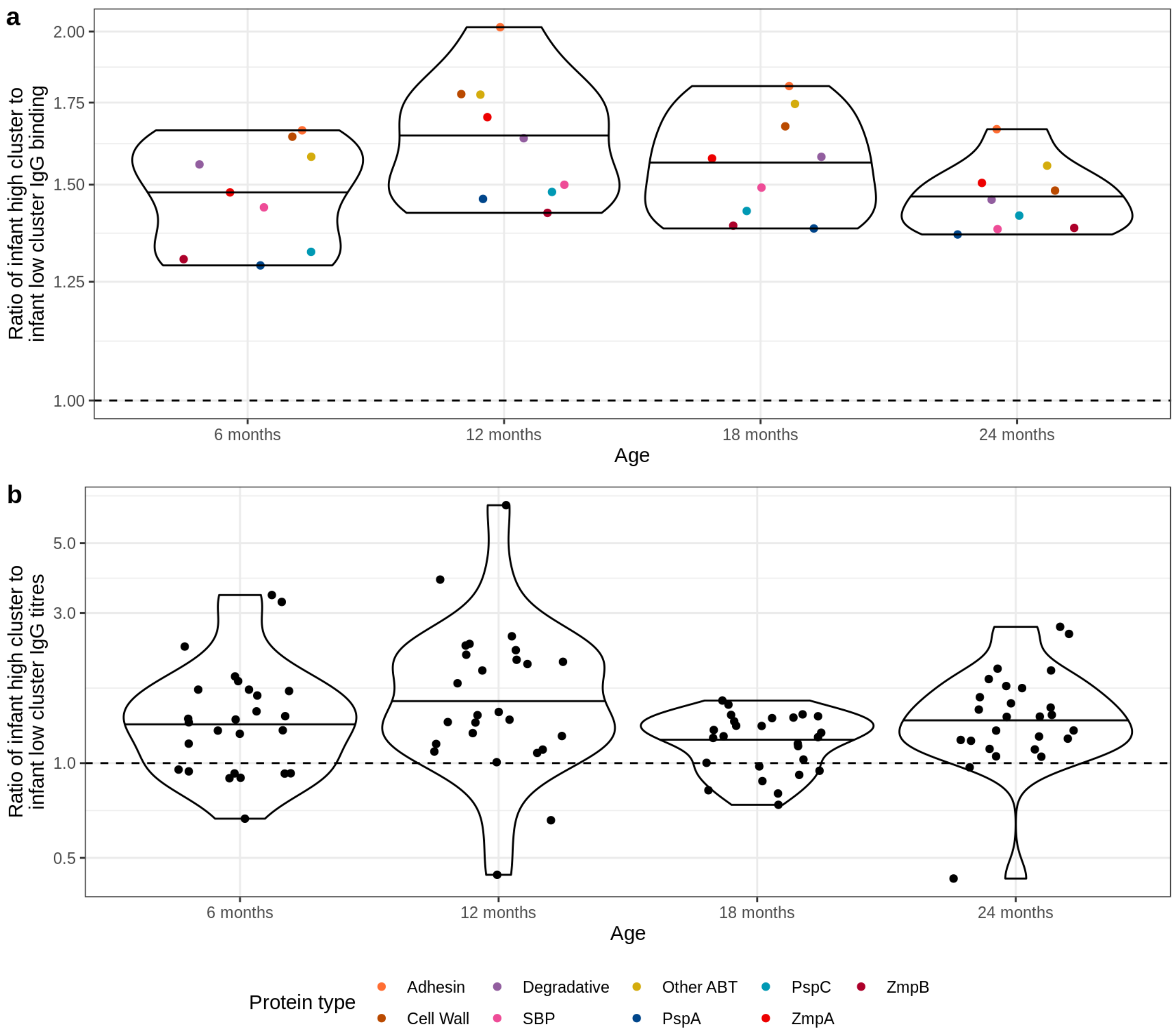

Supplementary Figure 5 Comparison of the levels of IgG responses in the infant high and infant low clusters across all post-birth timepoints. (a) Comparison of IgG levels using array data. Each point shows the ratio of IgG binding in the infant high cluster relative to the infant low cluster at a specified age for a defined ABT functional type, as indicated by the colour of the point. These values were calculated from the linear mixed effects modelling of the relationship between IgG data and immune cluster (Supplementary Table 1) and are summarised for each age by a violin plot, which indicates the median value with a horizontal line. The horizontal dashed line represents a ratio of one, showing the null expectation if the IgG binding in both clusters were equal. (b) Comparisons of IgG levels using ELISA assays. A linear mixed effects model, with the same structure as model 13 in Supplementary Table 1, was fitted to the ELISA data, treating each assay as a separate antigen type. The ratio of the estimated IgG titres for the infant high and infant low clusters (using the same classification defined using the array data) is shown by individual points, and summarised as a violin plot. The horizontal line in each violin shows the median ratio. The horizontal dashed line represents a ratio of one.

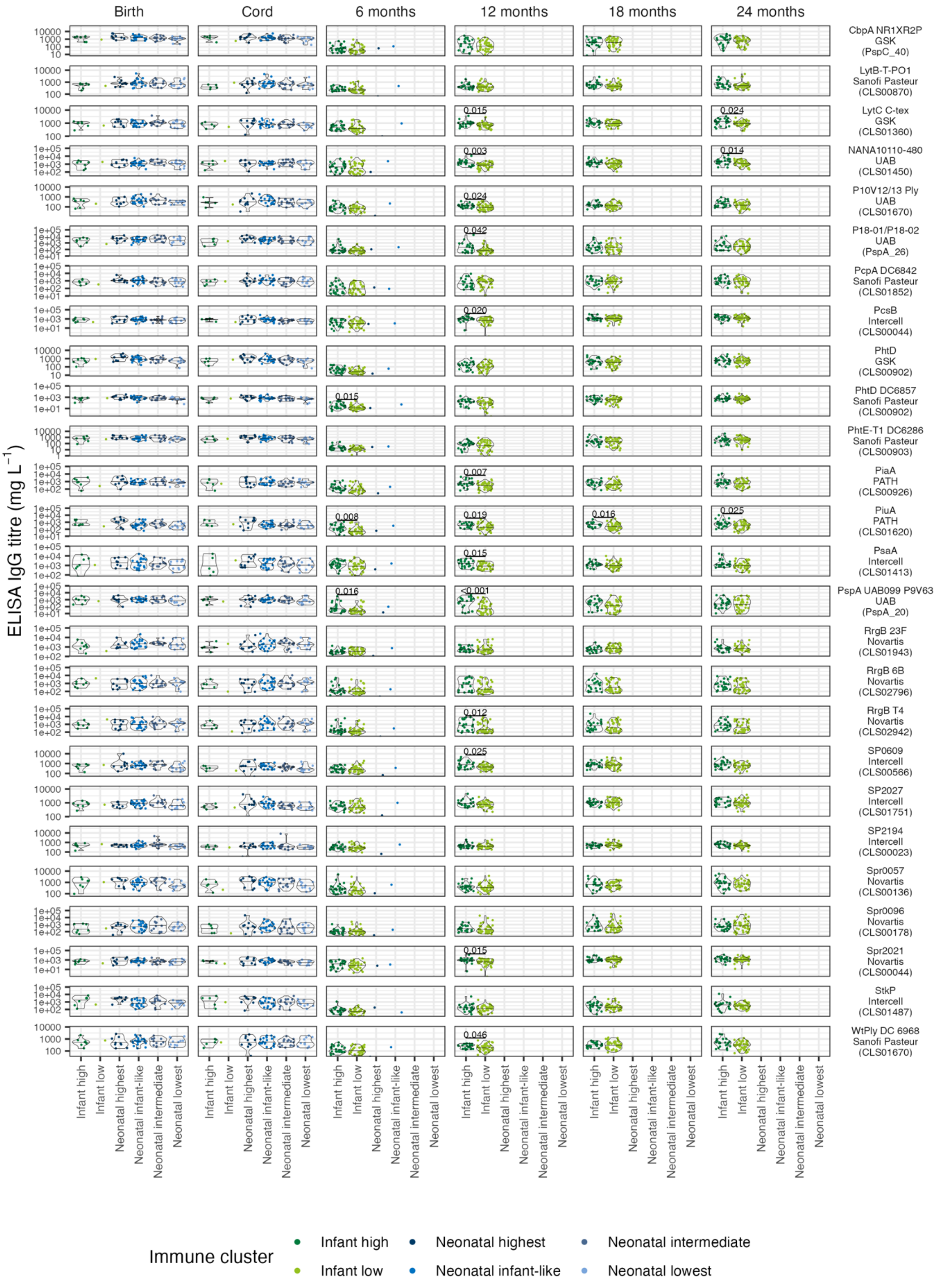
Birth
Cord
6 months
12 months
18 months
24 months
ELISA IgG titre (mg L^{-1})
CbpA NR1XR2P GSK (PspC_40)
LytB-T-PO1 Sanofi Pasteur (CLS00870)
LytC C-tex GSK (CLS01360)
NANA10110-480 UAB (CLS01450)
P10V12/13 Ply UAB (CLS01670)
P18-01/P18-02 UAB (PspA_26)
PcpA DC6842 Sanofi Pasteur (CLS01852)
PcsB Intercell (CLS00044)
PhtD GSK (CLS00902)
PhtD DC6857 Sanofi Pasteur (CLS00902)
PhtE-T1 DC6286 Sanofi Pasteur (CLS00903)
PiaA PATH (CLS00926)
PiuA PATH (CLS01620)
PsaA Intercell (CLS01413)
PspA UAB099 P9V63 UAB (PspA_20)
RrgB 23F Novartis (CLS01943)
RrgB 6B Novartis (CLS02796)
RrgB T4 Novartis (CLS02942)
SP0609 Intercell (CLS00566)
SP2027 Intercell (CLS01751)
SP2194 Intercell (CLS00023)
Spr0057 Novartis (CLS00136)
Spr0096 Novartis (CLS00178)
Spr2021 Novartis (CLS00044)
StkP Intercell (CLS01487)
WtPly DC 6968 Sanofi Pasteur (CLS01670)
Infant high
Infant low
Neonatal highest
Neonatal infant-like
Neonatal intermediate
Neonatal lowest
Immune cluster

Supplementary Figure 6 Validation of sample clustering using ELISA data. Serum samples were classified according to the clustering inferred from the array data, shown in Fig. 1. Each plot shows the responses to a specific ELISA assay antigen at sampled ages. Each point represents the IgG responses to the specified protein in an individual. To enable plotting on a logarithmic scale, IgG titres of zero were converted to half the minimum non-zero value recorded across the dataset. The violin plots summarise the responses within each immune cluster. The horizontal lines within the violins show the median response of each cluster. The significance of differences between the measurements in each plot was calculated using a Wilcoxon rank sum test, with a Holm-Bonferroni correction for multiple testing applied across all comparisons.

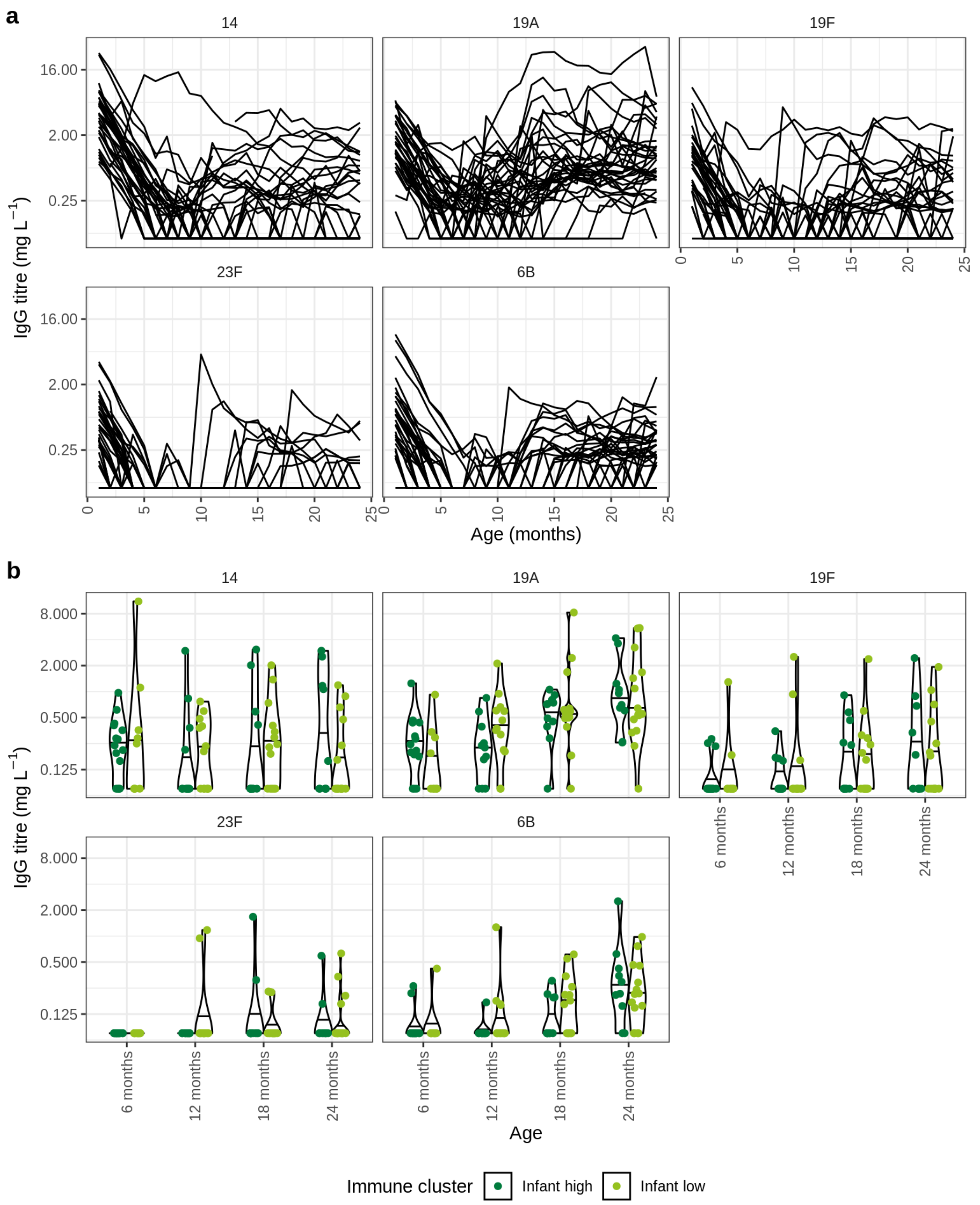

Supplementary Figure 7 Individuals' responses to pneumococcal capsular antigens. Turner *et al* previously measured the titres of IgG binding to five common pneumococcal capsular antigens using ELISA assays. (a) Line plots showing the responses to the five capsular antigens in each individual, measured at monthly intervals. (b) Violin plots showing the response to capsular antigens categorised by the immune clusters defined using the array data. Each point represents a response to a capsular antigen in an individual. Points are grouped and coloured according to the assignment of individuals to immune clusters, with their distribution summarised by a violin plot. The horizontal line within the violin shows the median IgG titre.

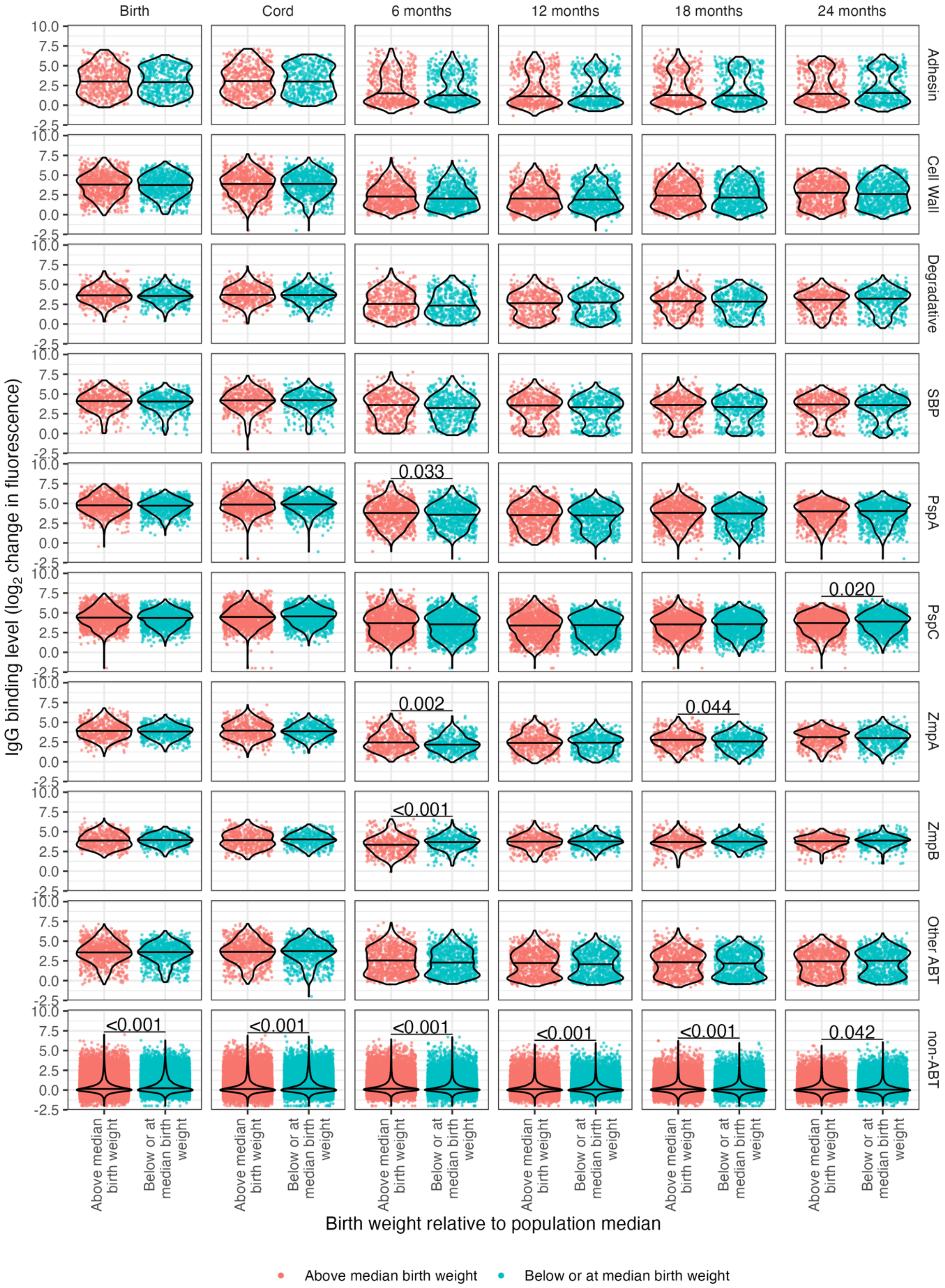

Supplementary Figure 8 Violin plots showing the differences in IgG binding to proteins between children born with a weight under the median for the cohort, relative to those born with a weight over the median for the cohort. Data are shown as in Fig. 5.

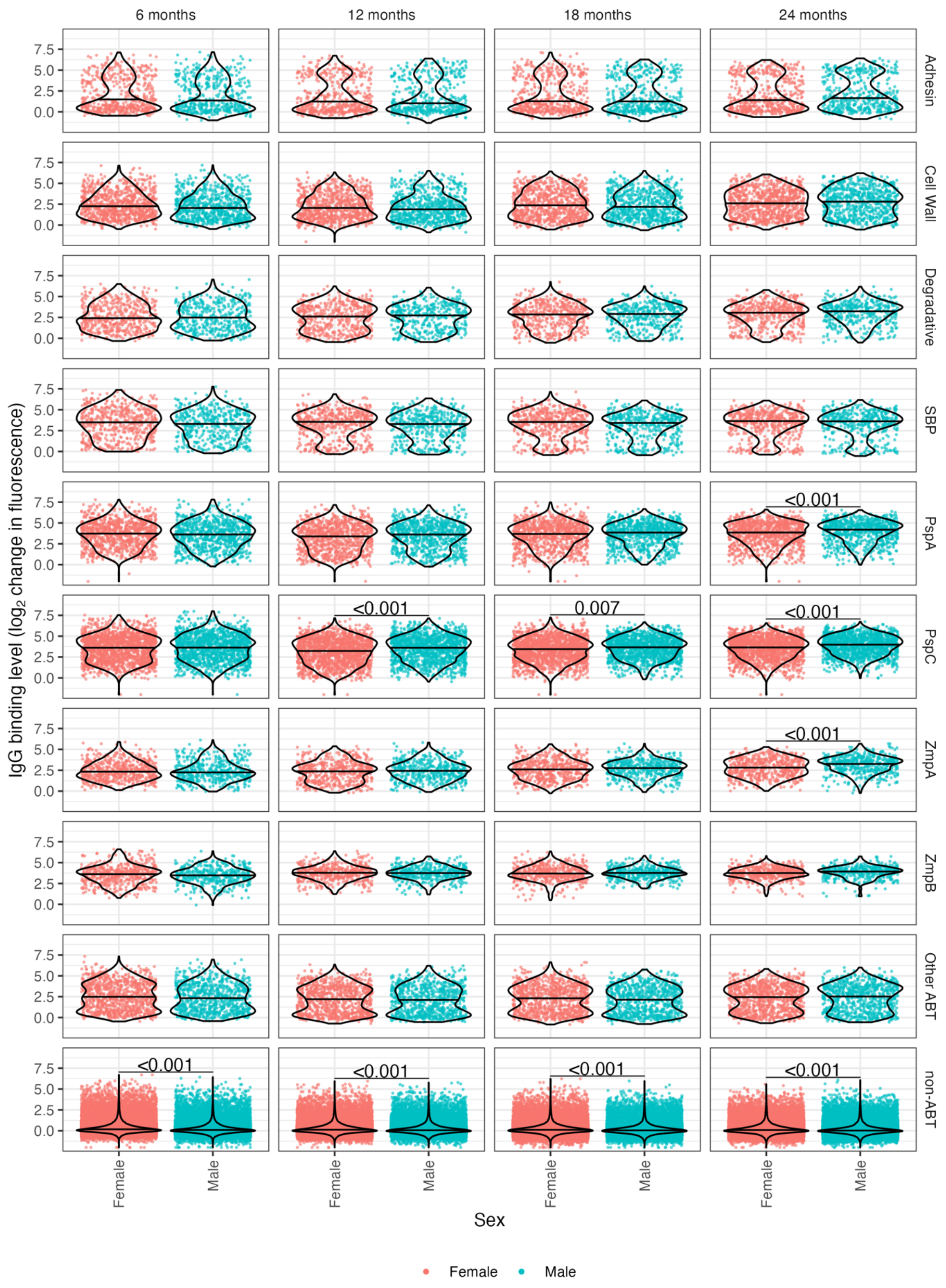

Supplementary Figure 9 Violin plots showing the differences in IgG binding to proteins between females and males. Data are shown as in Fig. 5.

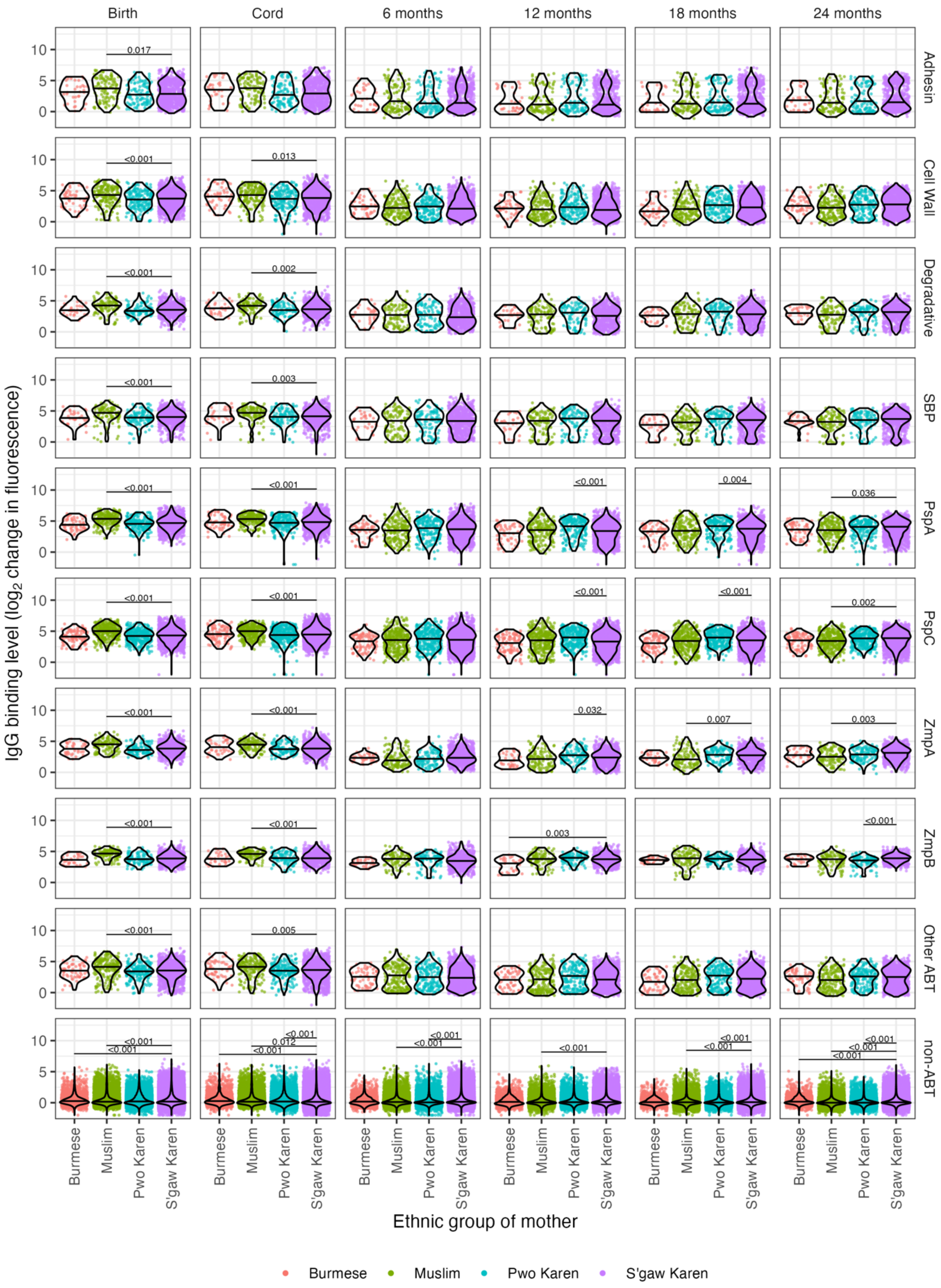

Supplementary Figure 10 Violin plots showing the differences in IgG binding to proteins between children born to mothers of different ethnicities. The pairwise tests for significant differences used the S’gaw Karen ethnicity as the reference set against which the other ethnicities were compared. Data are shown as in Fig. 5.

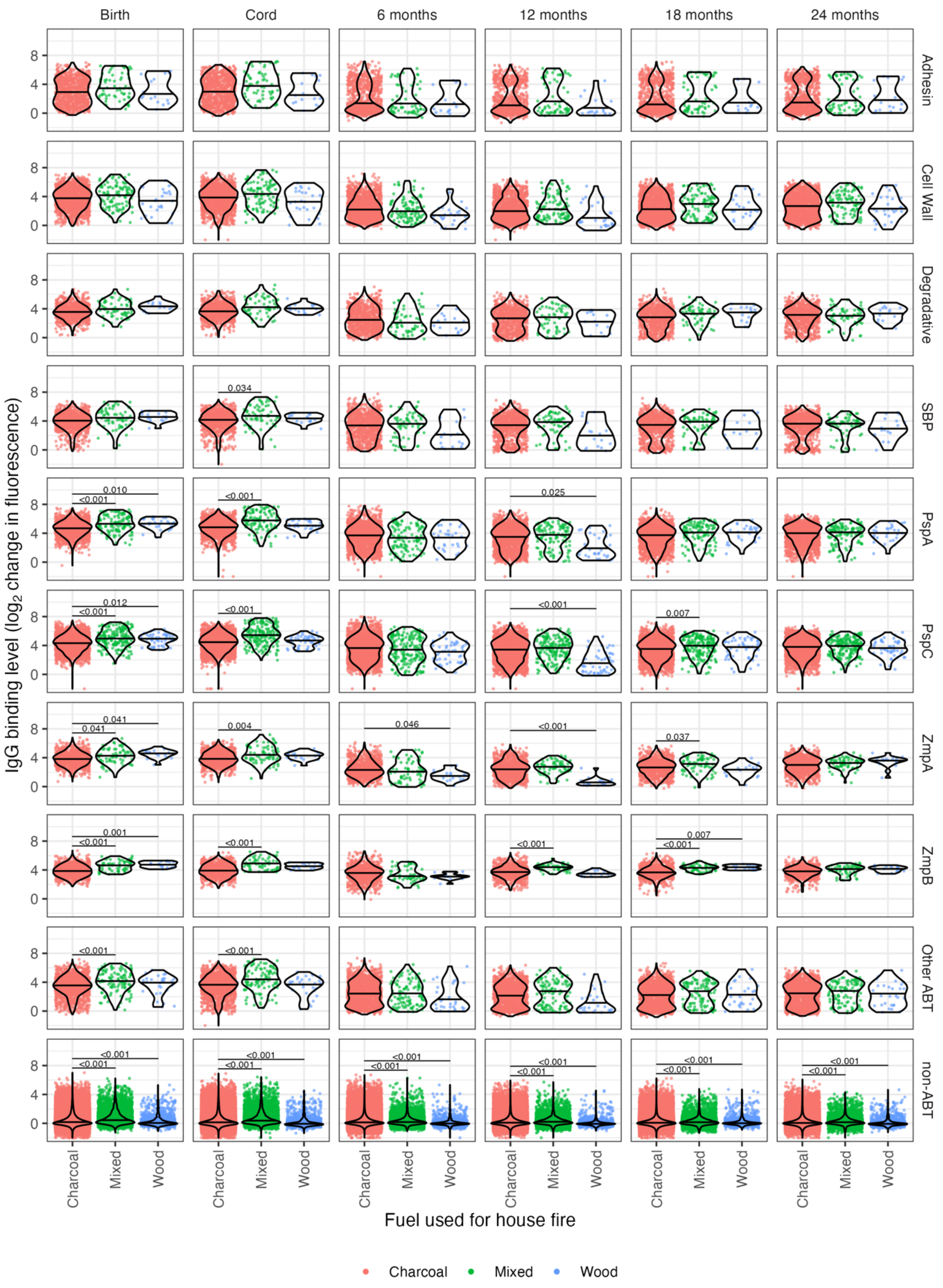

Supplementary Figure 11 Violin plots showing the differences in IgG binding to proteins between children born in households heated by fires using different fuel types. The pairwise tests for significant differences used charcoal fuel as the reference set against which the other fuels were compared. Data are shown as in Fig. 5.

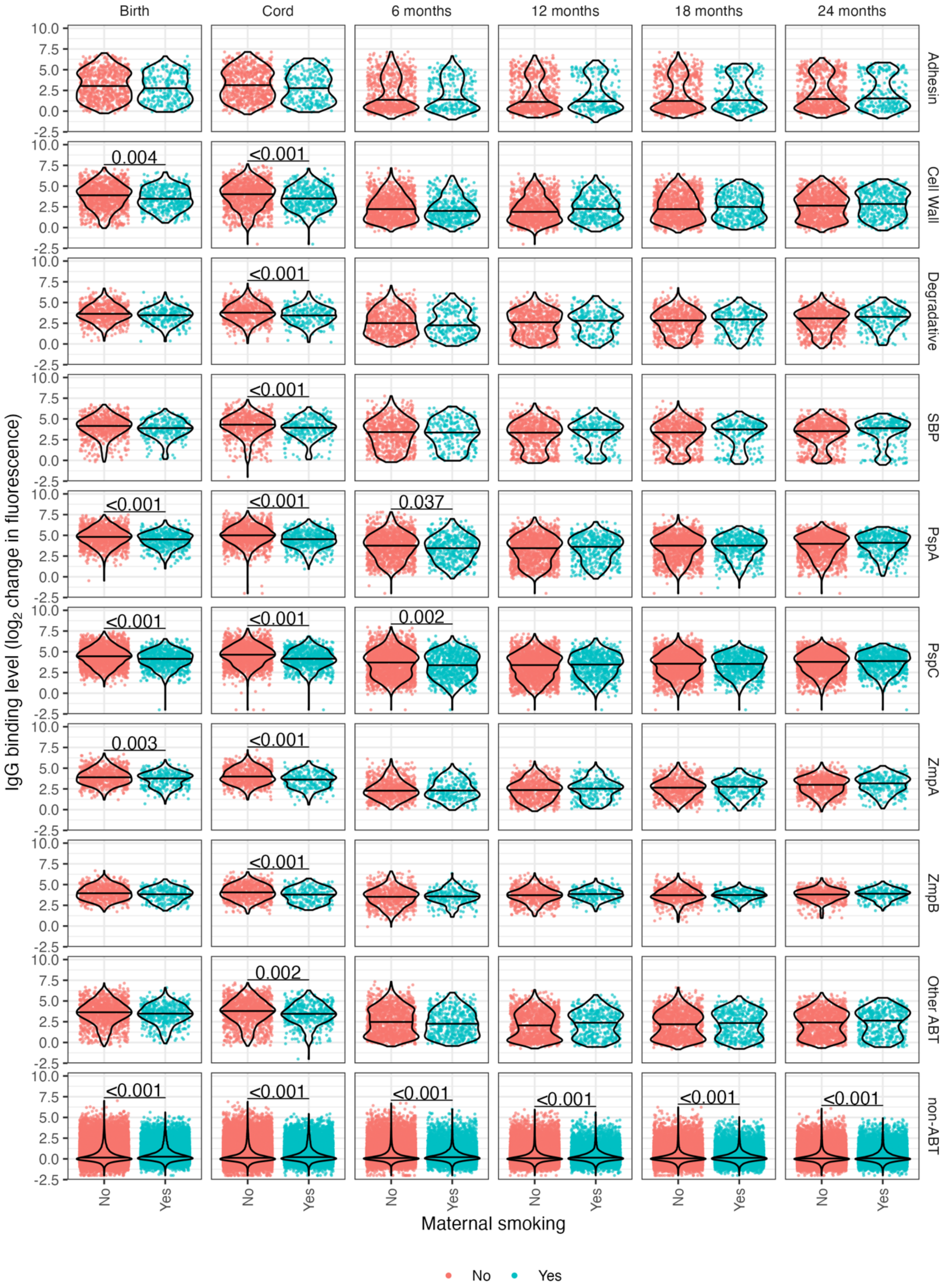

Supplementary Figure 12 Violin plots showing the differences in IgG binding to proteins between children born to smoking or non-smoking mothers. Data are shown as in Fig. 5.

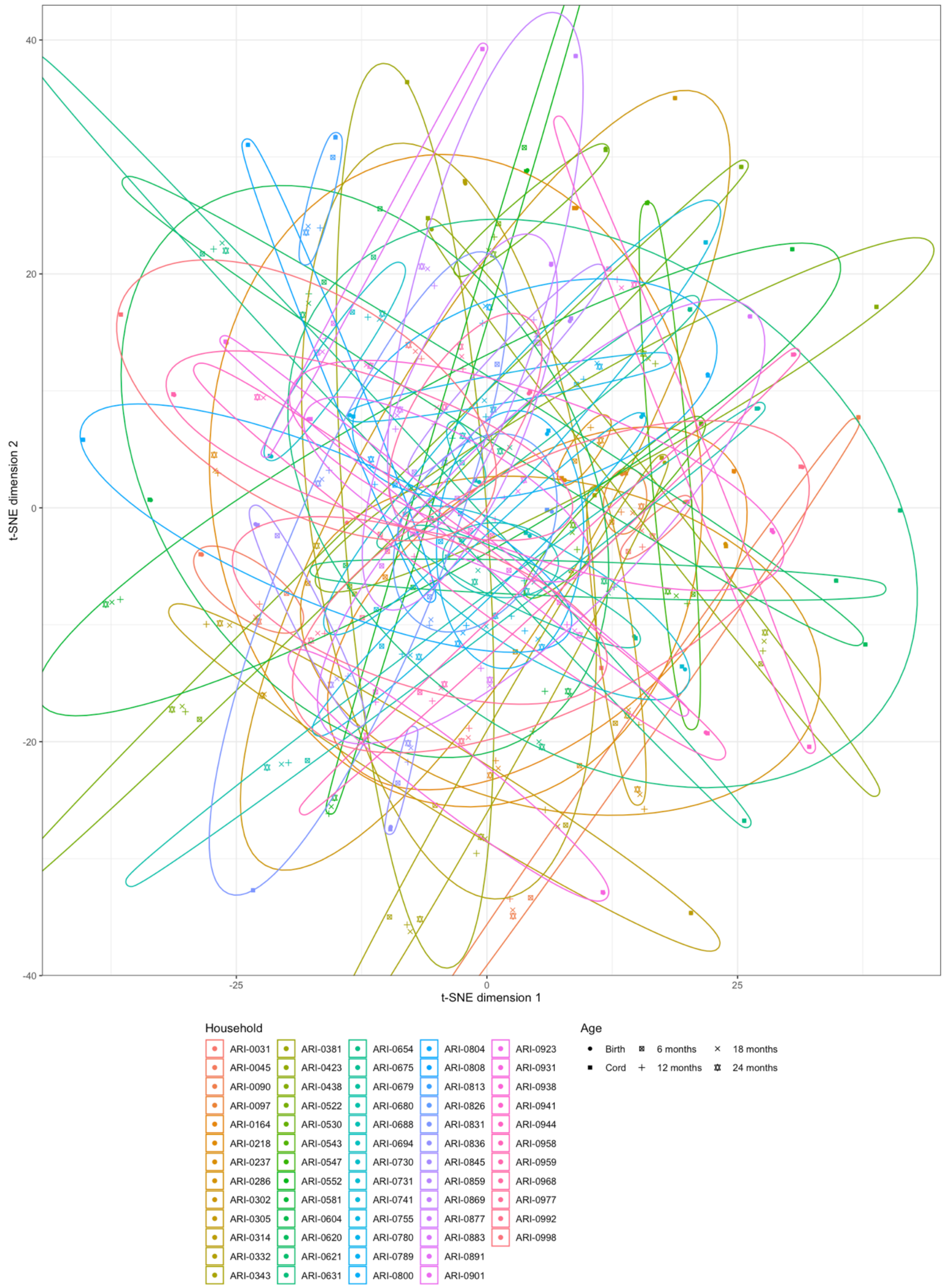

Supplementary Figure 13 Scatterplot showing a t-SNE projection of all serological datasets. The shape of each point shows the timepoint at which the corresponding sample was collected. Each set of points from an individual are coloured the same and grouped together by an ellipse. The embedding was calculated with a limit of 50,000 iterations.

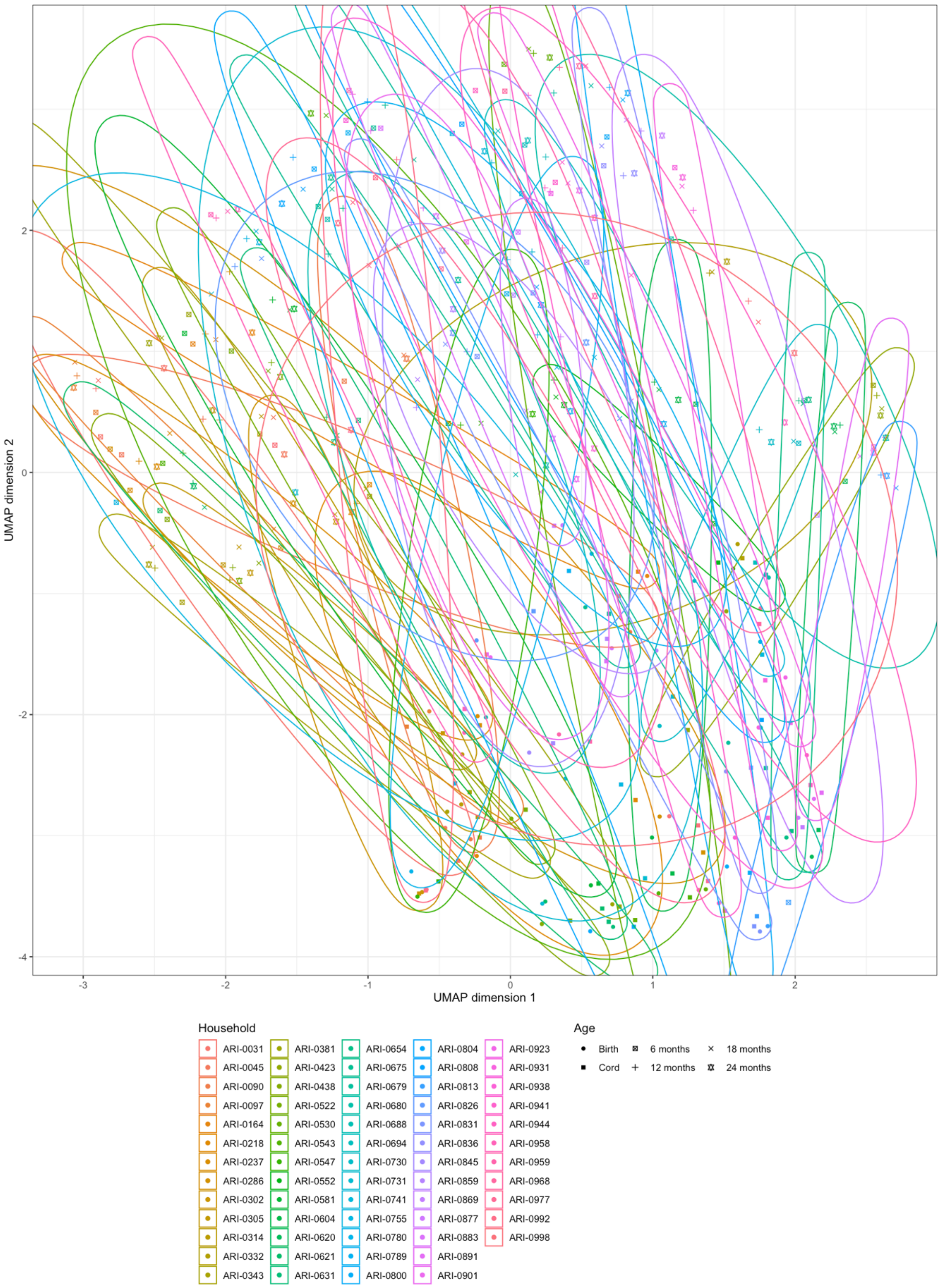

Supplementary Figure 14 Scatterplot showing a UMAP projection of all serological datasets. The shape of each point shows the timepoint at which the corresponding sample was collected. Each set of points from an individual are coloured the same and grouped together by an ellipse. This shows the inability of t-SNE to resolve individuals from one another is not particular to that specific method of calculating a two-dimensional projection. The embedding was calculated with a limit of 500 iterations.

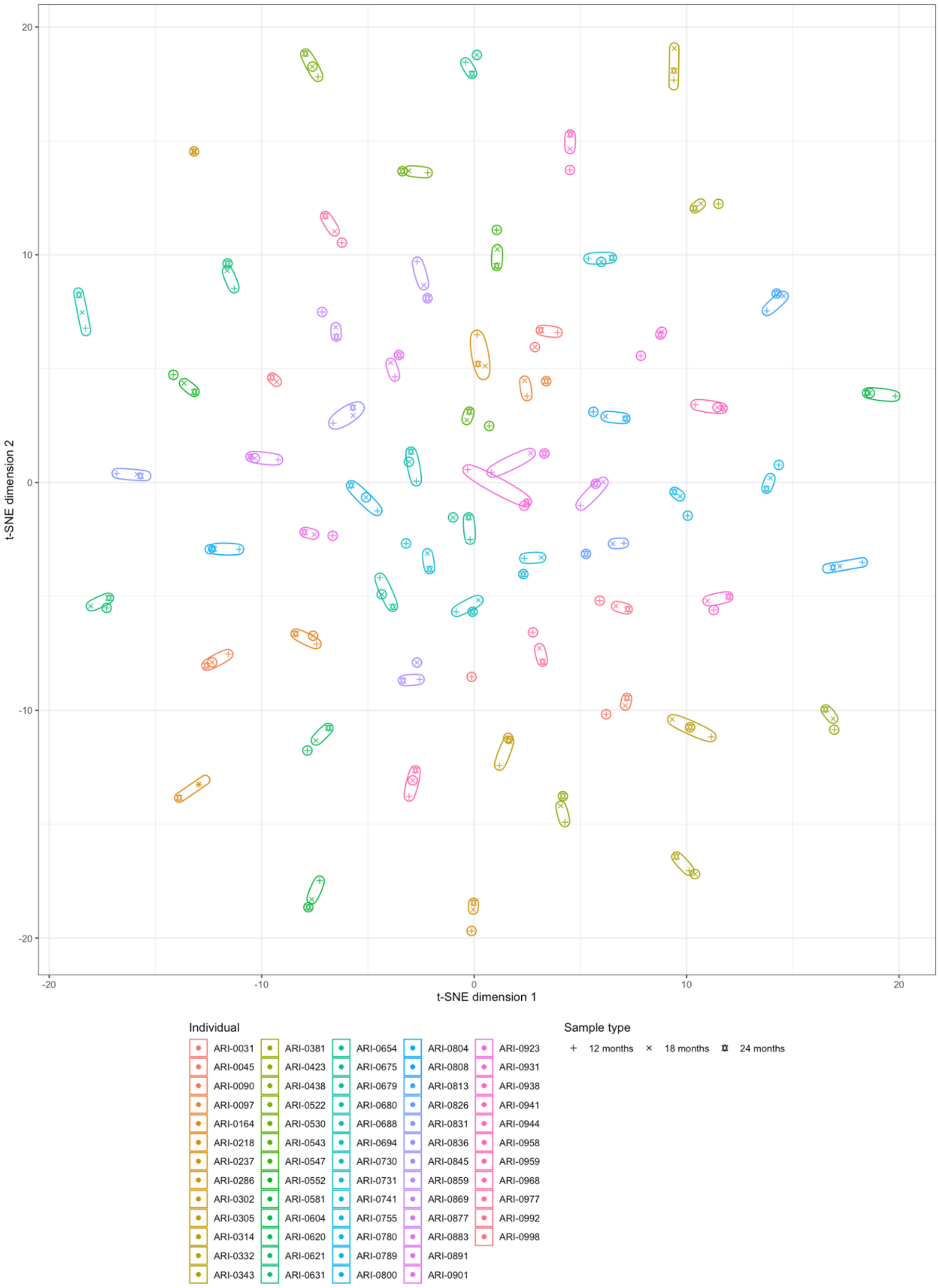

Supplementary Figure 15 Scatterplot showing a t-SNE projection of serological datasets collected from children 12 mo and older. The shape of each point shows the timepoint at which the corresponding sample was collected. The colour represents the individual from whom the sample was collected. Each set of points measured using the same panproteome slide is grouped together by an ellipse. This shows that highly similar points, coming from the same individual, are sometimes derived from different slides. This demonstrates the consistency of the measurements from a single individual, relative to the variation between individuals, is not an artefact of technical variation between different batches of slides. The embedding was calculated with a limit of 50,000 iterations.

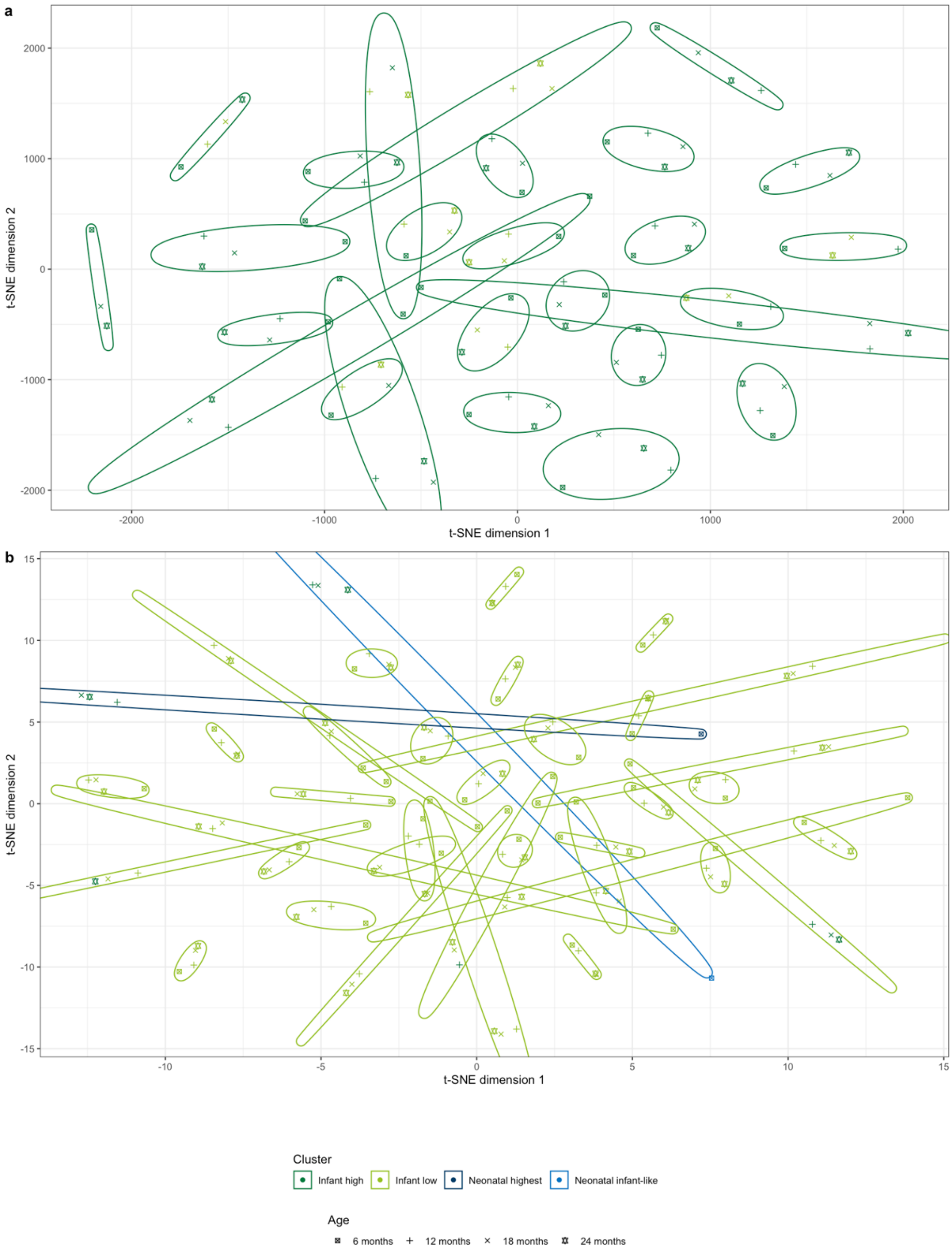

Supplementary Figure 16 Scatterplots showing t-SNE projections of all serological datasets collected from children 6 mo or older. These embeddings were calculated with a limit of 50,000 iterations. The datasets were divided by whether they were assigned to (a) the "infant high" cluster at 6 mo, or (b) any other cluster. The shape of each point shows the timepoint at which the corresponding sample was collected. Each set of points from an individual are grouped together by an ellipse. This plot demonstrates many of the samples taken from 6 mo children cluster with samples taken subsequently from the same individual, indicating the unique immune profile of some children developed by 6 mo. However, the 6 mo sample was divergent from late samples in a minority of children, suggesting the endogenous immune response was still in the early stages of development.

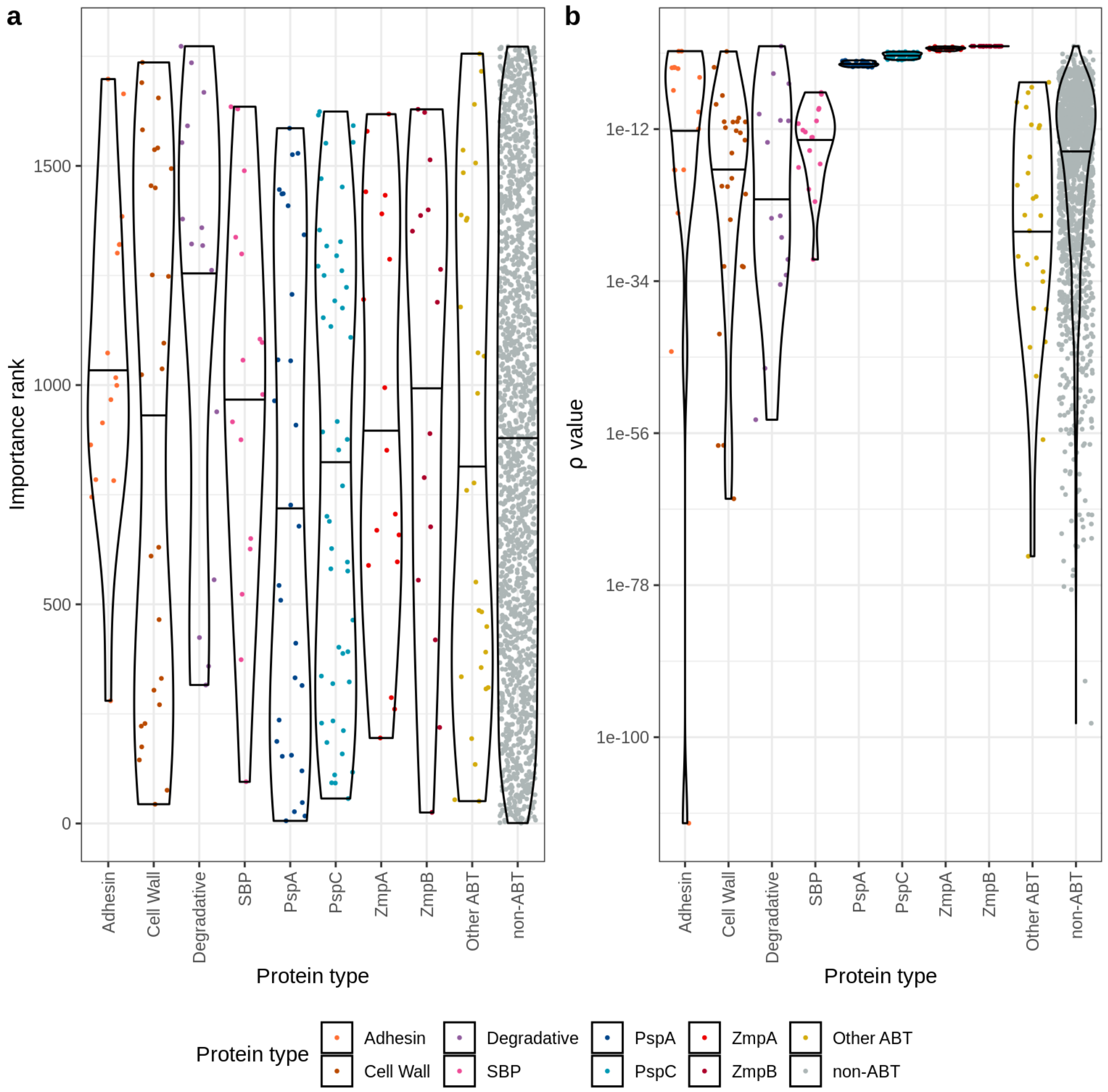

Supplementary Figure 17 Contribution of different protein types to the SIMLR clustering. (a) Violin plot showing the ranked importance of each protein to the clustering. The lower-ranked proteins make a greater contribution to the classification of the samples. This demonstrates the clustering is supported similarly strongly across all functional classes. (b) Violin plot showing the significance of ranks, as quantified by rho scores calculated using robust rank aggregation. These small values indicate the differences between ranks are unlikely to be due to random chance, suggesting the clustering is not the consequence of noise in the data.

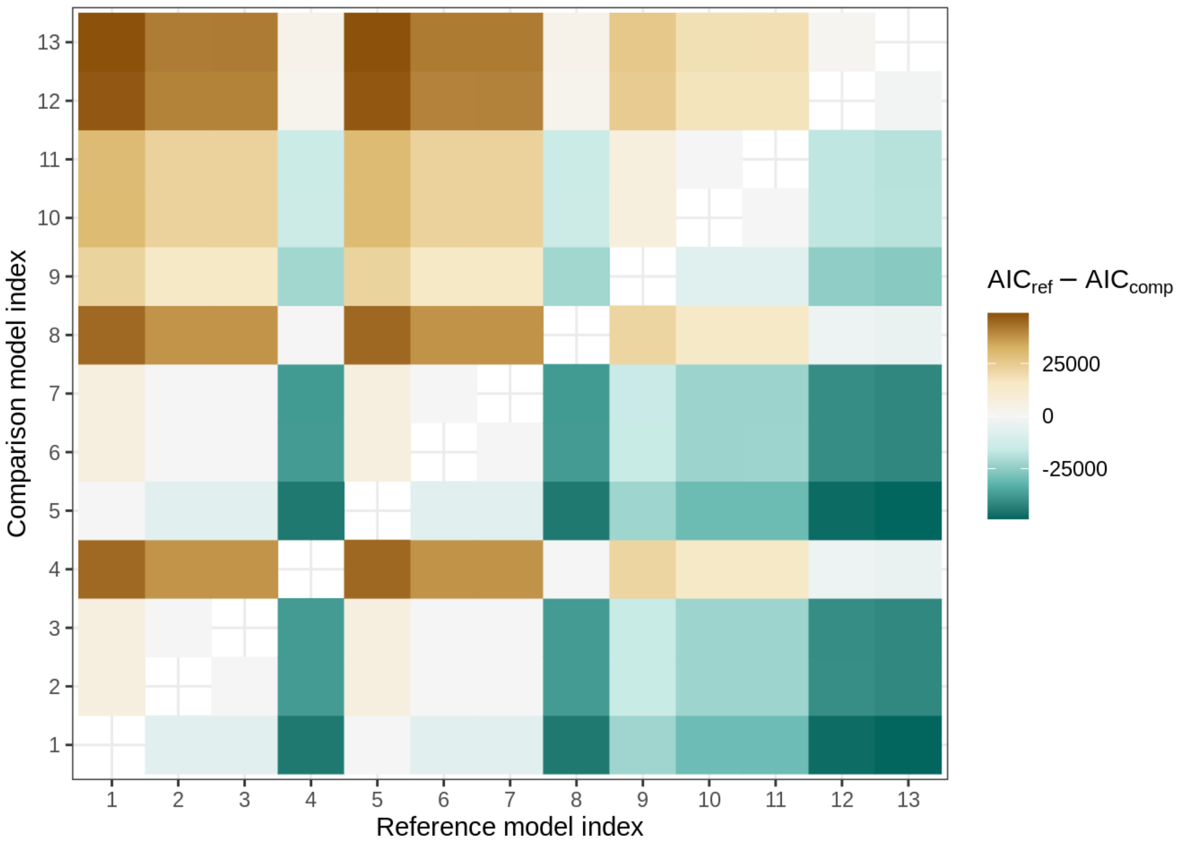

Supplementary Figure 18 Heatmap showing the difference in AIC between linear mixed effects models fitted to the IgG binding data. The indices relate to the models defined in Supplementary Table 1. The colour of each cell shows $AIC_{reference} - AIC_{comparison}$, such that negative values correspond to the reference model having the lower AIC, and therefore being the most appropriate model for the data.

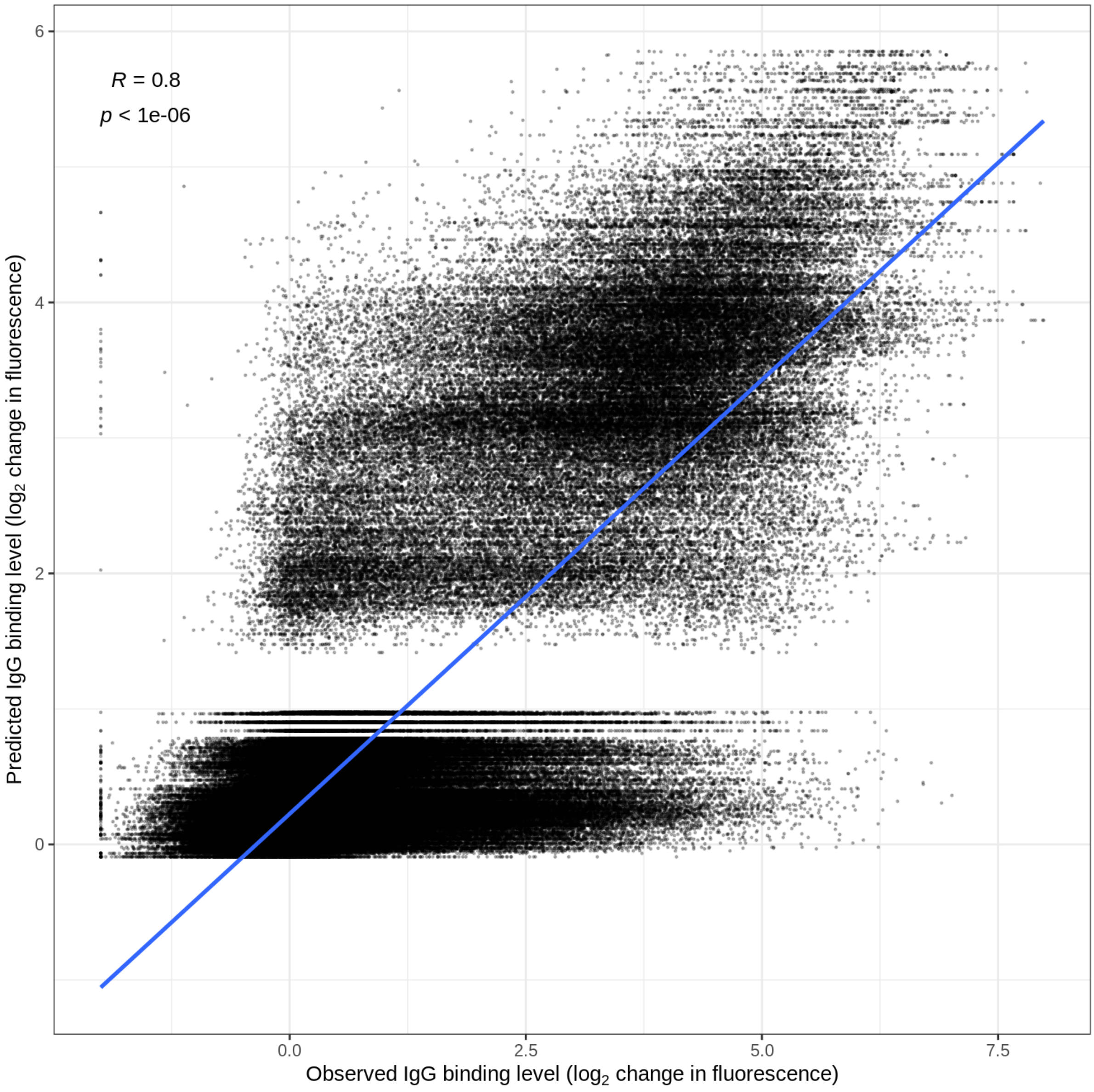

Supplementary Figure 19 Scatterplot showing the relationship between the observed IgG binding levels and those predicted by the best-fitting linear mixed-effects model. The best-fitting linear relationship between the observed and predicted values is shown by the blue line.

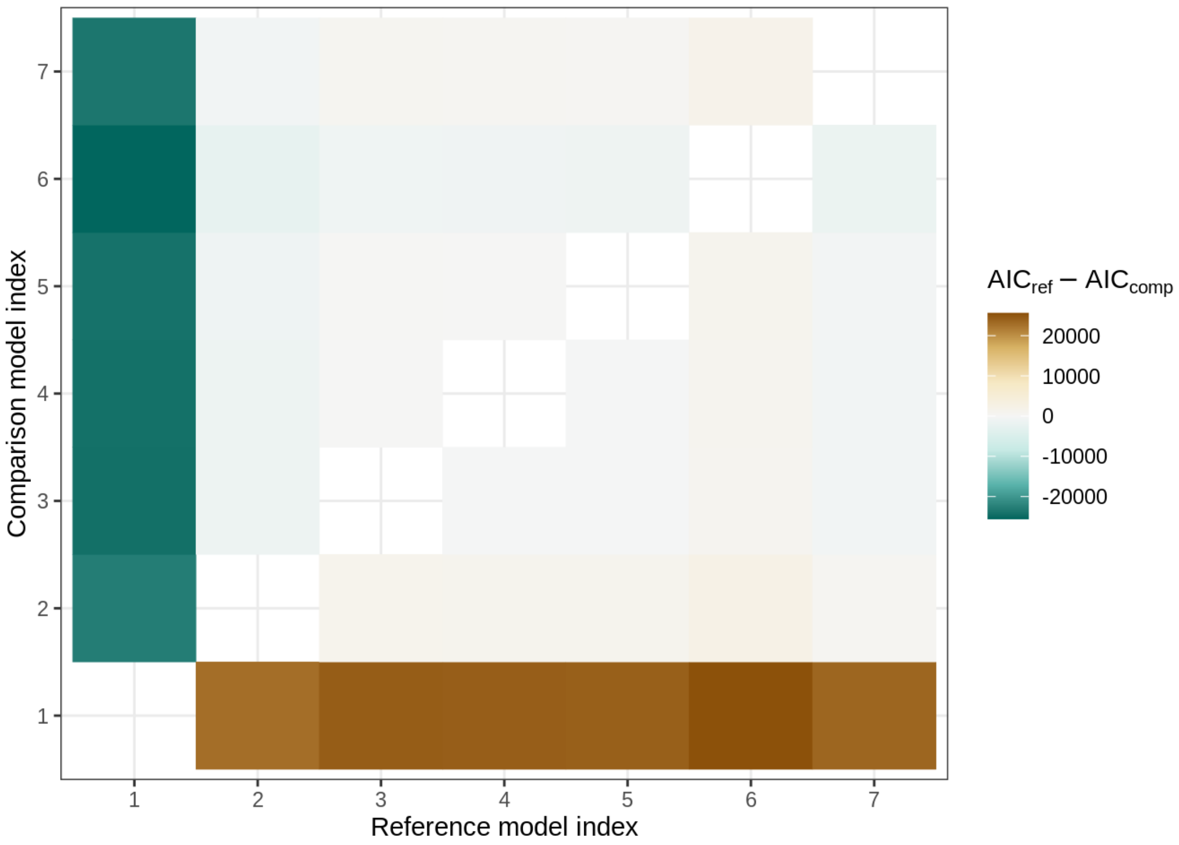

Supplementary Figure 20 Heatmap showing the difference in AIC between linear mixed effects models fitted to the IgG binding data. The indices relate to the models defined in Supplementary Table 2. Data are shown as described for Supplementary Figure 18.

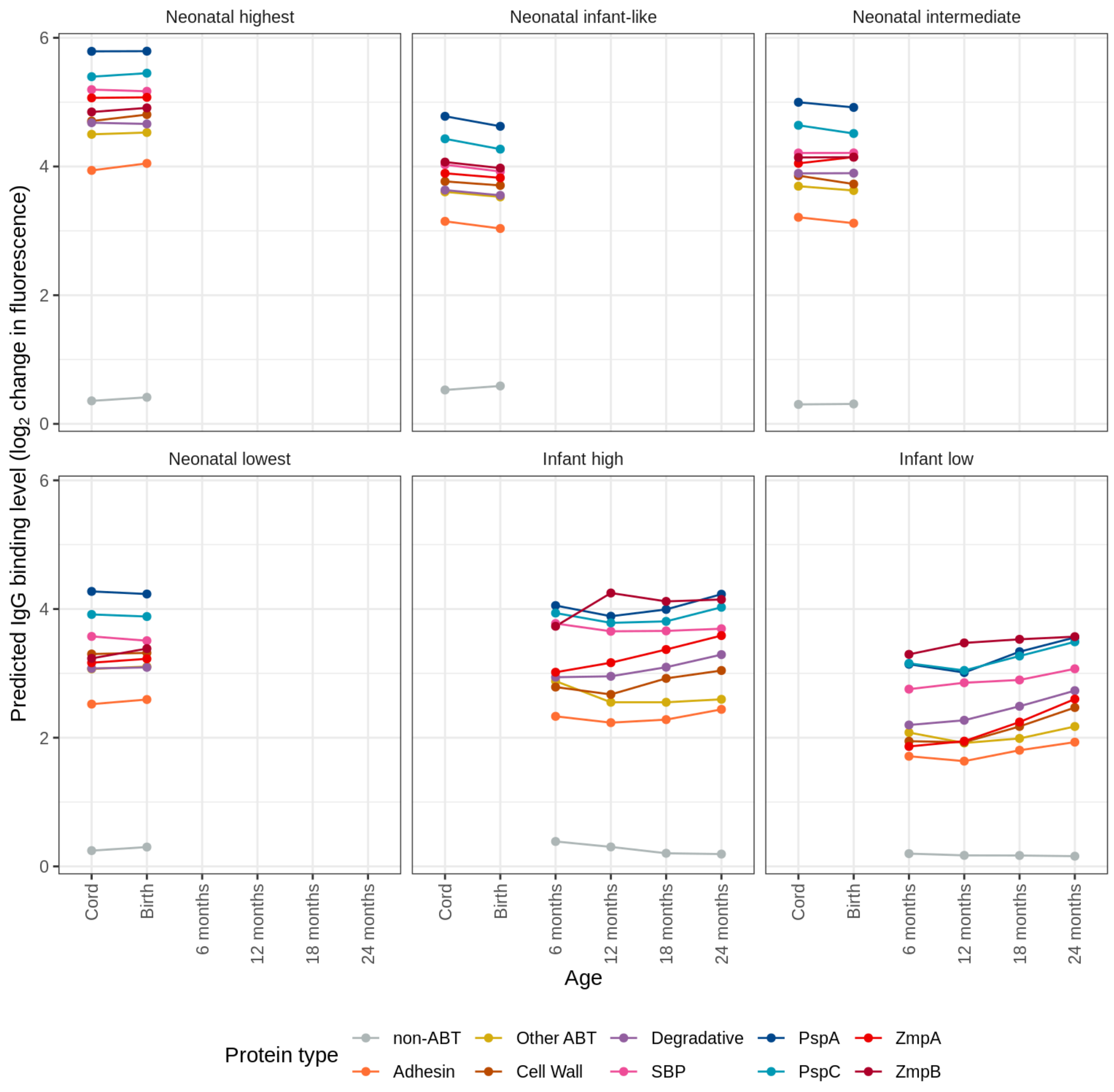

Supplementary Figure 21 Line plots showing the changes in IgG binding to proteins with age inferred by a linear mixed-effects model (Supplementary Table 1), classified by cluster. Each plot displays the age-associated changes in IgG binding to probes corresponding to different protein types, as indicated by the line's colour. Predictions are only shown for the maternal birth and umbilical cord samples, for neonatal clusters, and for the samples taken from children 6 mo or older, for the infant clusters.

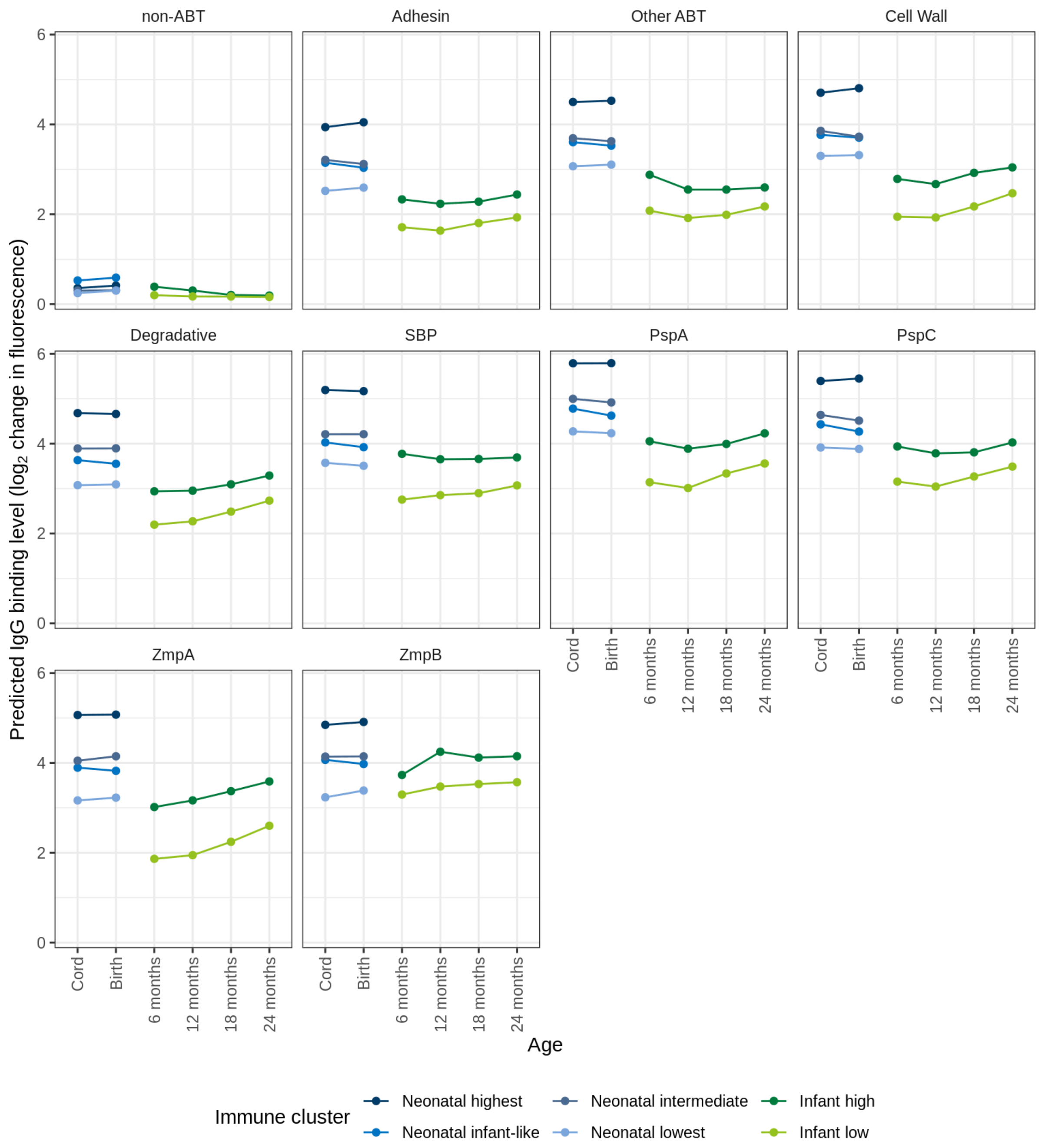

Supplementary Figure 22 Line plots showing the changes in IgG binding to proteins of different functional types with age within each immune cluster. These values were inferred by a linear mixed-effects model (Supplementary Table 1). Predictions are only shown for the maternal birth and umbilical cord samples, for neonatal clusters, and for the samples taken from children 6 mo or older, for the infant clusters.

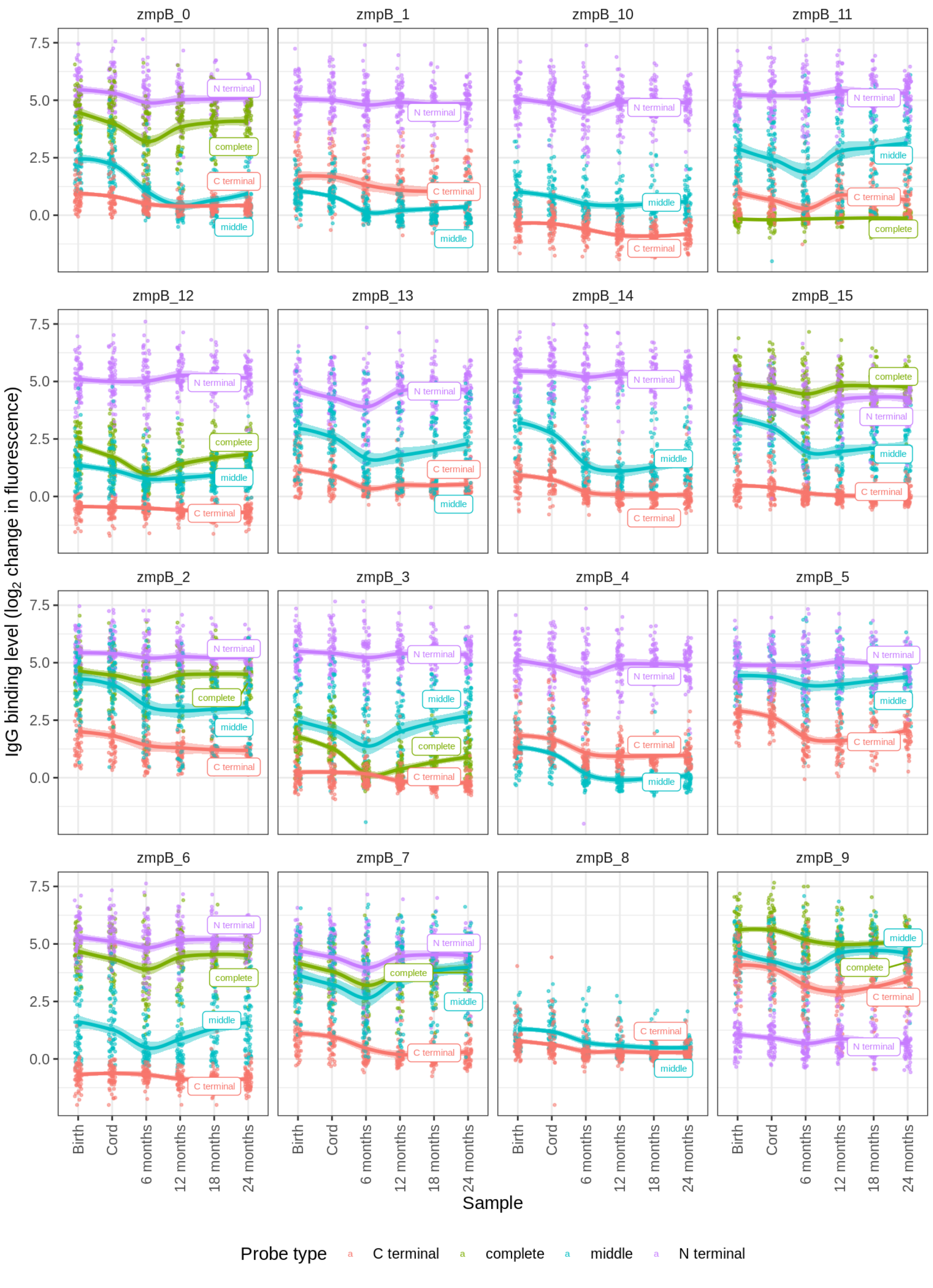

Supplementary Figure 23 Scatterplot detailing the binding of IgG to ZmpB probes. Each point represents the IgG binding to a particular probe at defined sampling time. Each individual plot corresponds to a different ZmpB variant. The colours denote whether the probe corresponded to the N terminal region, middle section, C terminal region, or the complete protein. The lines show the change in IgG binding with age, estimated using a Loess regression. The shaded region shows the 95% confidence interval.

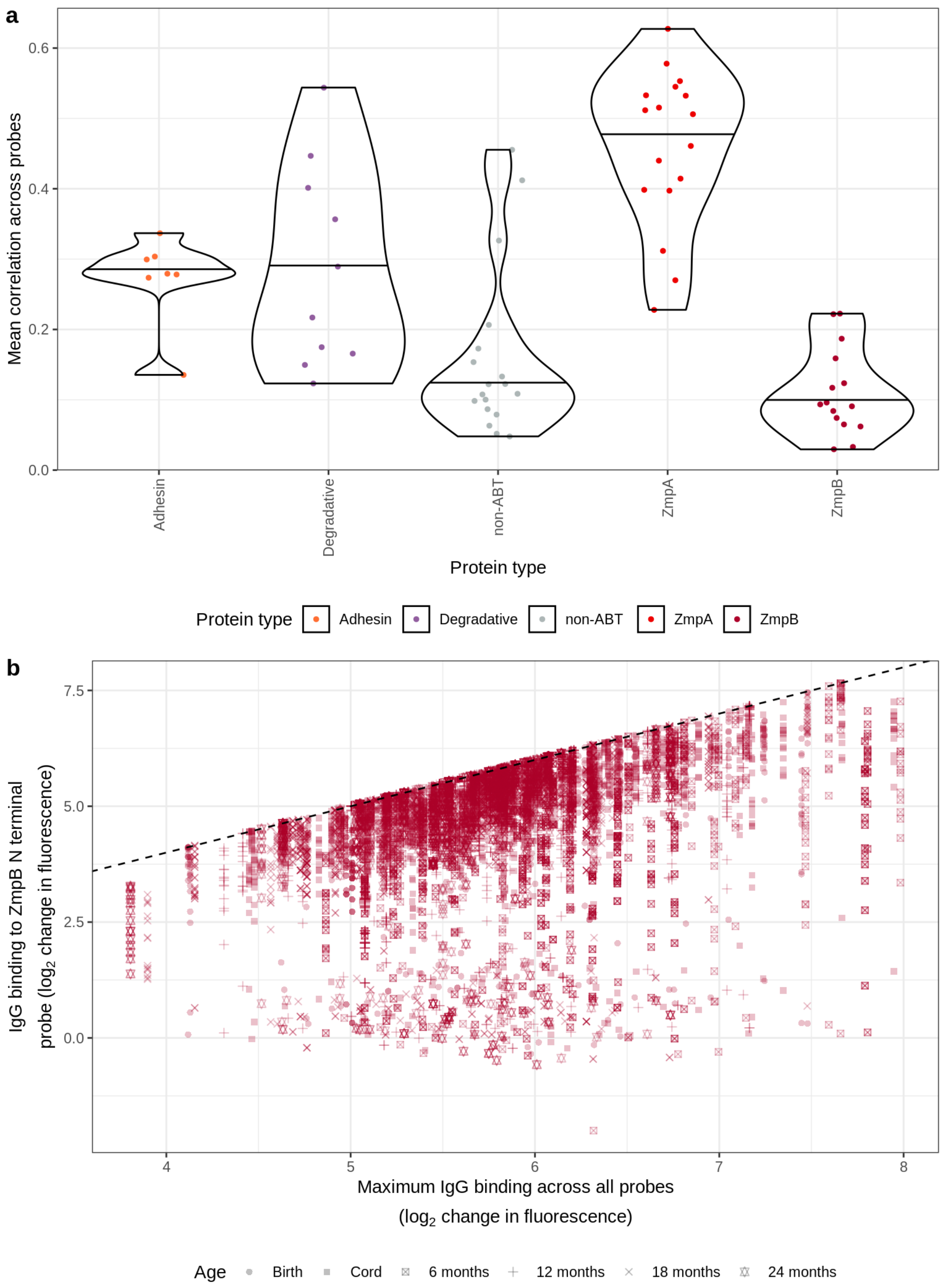

Supplementary Figure 24 IgG binding to ZmpB (a) Violin plot summarising the consistency of IgG binding measurements to polypeptide probes corresponding to partial fragments, or complete versions, of the same protein. Each point corresponds to the mean pairwise Pearson correlation coefficient, *R*, of IgG binding levels across the probes representing an individual protein, after values were grouped by individual. Higher correlation coefficients

indicate the probes provide a consistent measurement of each individual's changing immune response with age. The distribution of correlation coefficients for each protein type are summarised as a violin plot, with a horizontal line marking the median value. The correlation coefficients are generally higher for the ABTs than the non-ABTs, consistent with IgG binding to non-ABTs largely representing background noise that does not exhibit a strong trend relative to age. However, the weakest correlation relative to age are observed for the ZmpB probes, the binding of which to IgG does not exhibit a strong association with the age of the individual at the time of sampling. (b) Comparison of the maximum IgG binding observed across all probes in a dataset, on the horizontal axis, with the IgG binding to ZmpB probes, on the vertical axis. The shape of each point shows the age of the child at the time of sampling. The dashed line corresponds to the line of identity. This shows the maximum IgG binding observed across all probes in a dataset often corresponded to a response to the N terminus of a ZmpB protein.

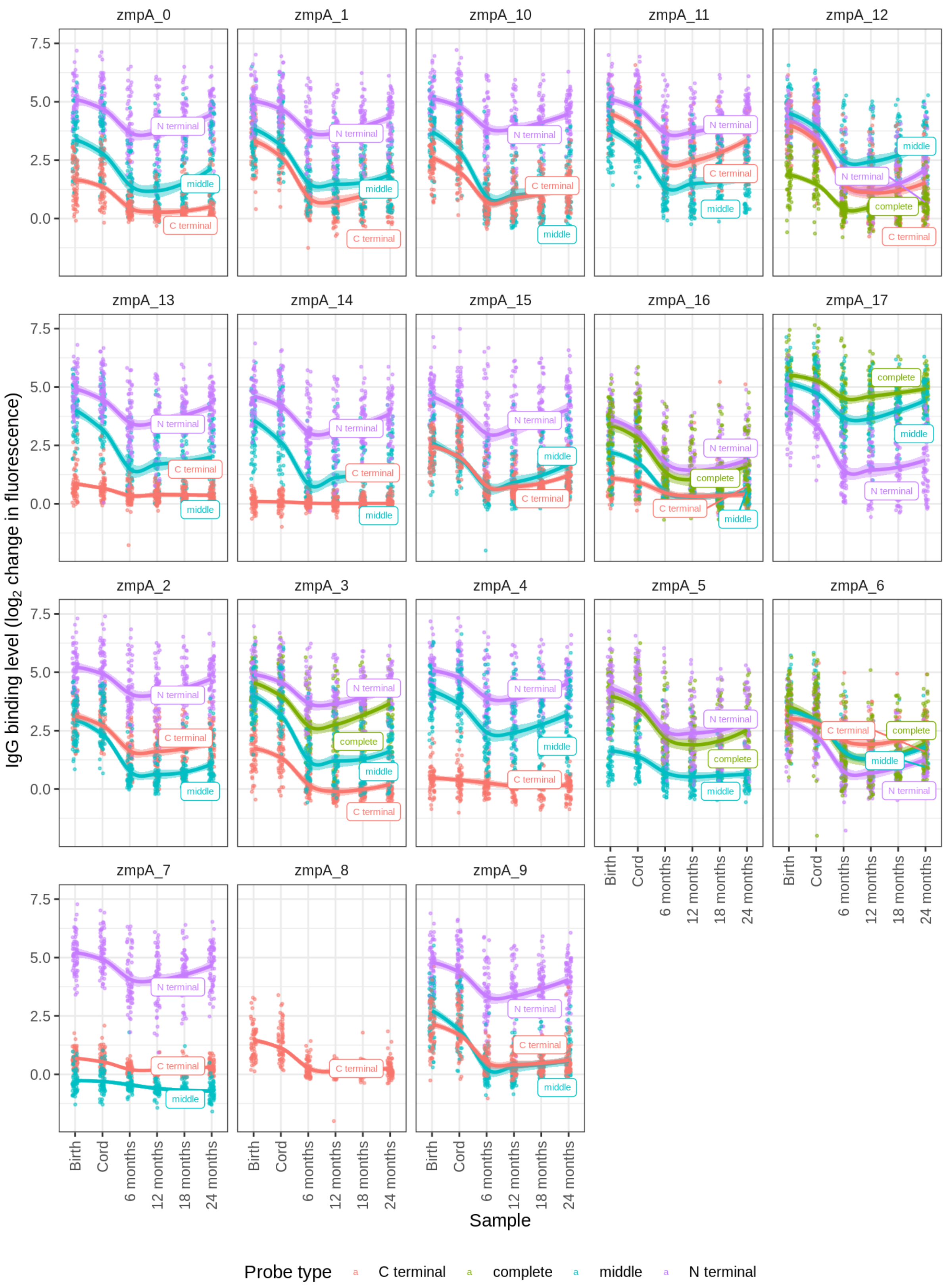

Supplementary Figure 25 Scatterplot detailing the binding of IgG to ZmpA probes. Data are show as for ZmpB probes in Supplementary Fig. 23. Despite the structural similarities between the ZmpA and ZmpB N termini, there is a considerably stronger post-birth decline in IgG binding to the ZmpA N terminus.

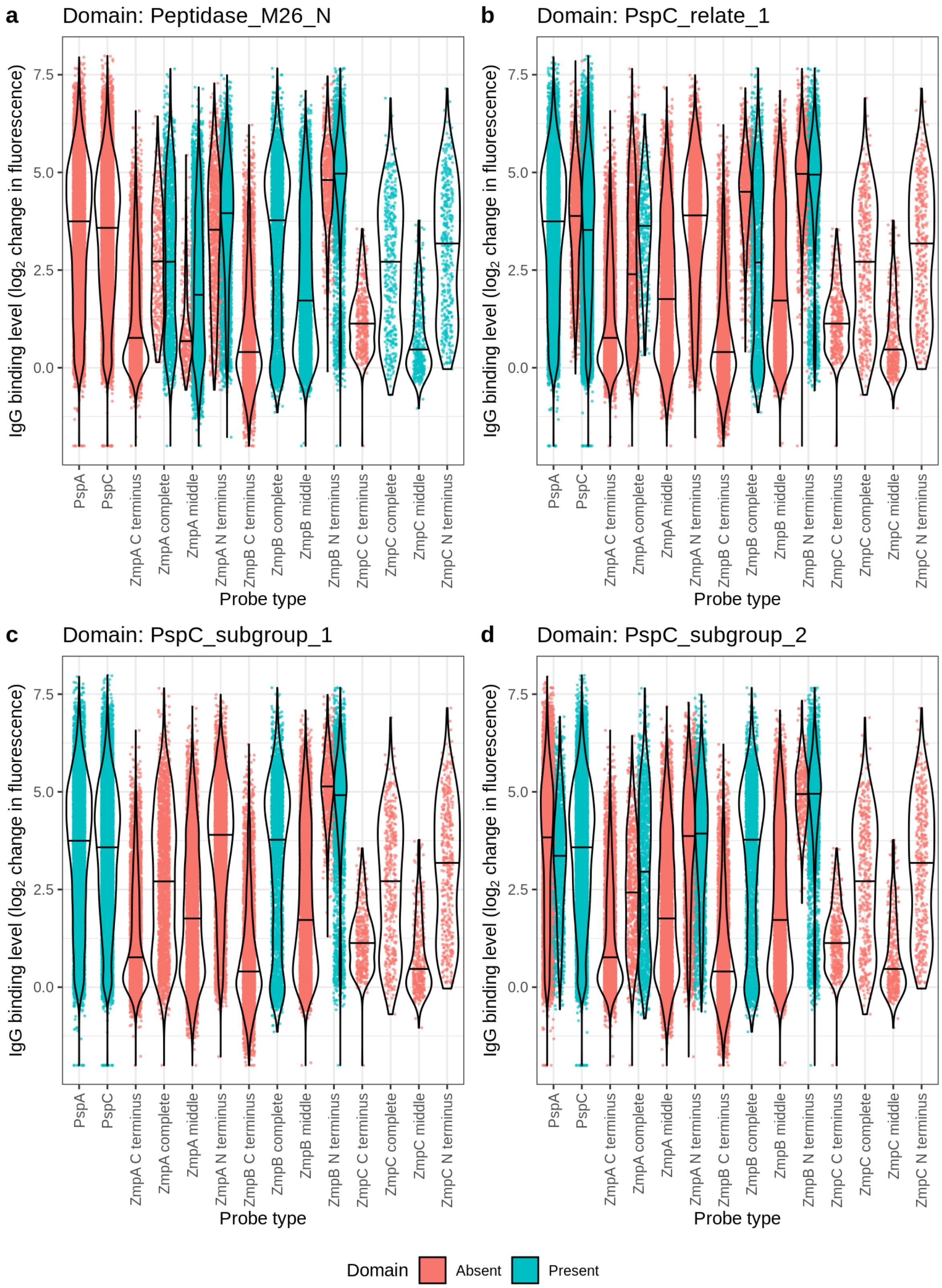

Supplementary Figure 26 Scatterplot detailing the binding of IgG to DCL protein probes. Each point represents the IgG binding to a particular probe across all datasets. Each panel separates probes by whether they contain the domain specified by the graph's title: (a) Peptidase_M26_N; (b) PspC_relate_1; (c) PspC_subgroup_1, and (d) PspC_subgroup_2. The violin plots summarise each distribution, with a horizontal line at the median value.

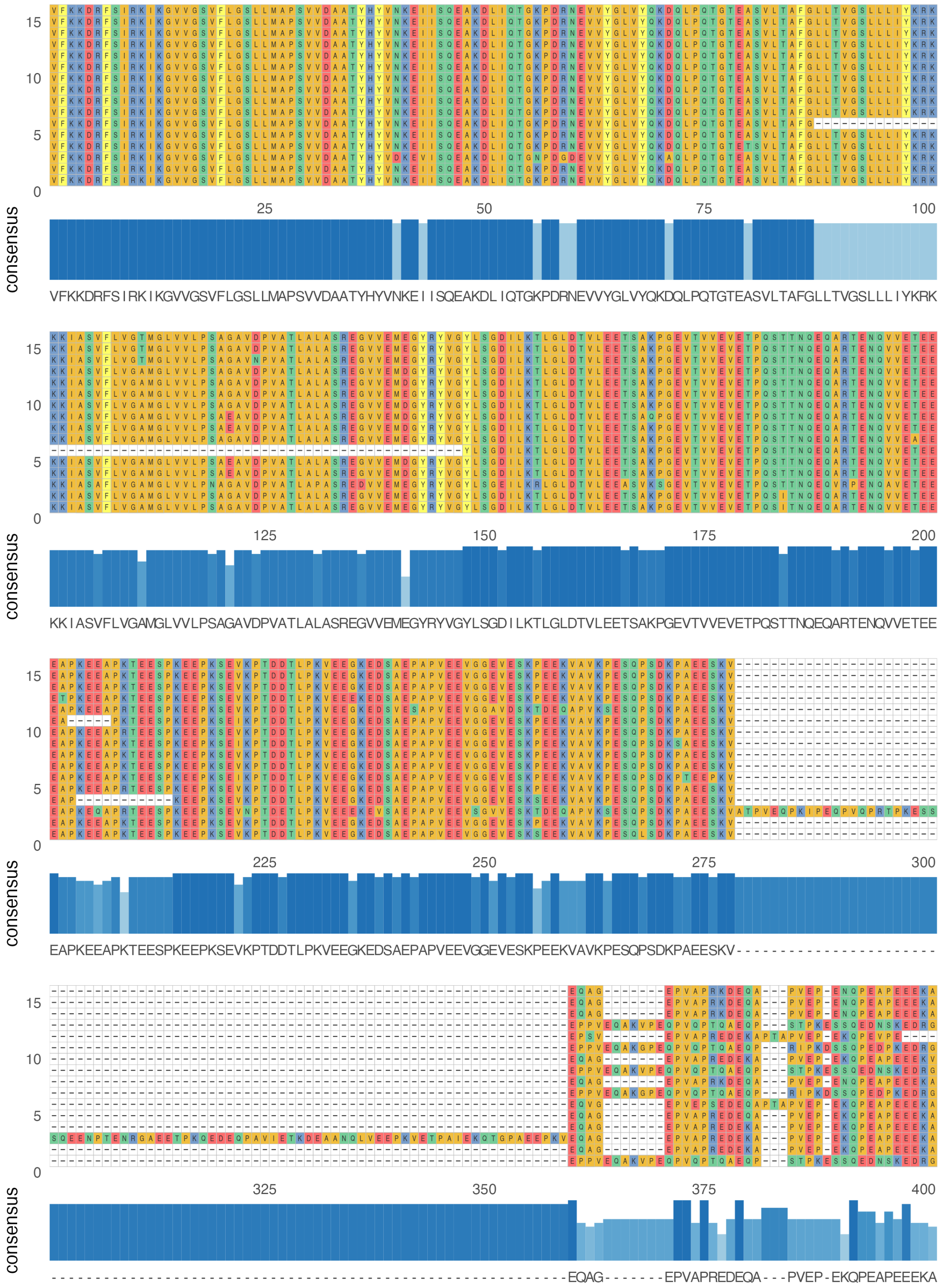

Supplementary Figure 27 Multiple sequence alignment of the N terminal region of ZmpB. The 16 variants of ZmpB that were used to represent the species-wide diversity of the protein on the array were aligned with Muscle using default settings. The blue bars under each site show the frequency of the corresponding major allele. This alignment shows the highly conserved N terminal 279 amino acids, which includes an LPQTG sortase attachment motif around position 75.

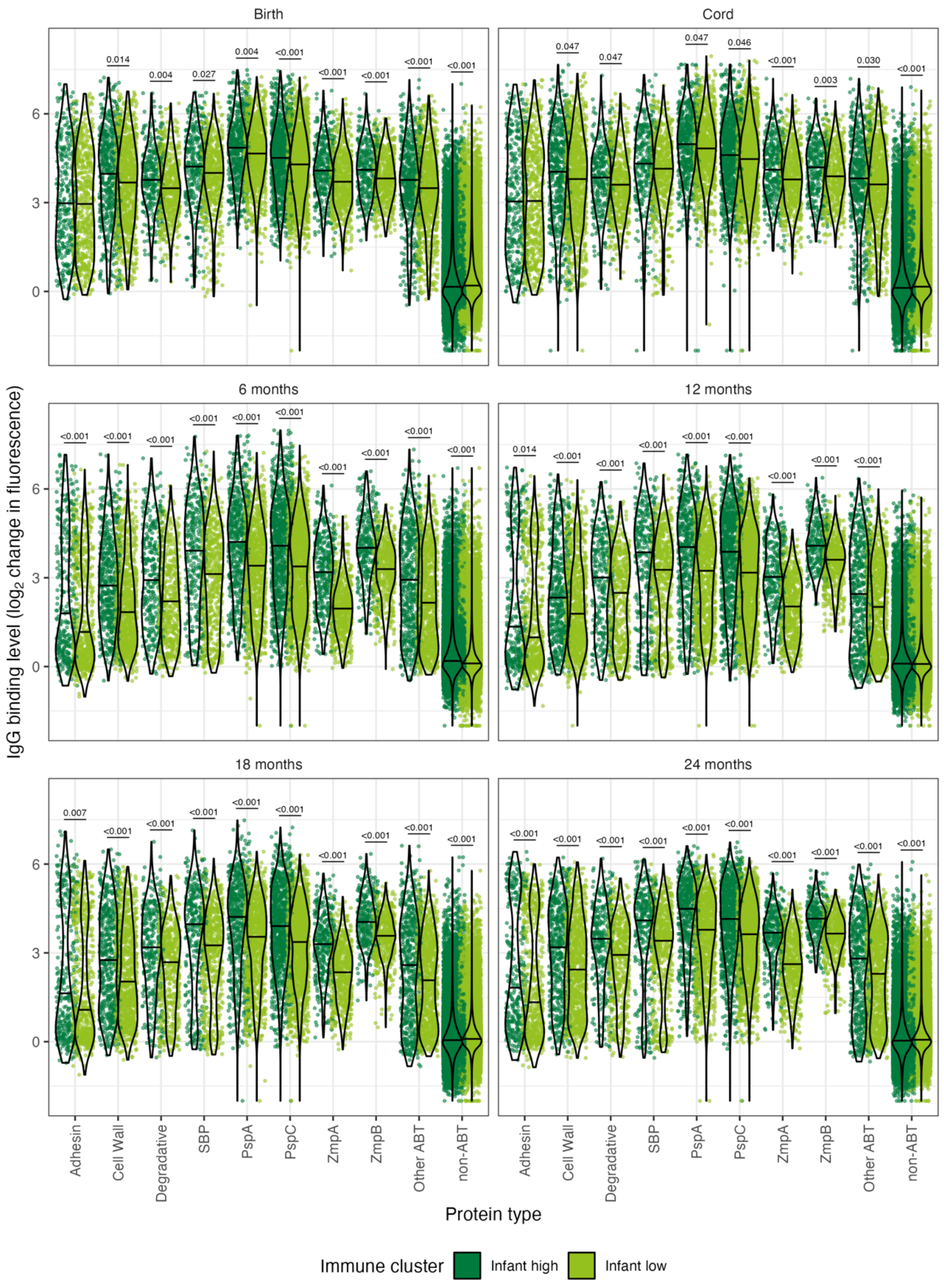

Supplementary Figure 28 Violin plots showing IgG responses to proteins separated by type, and the cluster to which individuals were assigned at 24 mo. Each panel shows data from a different sampling age. Each point represents the IgG binding level to a protein in a single individual. The significance of differences between clusters was assessed using two-tailed Wilcoxon rank sum tests. The significance of differences are annotated above the horizontal bars as *p* values. This comparison demonstrates the children with higher IgG levels at 24 mo also had significantly higher IgG levels when younger.

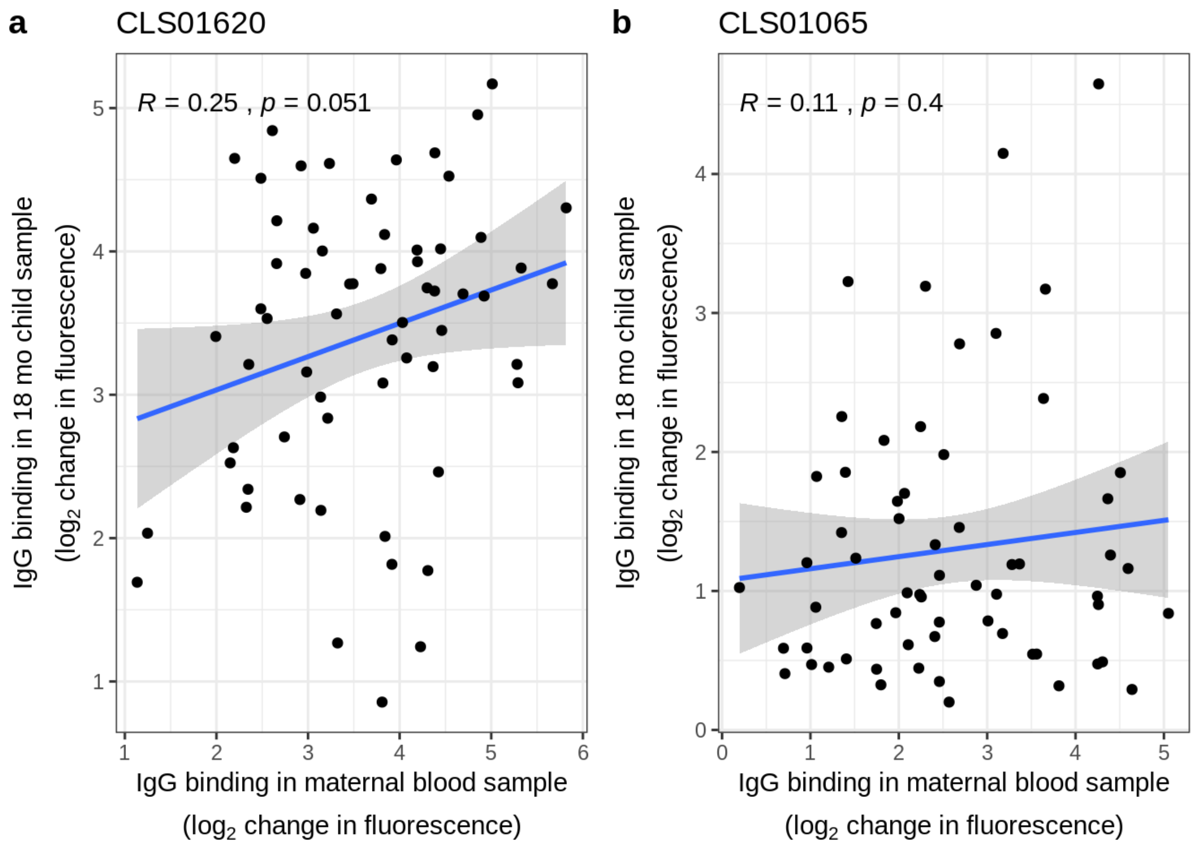

Supplementary Figure 29 Correlation between IgG levels levels in the maternal birth and 18 mo child samples. These proteins were selected as examples to visualise the relationships associated with (a) *R* close to 0.25, and (b) *R* close to 0.1. These demonstrate that the correlations at the level of individual proteins are weak. Nevertheless, the positive relationship observed across multiple proteins is significant, suggesting maternal immunity is positively associated with the formation of infant immunity.

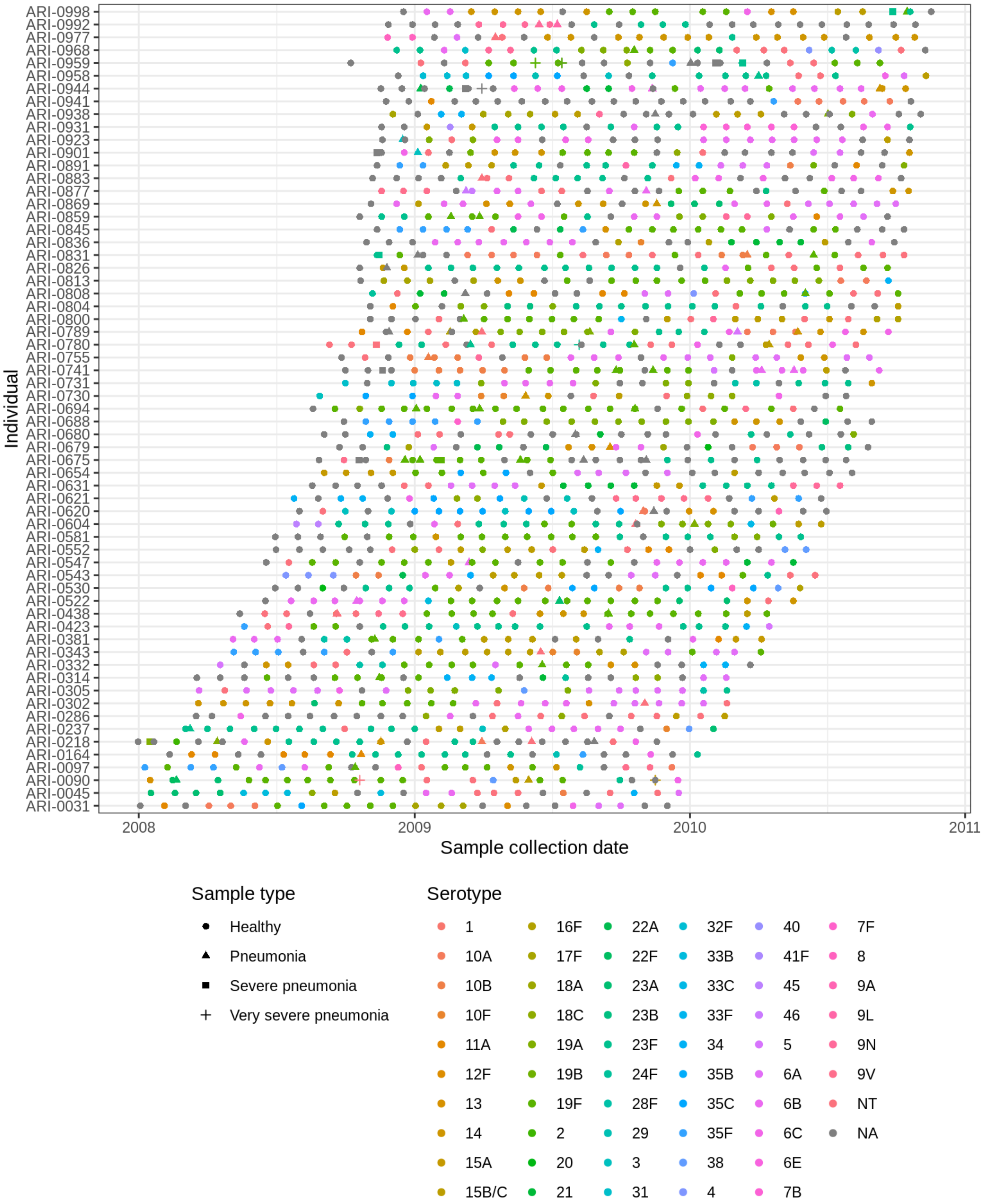

Supplementary Figure 30 Scatterplot showing the sampling of pneumococci from the studied cohort. Each point represents the detection of a pneumococcal sample, arranged by individual on the vertical axis, and date on the horizontal axis. The colour of the point represents the serotype. The shape of the point shows whether the sample was collected as part of routine monthly sampling, or was triggered by the clinical diagnosis of pneumonia.

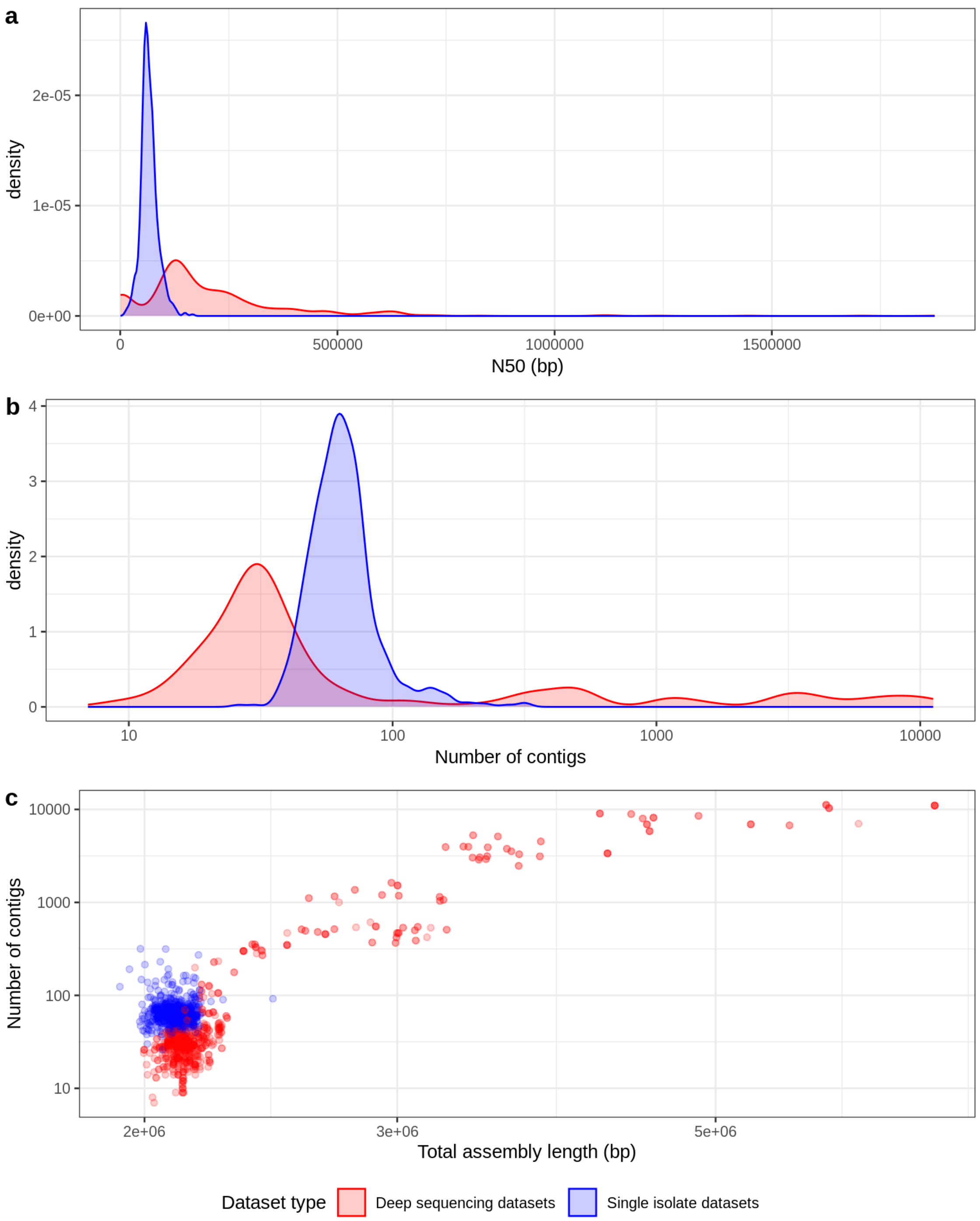

Supplementary Figure 31 Properties of genomic datasets. Each panel compares the statistics of draft assemblies generated from single genome and deep sequencing datasets. Deep sequencing assemblies were expected to be of more variable quality, as they were sequenced with longer sequence read lengths, but may represent a mixture of multiple diverse genotypes. (a) Density plot showing the N50 of the draft genome assemblies. (b) Density plot of the number of contigs in each assembly. (c) Scatterplot comparing the number of contigs in an assembly with its total length. All single isolate assemblies are close to the 2 Mb length expected of a pneumococcal genome. Many deep sequencing genomes have similar properties, suggesting they also correspond to a single isolate. However, many are longer, and fragmented into many contigs, suggesting these assemblies represent multiple genotypes.

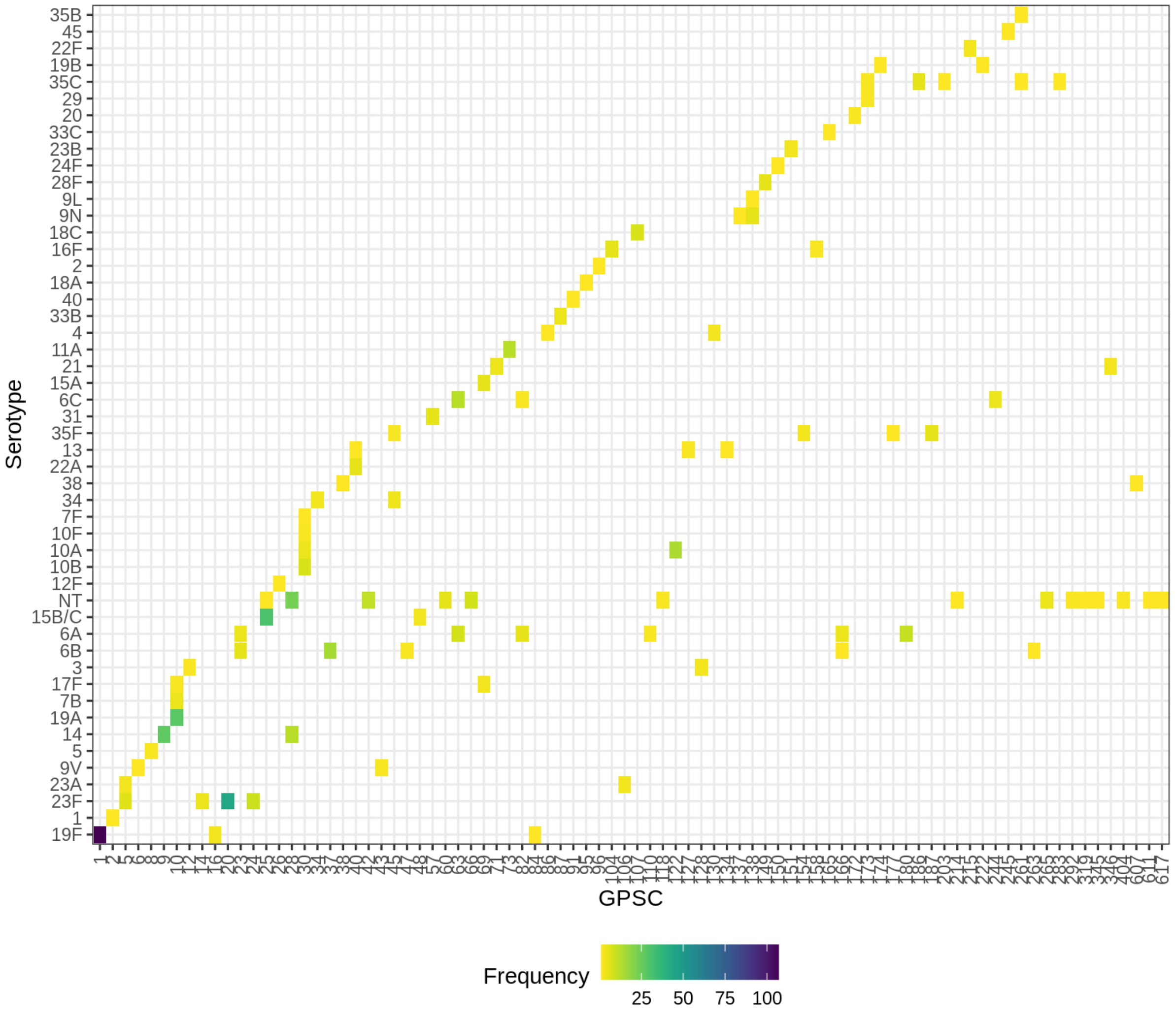

Supplementary Figure 32 Heatmap showing the distribution of serotypes across the Maela pneumococcal population. Each row of the grid corresponds to a different serotype. Each column of the grid corresponds to a different Global Pneumococcal Sequence Cluster (GPSC). The colour of the cell indicates the number of single isolate genomes assigned to the corresponding GPSC, expressing the corresponding serotype. The columns are arranged by the numeric order of the GPSCs, representing their frequency in the global pneumococcal population. The rows are arranged in order of the numerically lowest GPSC in which the serotype was detected, then by descending frequency within that GPSC.

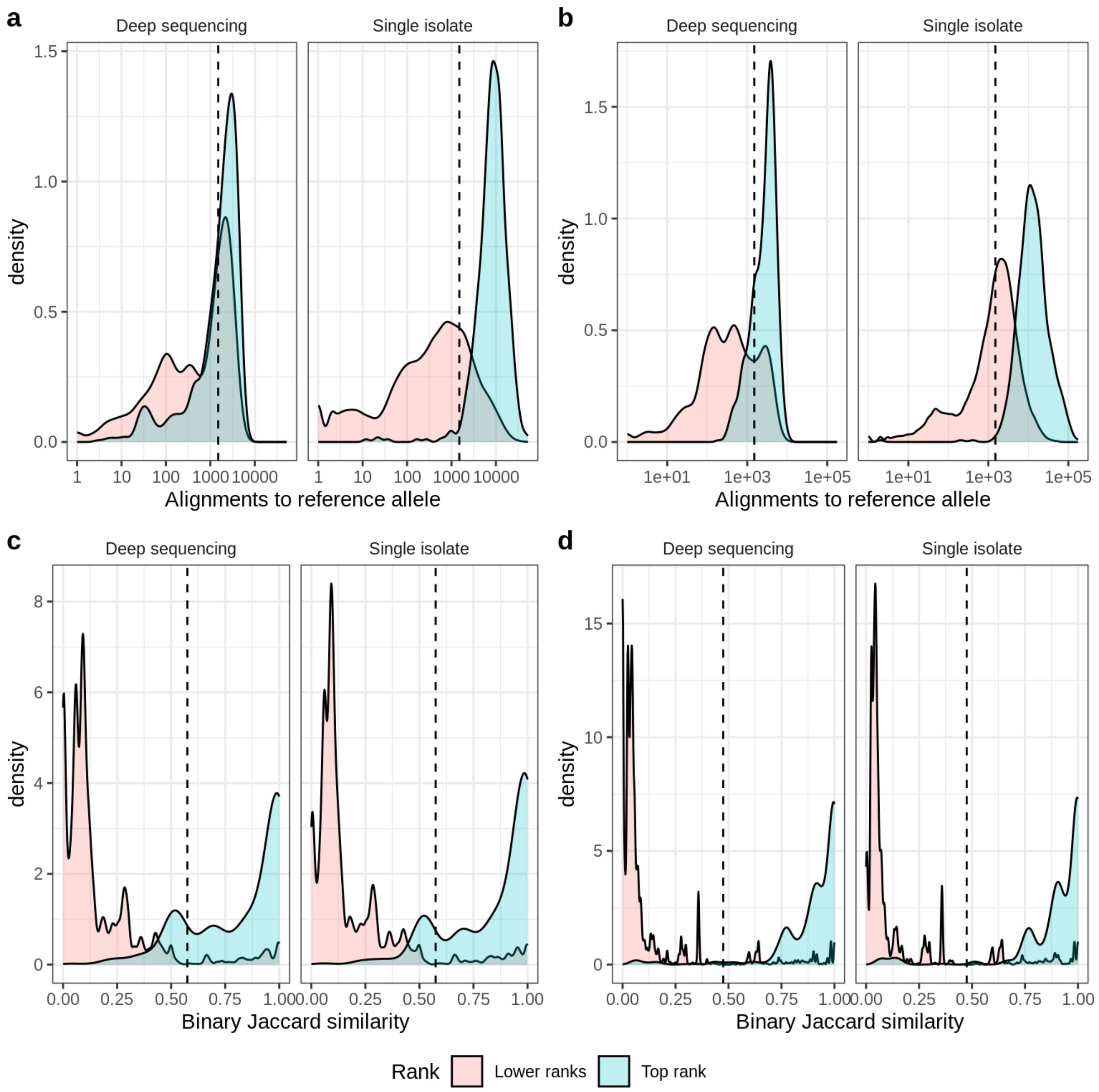

Supplementary Figure 33 Density plots enabling the identification of thresholds for the assignment of DCL variants to those represented on the array. (a) Density plot showing the number of reads aligned to each PspA variant across all isolates. The results are split by whether the read alignment counts corresponded to the maximum value for an isolate. These variants were considered top ranked. As each isolate was expected to encode one variant of PspA, but some isolates may not closely match all the variants on the array, the vertical dashed line represents a threshold that separated the stronger top-ranking hits from weaker top-ranking hits and lower-ranking hits. Therefore, for a PspA variant to be assigned to an isolate, it had to be the isolate's top-ranking hit, and the count of reads aligning to the variant had to surpass the defined threshold. (b) Density plot showing the number of reads aligned to each PspC variant across all isolates. Data are shown as in panel A. (c) Density plot showing the binary Jaccard similarities between the ZmpA sequences identified in genomic datasets and the ZmpA variants on the array, calculated using 15 aa-long *k*-mers. The results are split by whether the *k*-mer similarities corresponded to the maximum value for an isolate. These variants were considered top ranked. As each isolate

was expected to encode one variant of ZmpA, but some isolates may not closely match all the variants on the array, the vertical dashed line represents a threshold that separated the stronger top-ranking hits from weaker top-ranking hits and lower-ranking hits. Therefore, for a ZmpA variant to be assigned to an isolate, it had to be the isolate's top-ranking hit, and the *k*-mer similarity to the variant had to surpass the defined threshold. (d) Density plot showing the binary Jaccard similarities between the ZmpB sequences identified in genomic datasets and the ZmpB variants on the array. Data are displayed as in panel c.

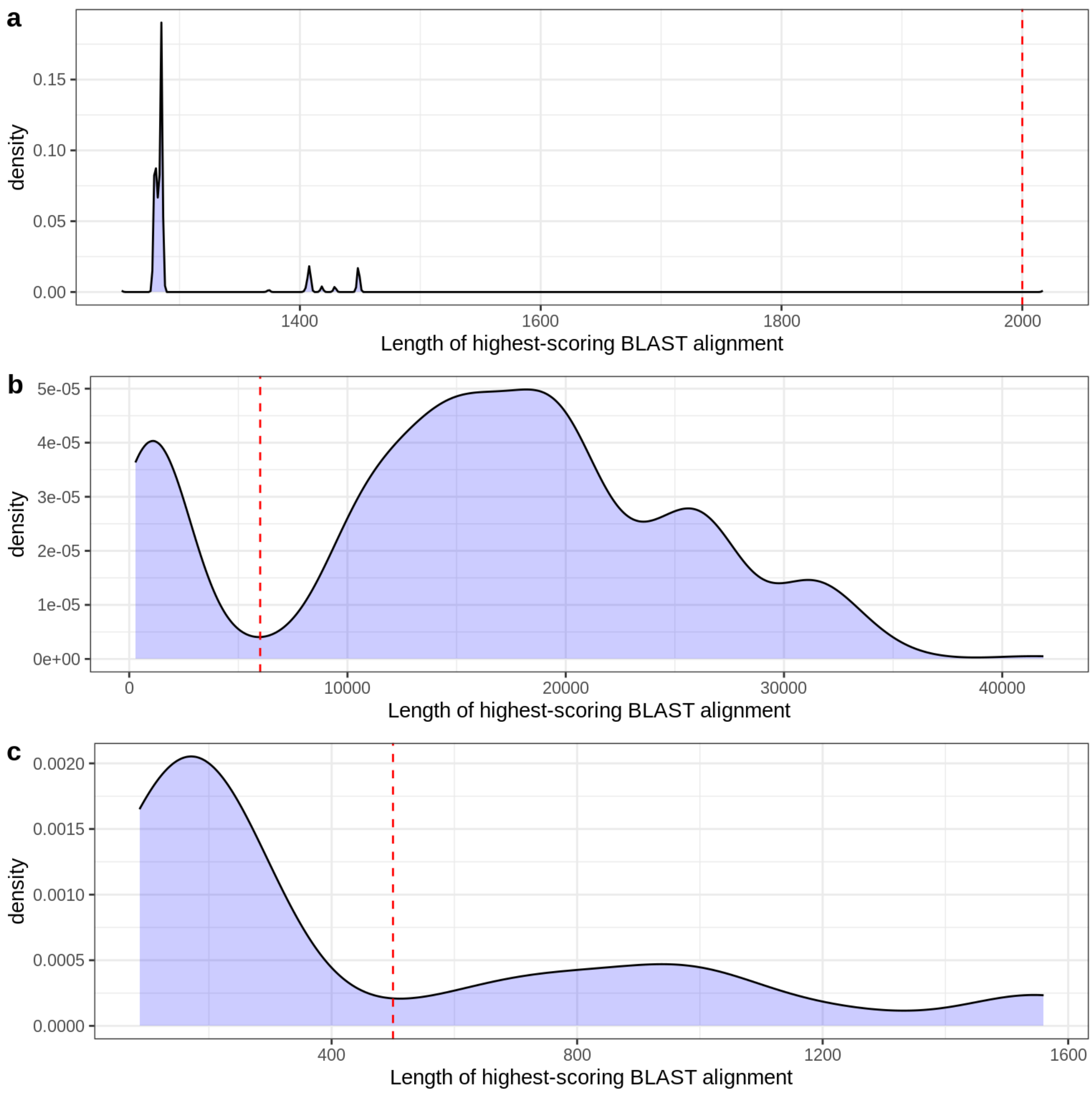

Supplementary Figure 34 Density plots justifying the thresholds used for quantifying the presence of proteins on the array that could not be identified through the clustering of protein sequences. Each panel shows the lengths of the highest-scoring BLAST alignment of a specified query to each genome assembly in the dataset. (a) Lengths of TBLASTN alignments of the ZmpE protein sequences against the draft genome assemblies. Only one isolate encoded the full-length protein. (b) Lengths of BLASTN alignments of a database of pneumococcal prophage sequences against the draft genome assemblies. Full-length prophages are typically greater than 20 or 30 kb in length, but their highly variable sequences are often fragmented in draft assemblies, preventing BLASTN from identifying intact elements. (c) Lengths of TBLASTN alignments of the PblB phage protein sequence against the draft genome assemblies.

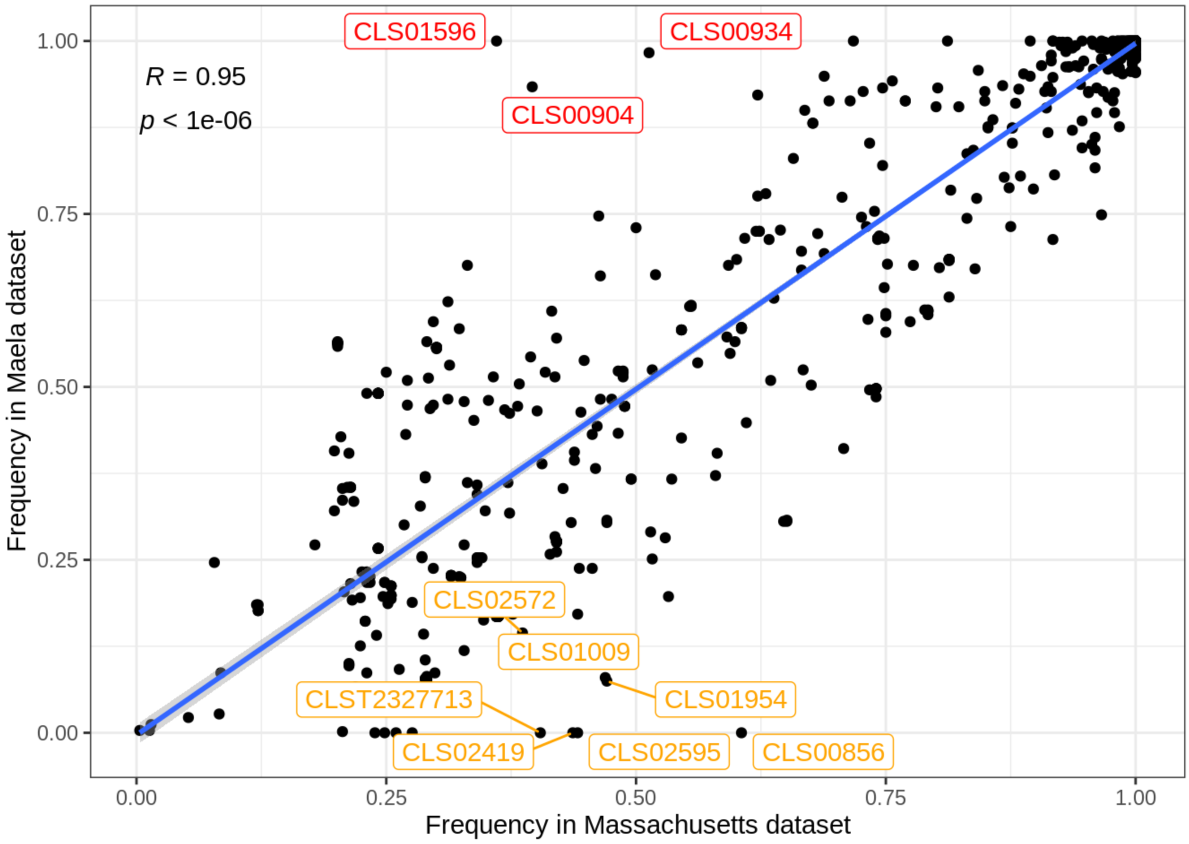

Supplementary Figure 35 Scatterplot comparing the frequency of clusters of orthologous genes (COGs) in the original Massachusetts population in which they were inferred, and the single isolate genomes used in this study. The blue line shows the best-fitting relationship between the frequencies. The associated grey shading shows the 95% confidence interval of this relationship. This linear relationship is very close to the line of identity, confirming that the COGs were identified at similar overall frequencies in this cohort as in previous analyses of pneumococcal populations. The points labelled in red correspond to COGs found at higher than expected frequencies in the Maela dataset. The points labelled in yellow correspond to COGs found at lower than expected frequencies in the Maela dataset. All of these outliers correspond to genes that are often fragmented by misassembly. Therefore the deviations in frequency likely represent differences in assembly quality between datasets sequenced at different times, using different platforms.

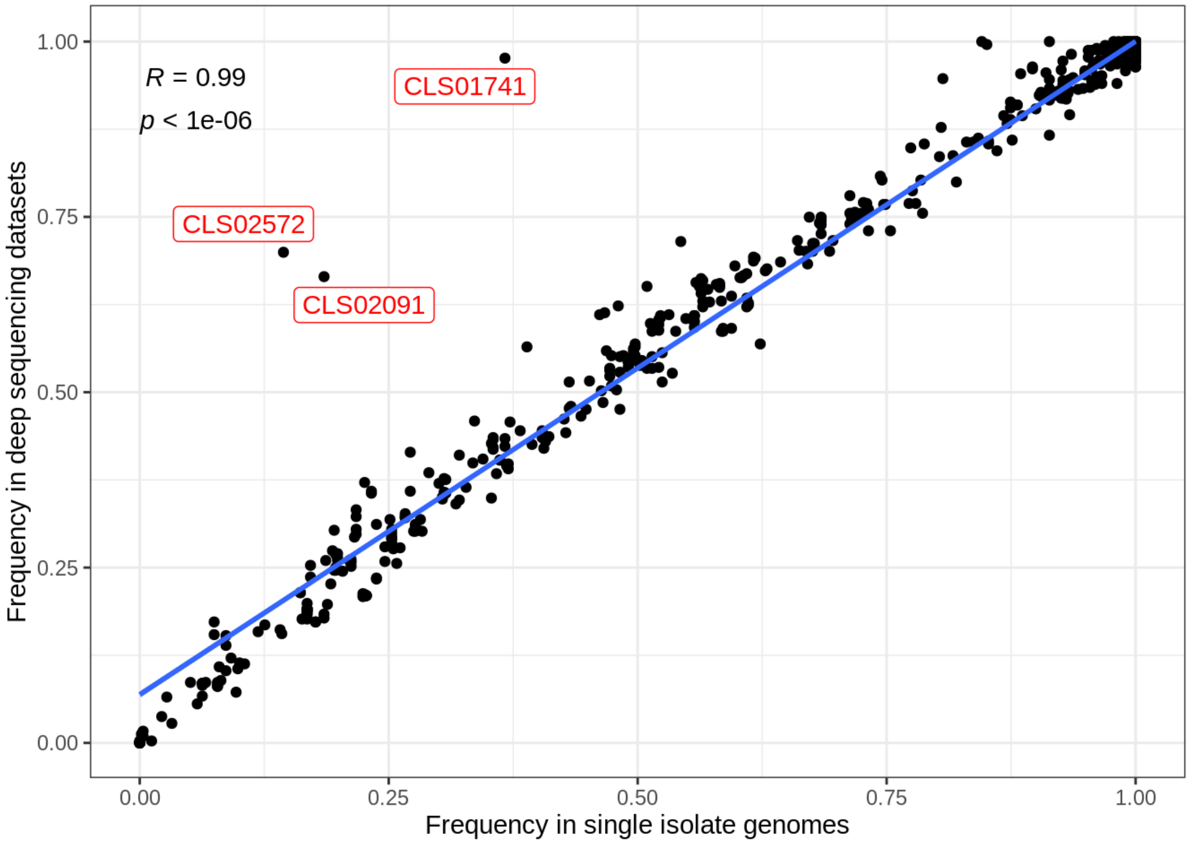

Supplementary Figure 36 Scatterplot comparing the frequency of clusters of orthologous genes (COGs) in the single isolate genomes and deep sequencing datasets used in this study. The blue line shows the best-fitting relationship between the frequencies. The associated grey shading shows the 95% confidence interval of this relationship. This linear relationship is very close to the line of identity, confirming that the joint inference of COGs across both types of dataset generates consistent results overall. The points labelled in red correspond to COGs found at higher than expected frequencies in the deep sequencing datasets. All of these outliers correspond to genes that are often fragmented by misassembly, such as when deep sequencing datasets contain multiple genotypes, and therefore the differences in frequency likely represent differences in assembly quality.

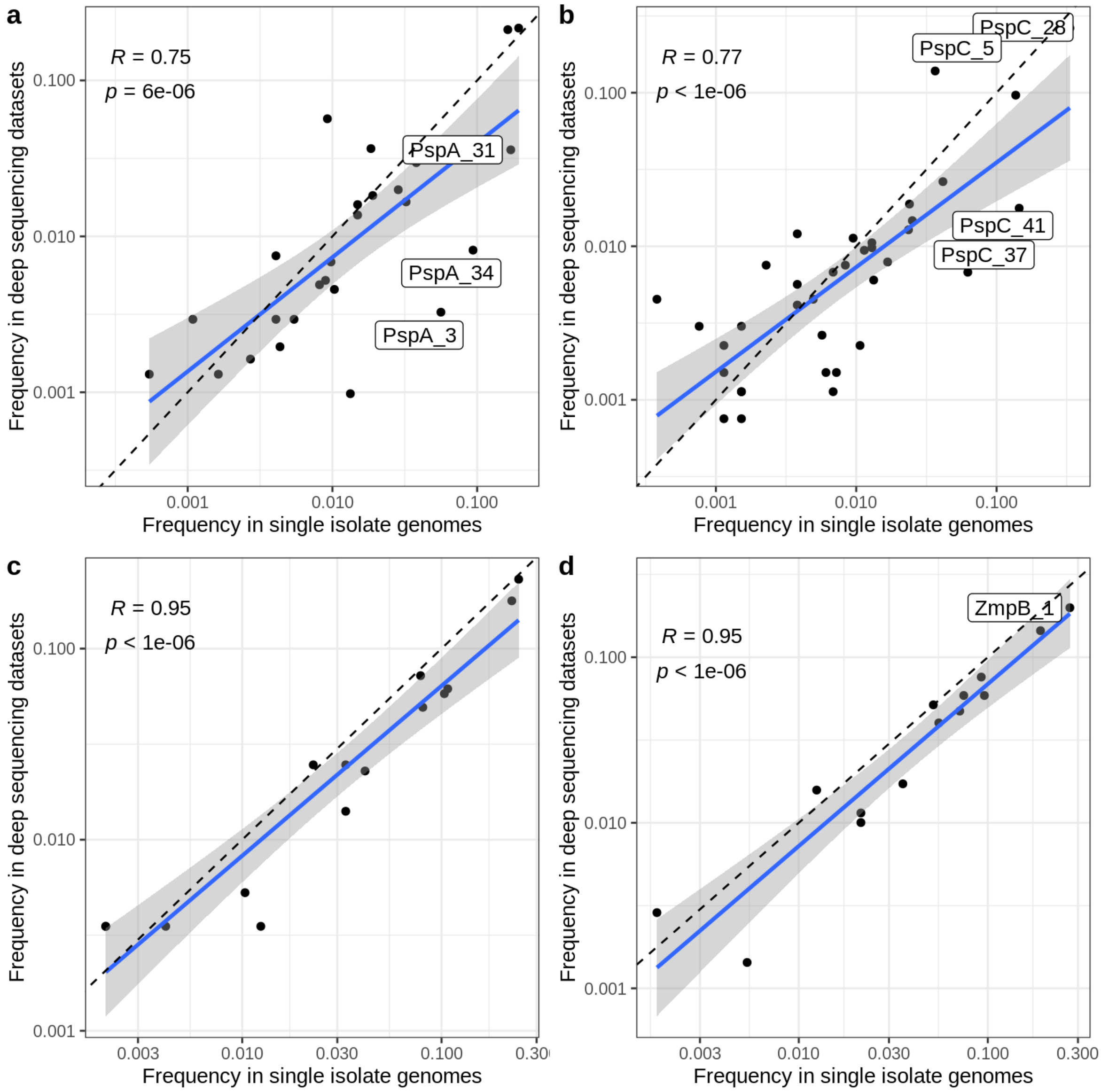

Supplementary Figure 37 Scatterplots comparing the frequency of DCL variants in the single isolate genomes and deep sequencing datasets used in this study. The black dashed line shows the line of identity. The blue lines show the best-fitting linear relationship between the frequencies. The associated grey shading shows the 95% confidence intervals of these relationships. For (a) PspA and (b) PspC, the frequencies of each variant are lower in the deep sequencing dataset than in the single isolate genomes. This is likely a consequence of the difficulty of inferring alleles from mixed samples using a mapping-based approach with the strict thresholds inferred from single isolate data (Supplementary Fig. 33). Nevertheless, the relative frequencies of PspA and PspC variants are similar across both types of sequencing data, implying there is not a systematic bias towards a subset of alleles that depends upon the method used to generate the data. For (c) ZmpA and (d) ZmpB, the linear relationships are very close to the line of identity, confirming that the inference of DCL variants across both types of dataset was similar in both relative and absolute frequencies. This is likely a consequence of inferring the presence of variants using thresholds that were relatively less stringent than those employed for PspA and PspC. These were enabled by the greater distinction between top ranking and lower ranking matches for ZmpA and ZmpB (Supplementary Fig. 33).

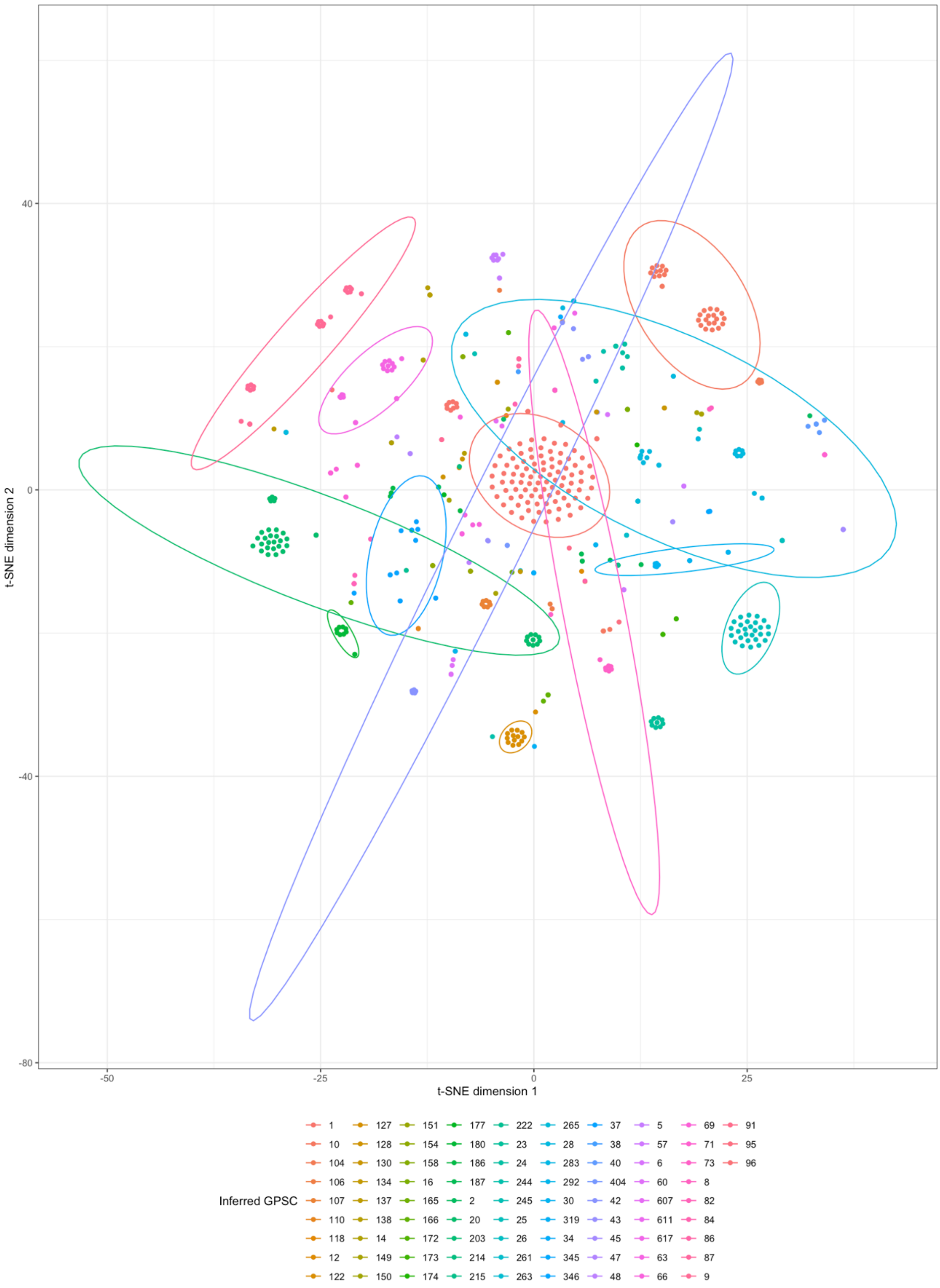

Supplementary Figure 38 Scatterplot showing a t-SNE projection of all single isolate genomes, based on the presence and absence matrix of clusters of orthologous genes. Points are coloured according to the GPSC to which the isolate was assigned. For all GPSCs represented by five or more isolates, all isolates belonging to the GPSC are grouped together by an ellipse. The co-clustering of isolates from the same GPSC is consistent with the observation that divergence in the core and accessory genomes is correlated. The projection was calculated with a maximum of 50,000 iterations.

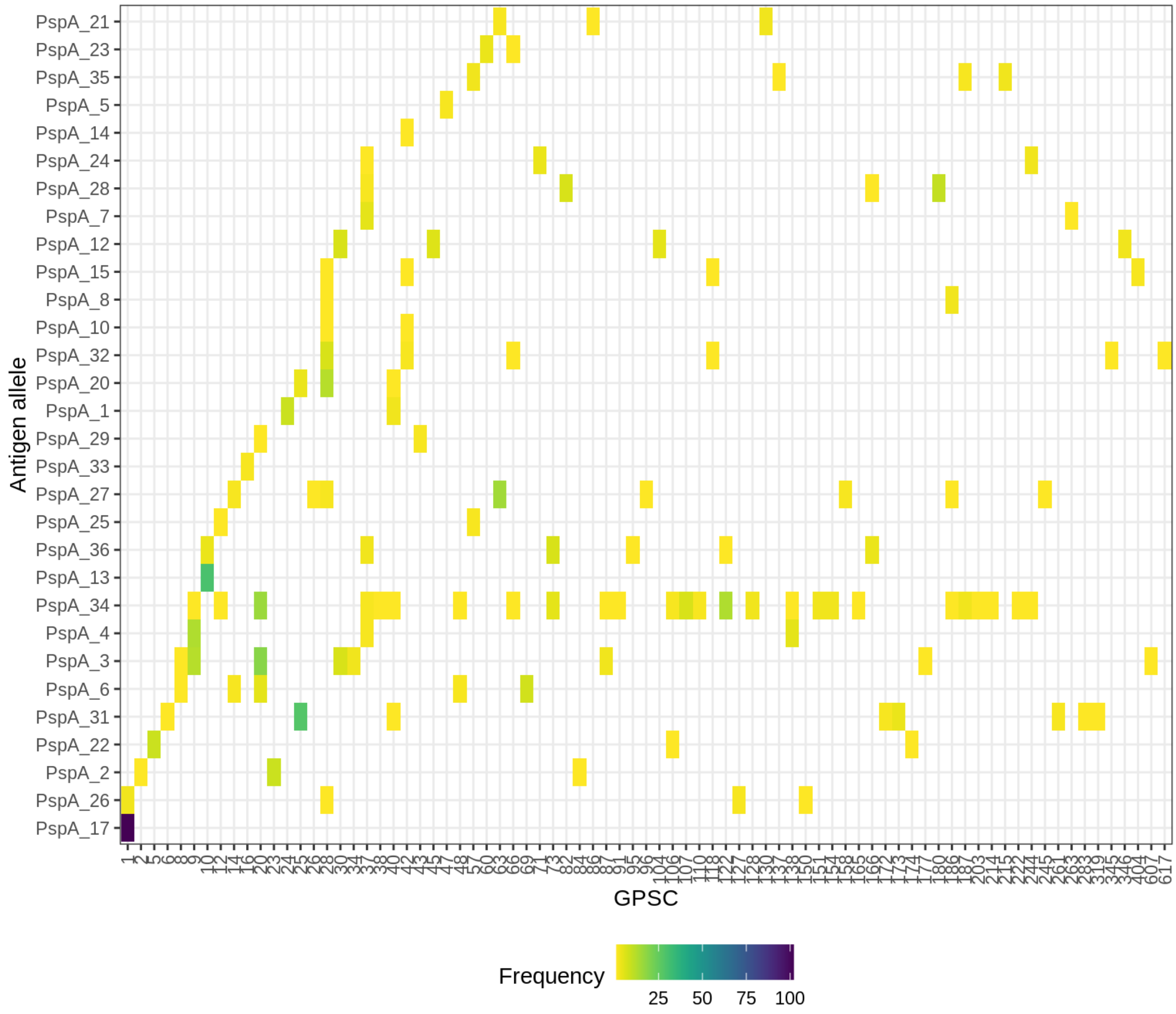

Supplementary Figure 39 Heatmap showing the distribution of PspA variants across the Maela pneumococcal population. Each row of the grid corresponds to a different PspA variant. Each column of the grid corresponds to a different GPSC. The colour of the cell indicates the number of single isolate genomes assigned to the corresponding GPSC, expressing the corresponding PspA variant. The columns are arranged by the numeric order of the GPSCs, representing their frequency in the global pneumococcal population. The rows are arranged in order of the numerically lowest GPSC in which the variant was detected, then by descending frequency within that GPSC. The strong association of many variants with one, or few, GPSCs results in many points lying along a “diagonal” line extending up the plot. This pattern has been previously observed in analyses of multi-strain pathogens. The correlation between antigen profiles and population structure is consistent with reproducible inference of the PspA variant on the array best representing that encoded by isolates’ genomes.

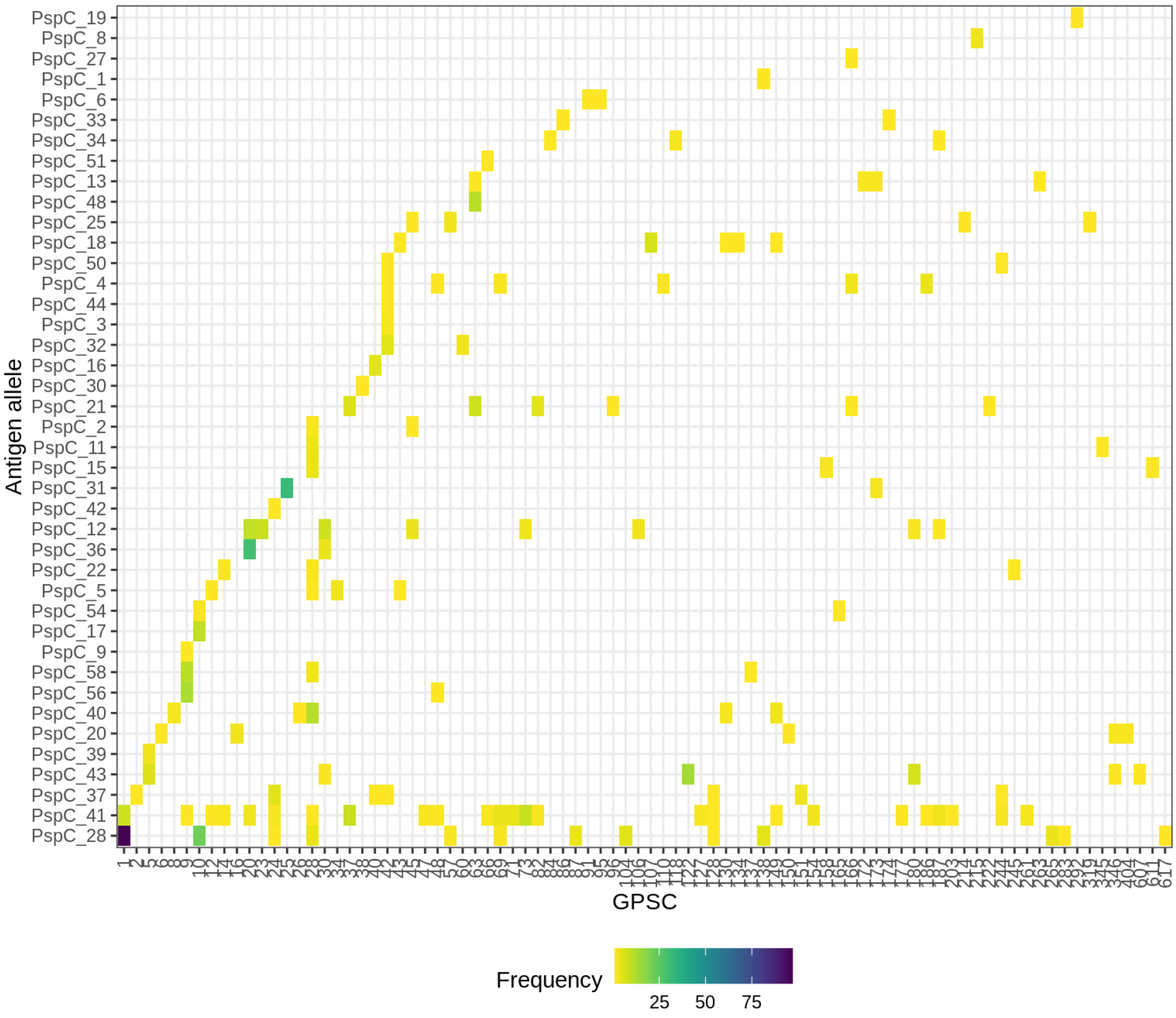

Supplementary Figure 40 Heatmap showing the distribution of PspC variants across the Maela pneumococcal population, displayed as described in Supplementary Fig. 39.

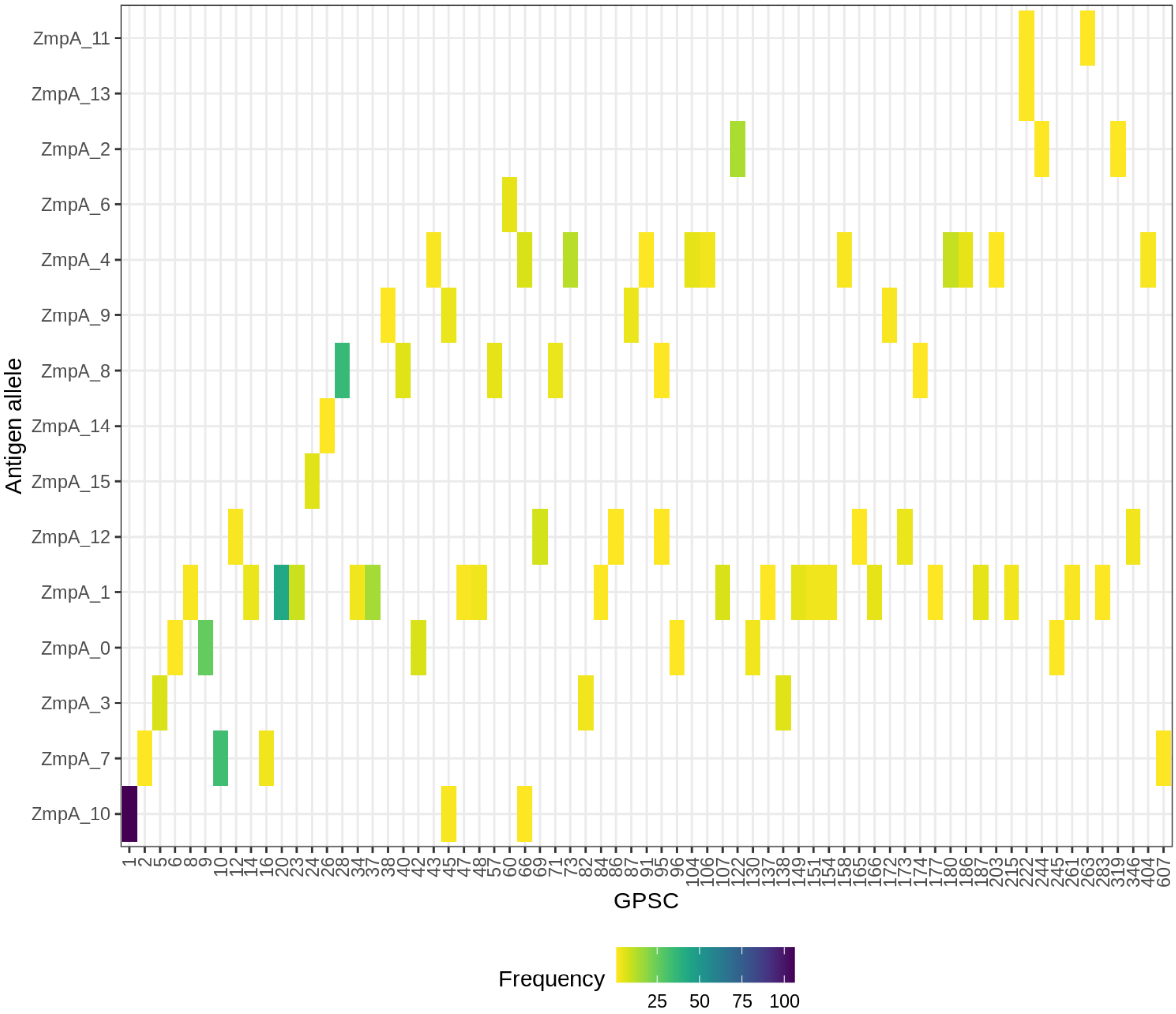

Supplementary Figure 41 Heatmap showing the distribution of ZmpA variants across the Maela pneumococcal population, displayed as described in Supplementary Fig. 39.

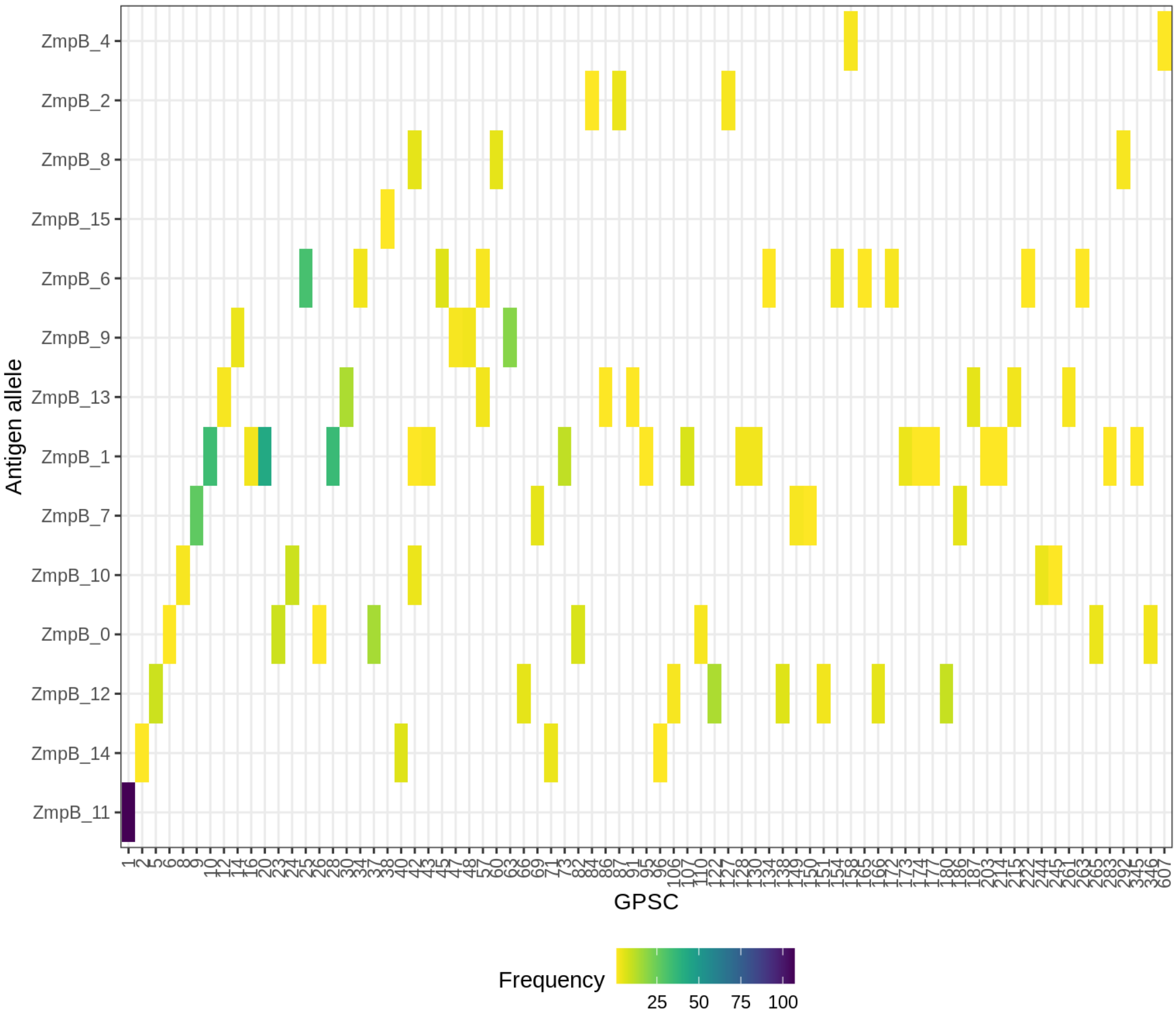

Supplementary Figure 42 Heatmap showing the distribution of ZmpB variants across the Maela pneumococcal population, displayed as described in Supplementary Fig. 39.

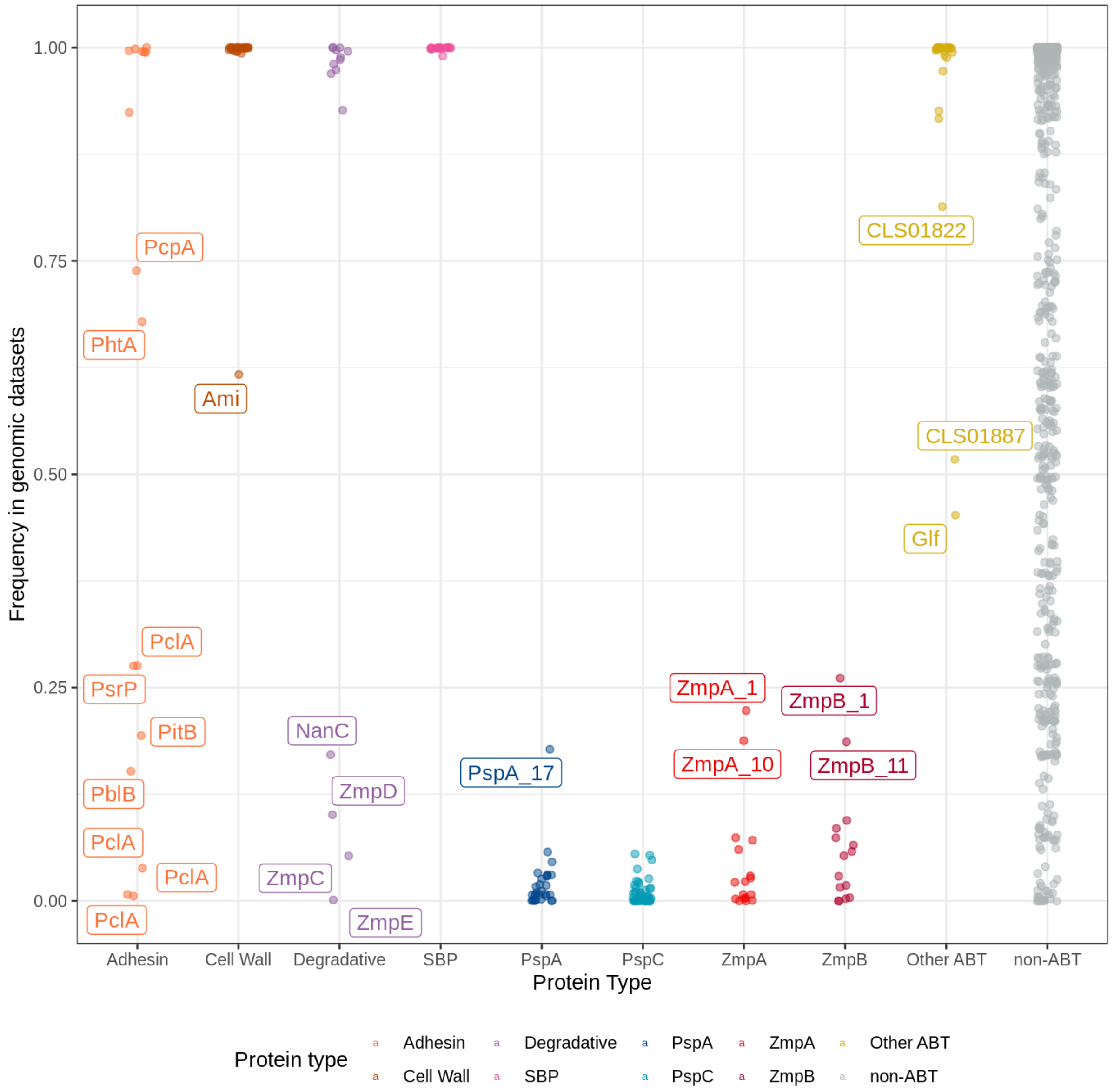

Supplementary Figure 43 Scatterplot showing the frequency of proteins represented on the panproteome array in the Maela pneumococcal population. The horizontal axis separates proteins by type. The vertical axis shows the frequency of the protein in the pneumococcal population, with a frequency of one indicating the protein is ubiquitous across all sampled isolates.

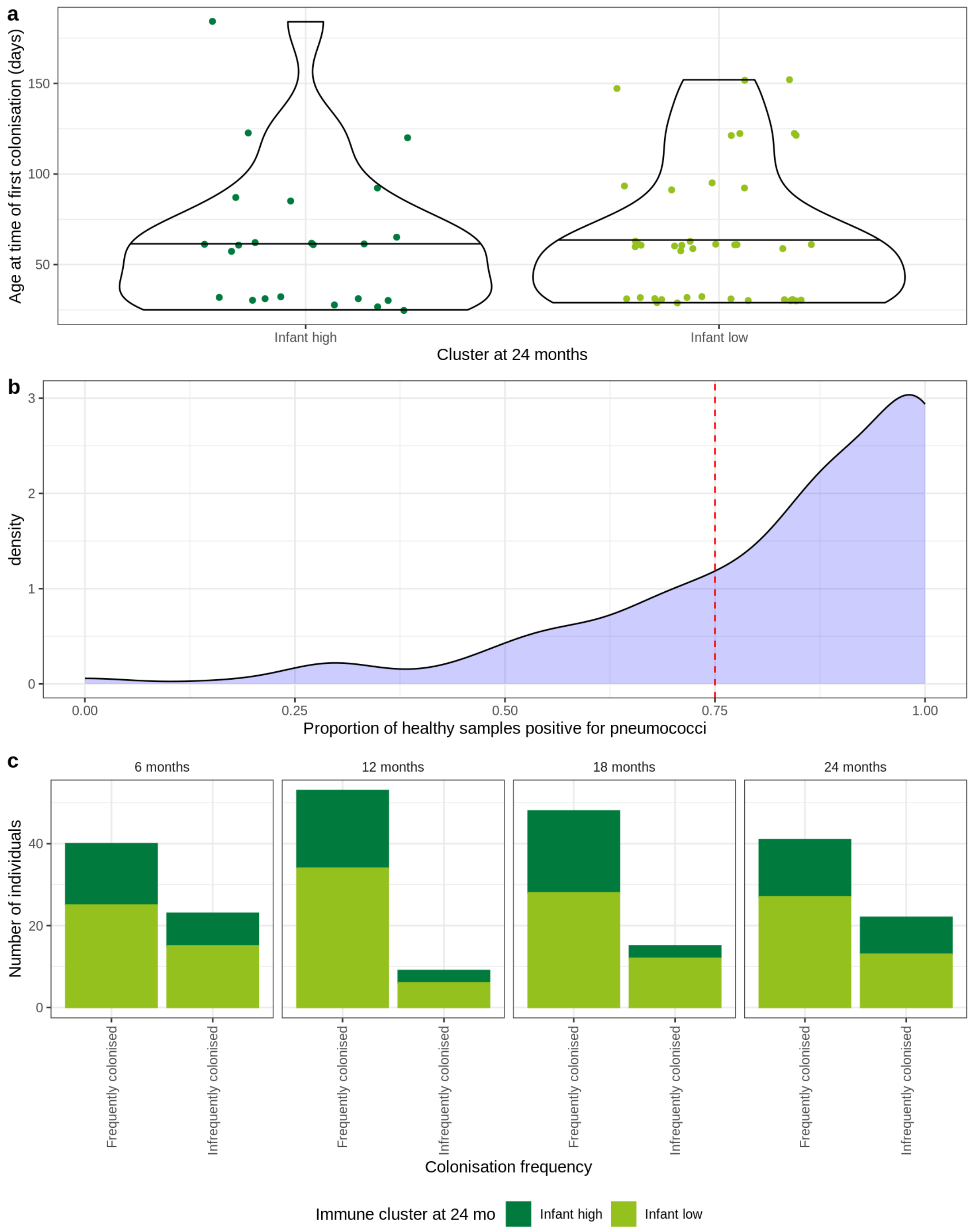

Supplementary Figure 44 Differences in the early colonisation of individuals. (a) Violin plot showing the age of each individual when they were first identified as being colonised with a pneumococcus. Individuals are categorised by the cluster to which they were assigned at 24 mo. Violin plots summarise the distribution, with a horizontal line at the median position. (b) Density plot showing the proportion of nasopharyngeal swabs from healthy carriage that were positive for pneumococci during the intervals between serological sampling for each individual. The presence of pneumococci was inferred by microbiological culturing. The vertical red dashed line shows the threshold that was used to identify periods of frequent pneumococcal carriage preceding a serological sample. (c) Bar plot showing the relationship between individuals' frequency of colonisation at different ages and their immune cluster assignment at 24 mo.

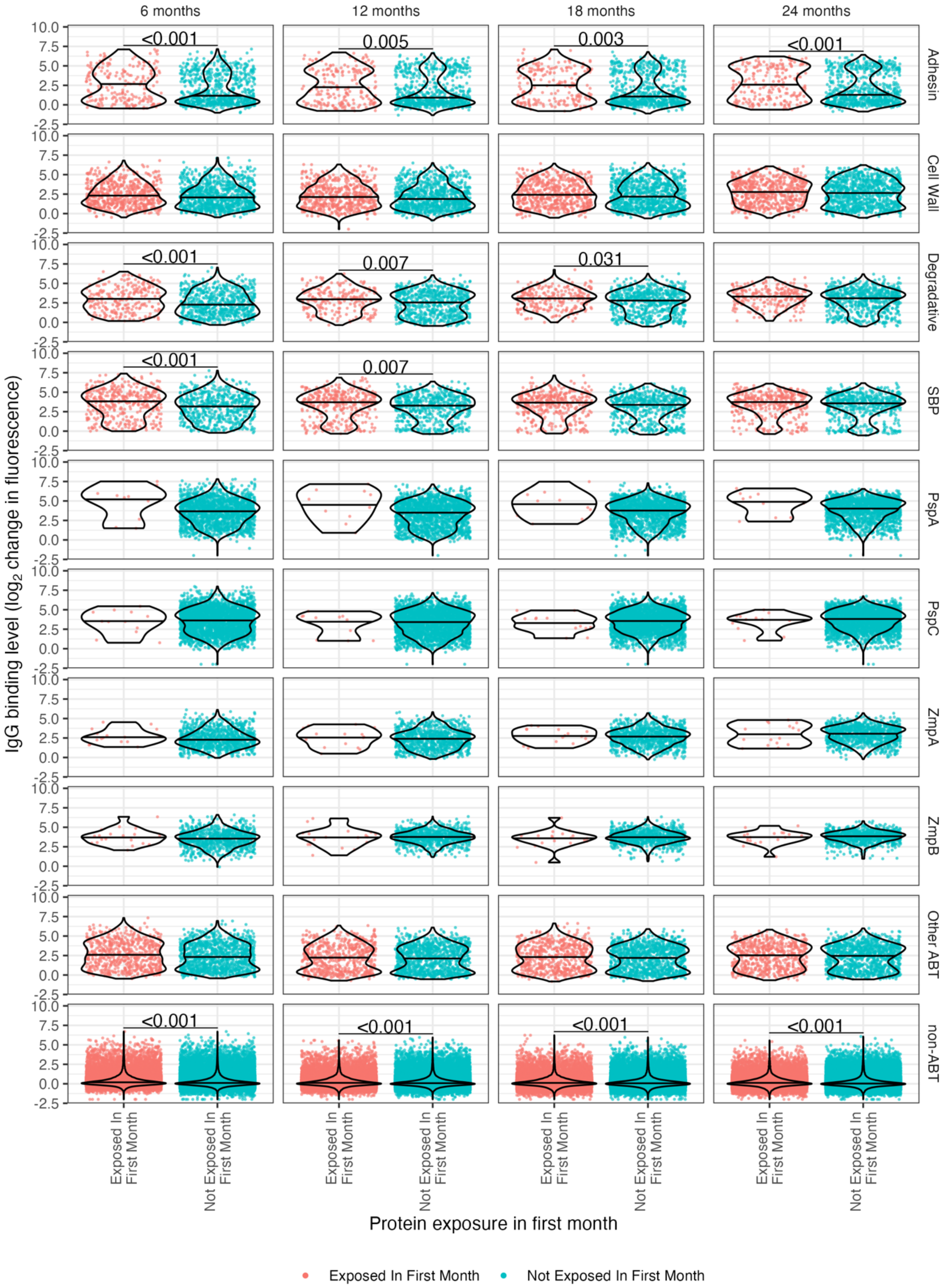

Supplementary Figure 45 Violin plots showing the differences in IgG binding to proteins to which individuals were inferred to have been exposed in the first month after their birth, relative to those proteins for which there was no evidence of exposure from the individual's colonisation history during this period. Data are presented as described for Fig. 5.

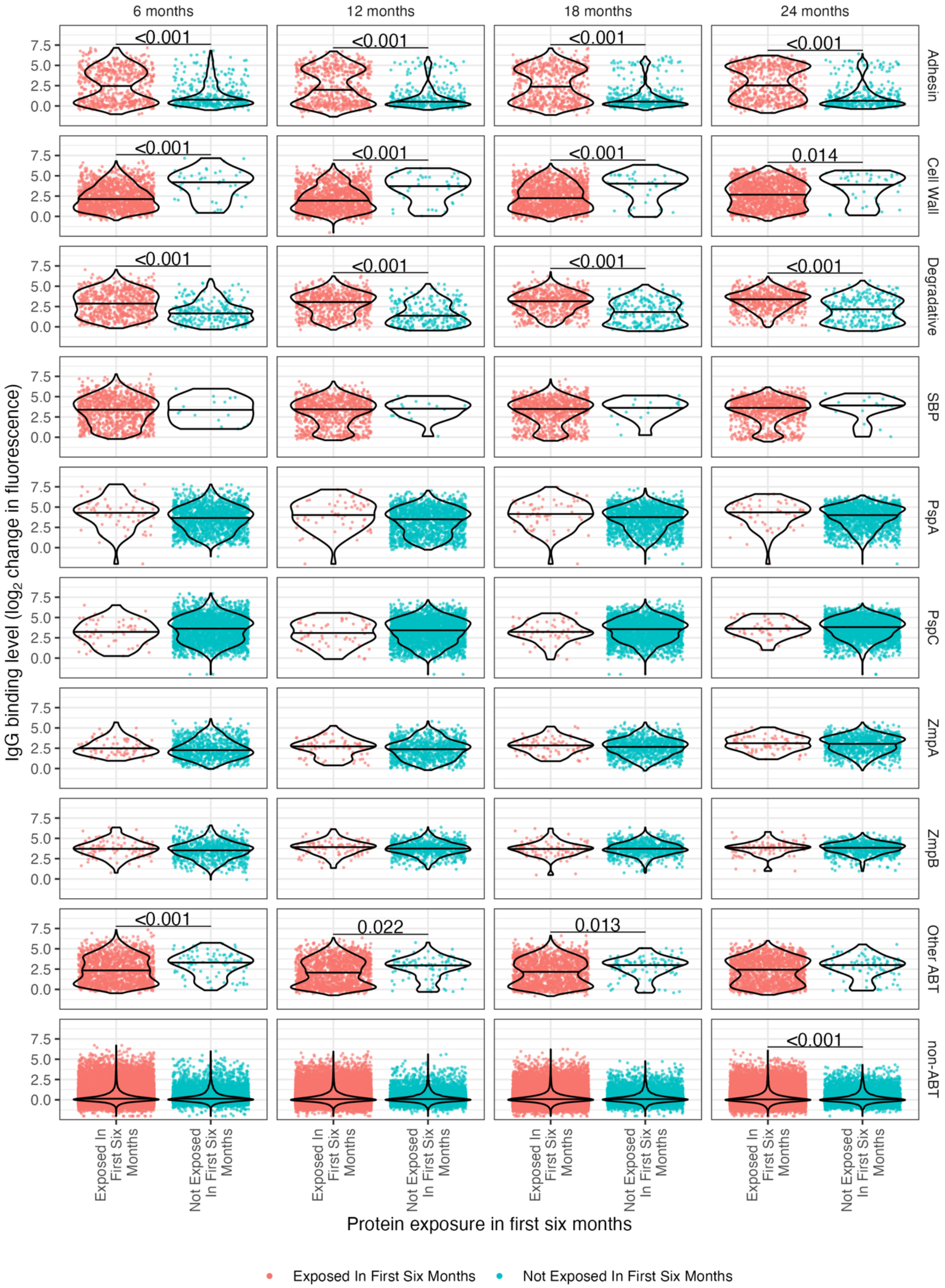

Supplementary Figure 46 Violin plots showing the differences in IgG binding to proteins to which individuals were inferred to have been exposed in the first six months after their birth, relative to those proteins for which there was no evidence of exposure from the individual's colonisation history in this period. Data are presented as described for Fig. 5.

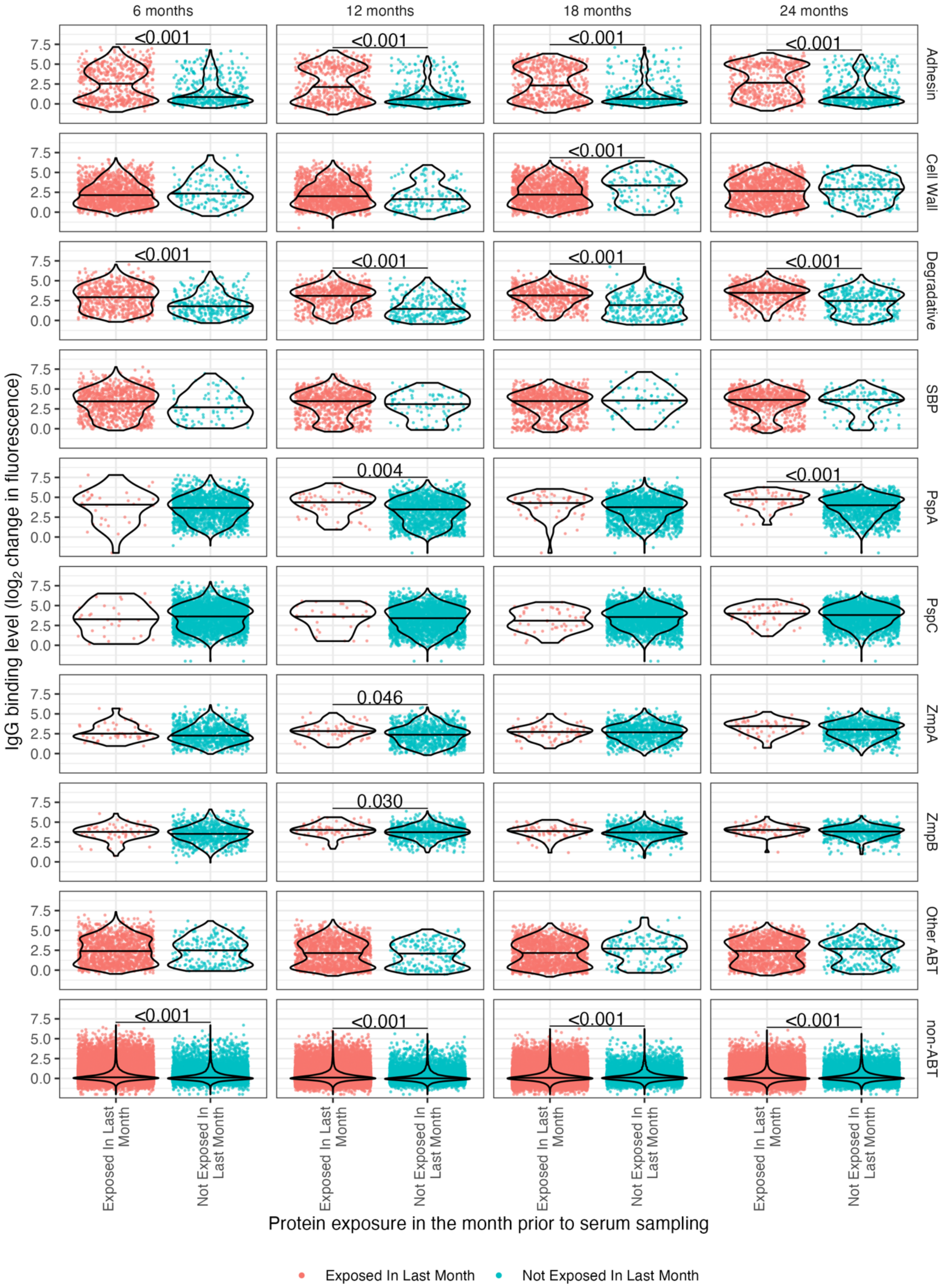

Supplementary Figure 47 Violin plots showing the differences in IgG binding to proteins to which individuals were inferred to have been exposed in the month prior to the serum sample being taken, relative to those proteins for which there was no evidence of exposure from the individual's colonisation history during this period. Data are presented as described for Fig. 5.

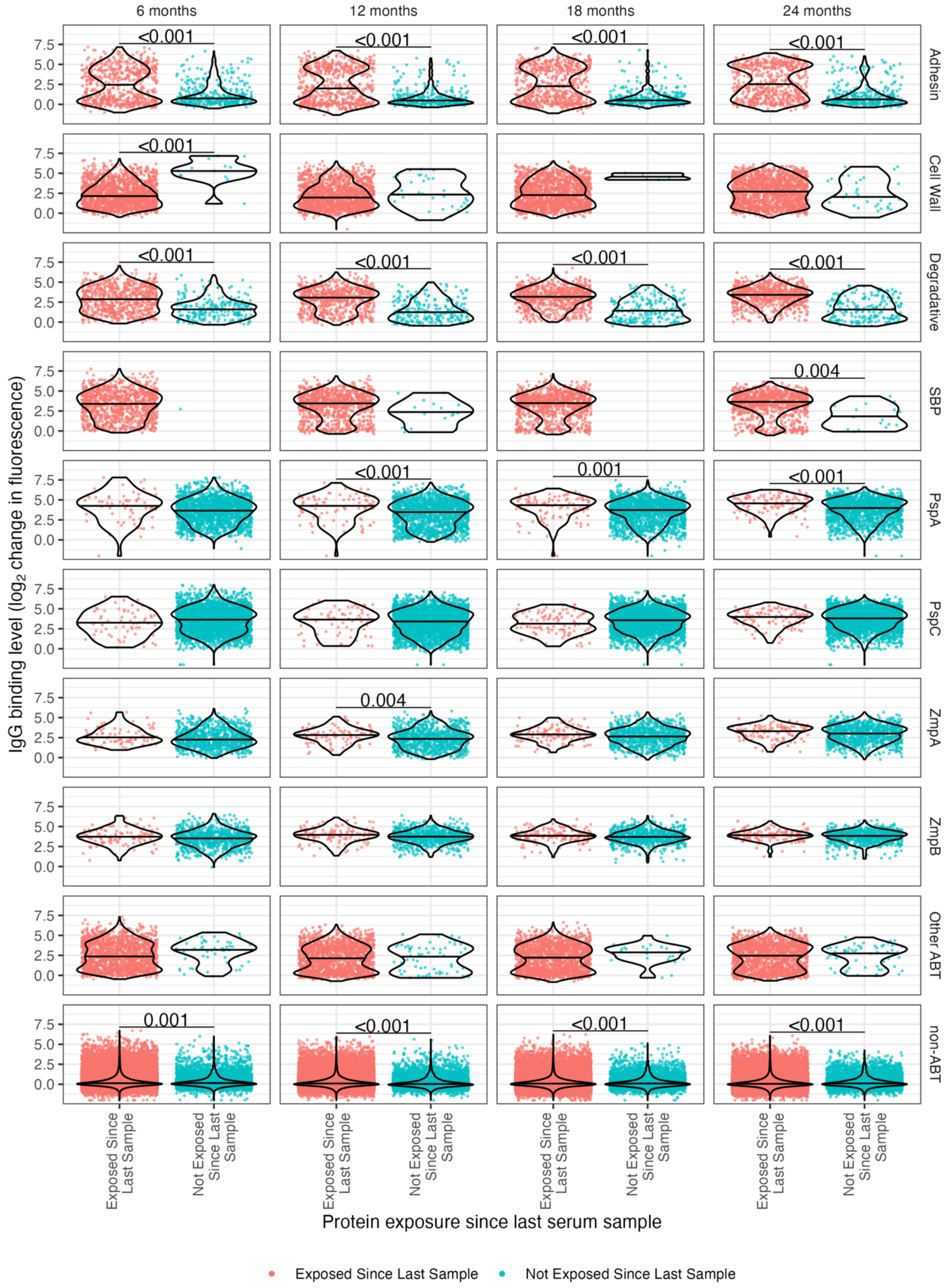

Supplementary Figure 48 Violin plots showing the differences in IgG binding to proteins to which individuals were inferred to have been exposed in the months since the previous serum sample was taken, relative to those proteins for which there was no evidence of exposure from the individual's colonisation history during this period. Data are presented as described for Fig. 5.

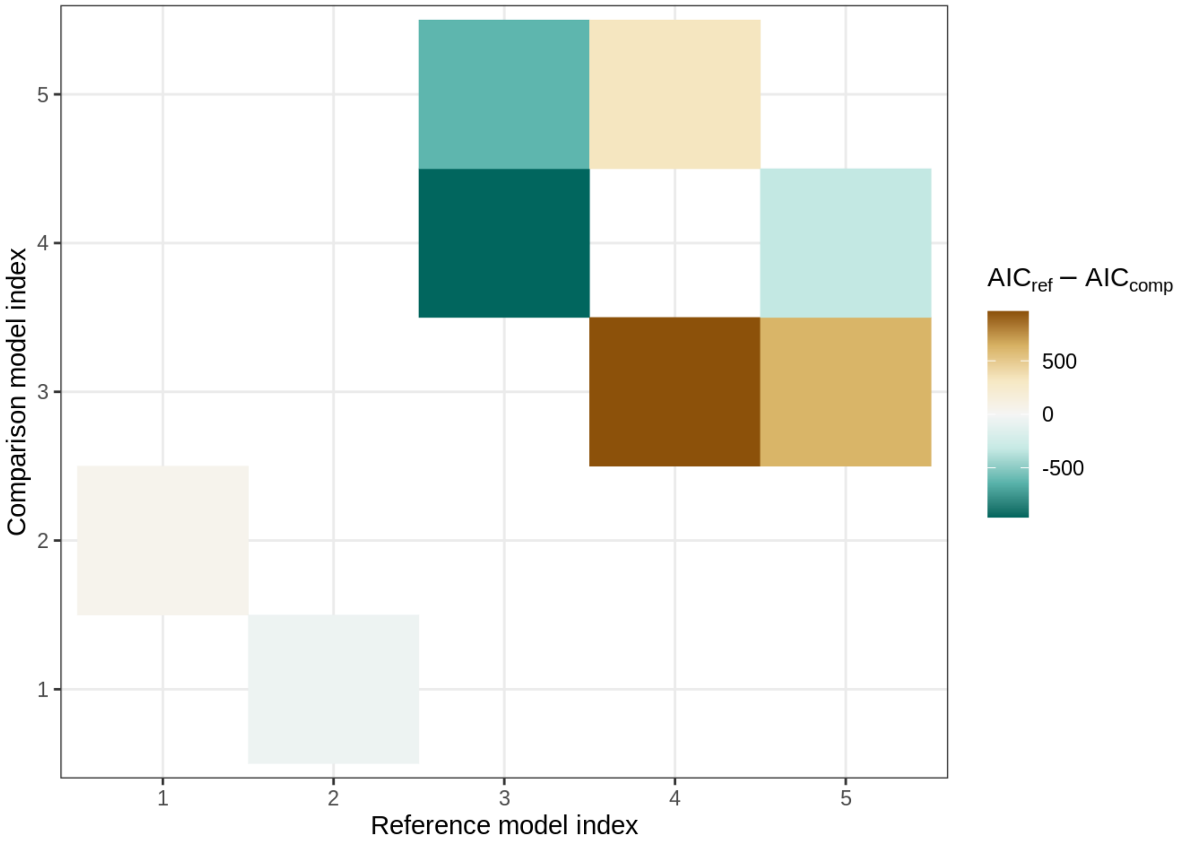

Supplementary Figure 49 Heatmap showing the difference in AIC between linear mixed effects models fitted to the IgG binding data, partitioning ABTs by whether infants had been universally exposed to the proteins at 12 mo (models 1 and 2) or whether the models included terms to account for the partial exposure of the cohort to the protein (models 3, 4 and 5). The models are described in Supplementary Table 5. The colour of each cell shows $AIC_{reference} - AIC_{comparison}$, such that negative values correspond to the reference model having the lower AIC, and therefore being the most appropriate model for the data.

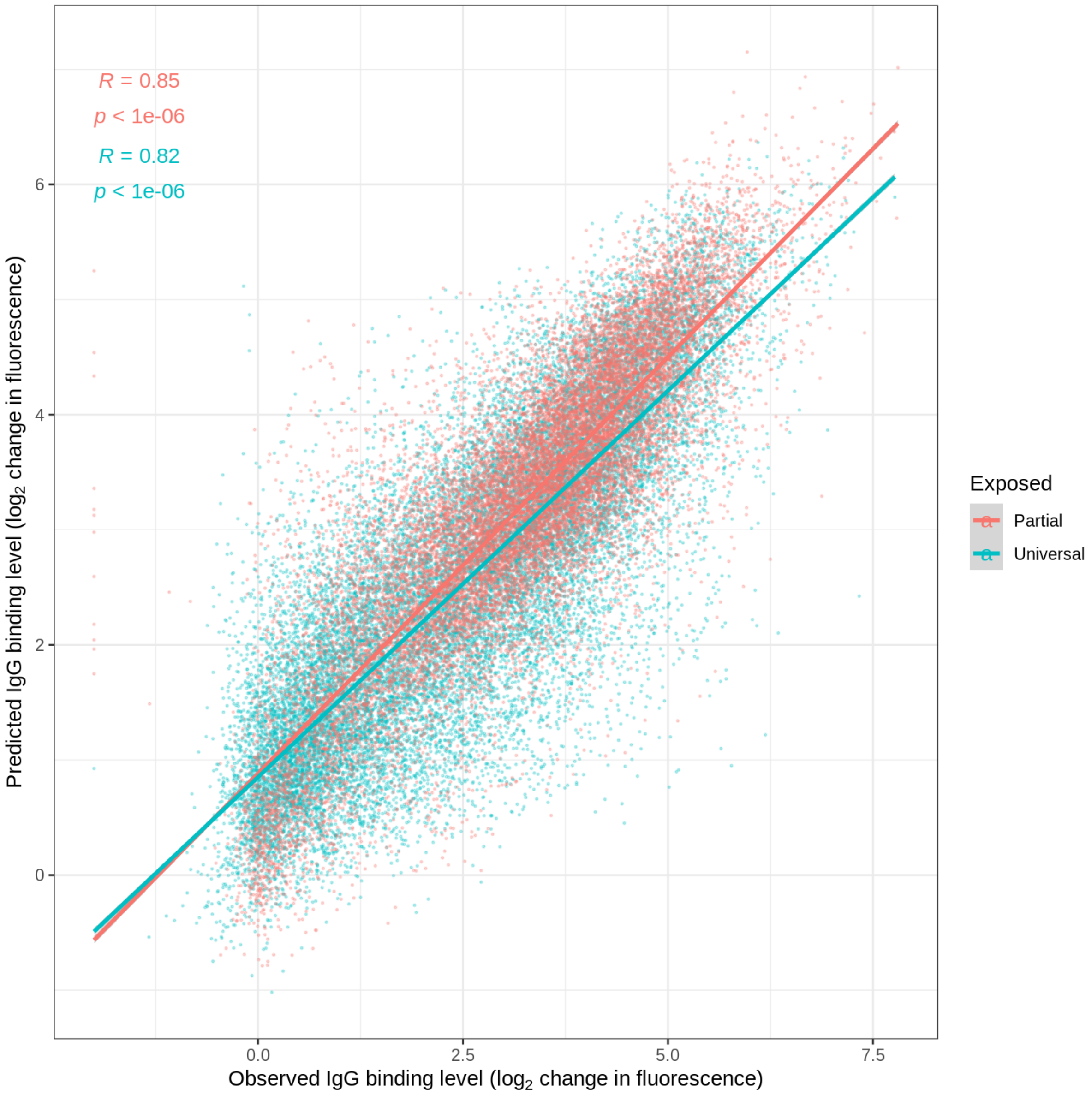

Supplementary Figure 50 Scatterplot showing the relationship between the observed IgG binding levels and those predicted by the best-fitting linear mixed-effects models when ABTs were categorised by whether exposure was universal, or partial, across the cohort at 12 mo. Points are coloured by the type of model used to generate the prediction. The best-fitting linear relationship between the observed and predicted values are shown for each model type.

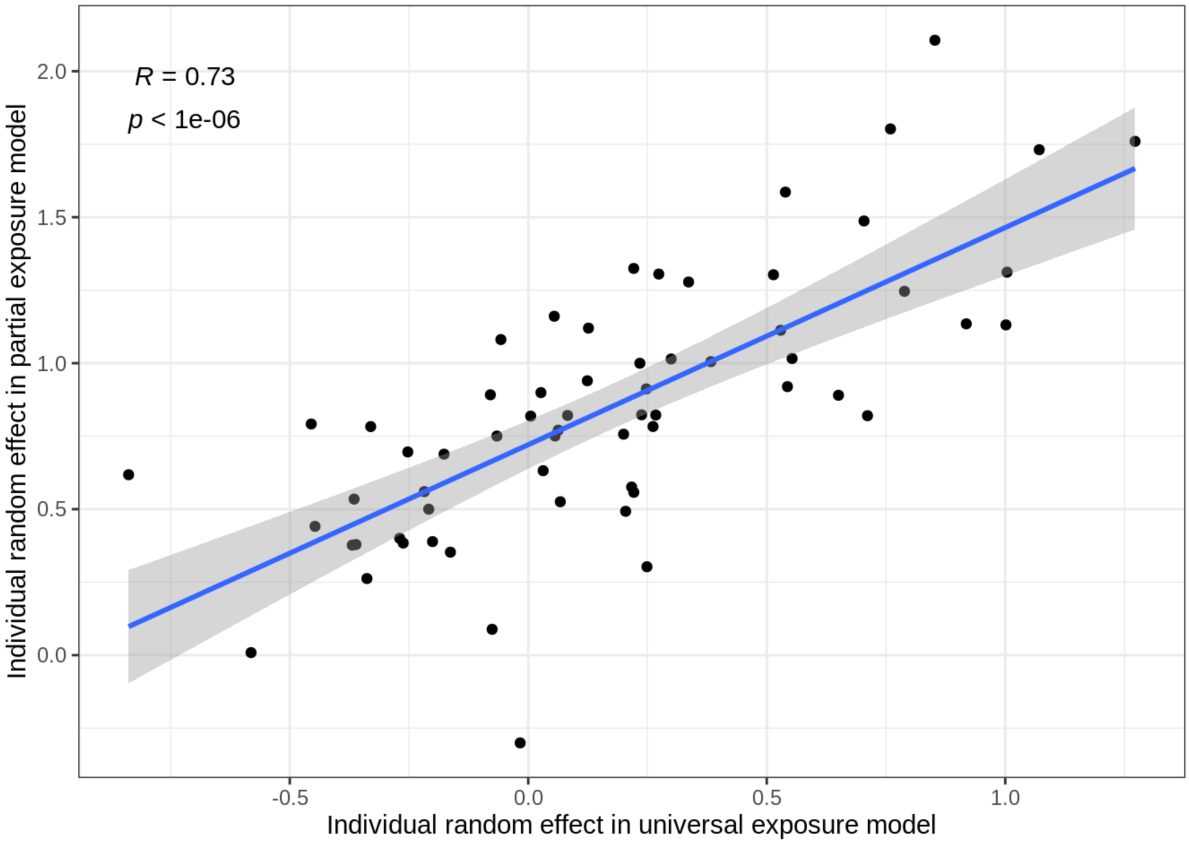

Supplementary Figure 51 Scatterplot comparing the estimated random effect terms for individuals from the linear mixed-effects models fitted to IgG binding data to ABTs to which exposure was either universal, or partial, across the cohort at 12 mo. Each point is a separate individual. The blue line shows the best-fitting linear model of the data, with the grey shaded region representing the 95% confidence interval. The strong positive correlation shows both models generate consistent estimations of the strength of the antibody response mounted by each individual, despite the distinct model structures being independently fitted to separate partitions of the data.

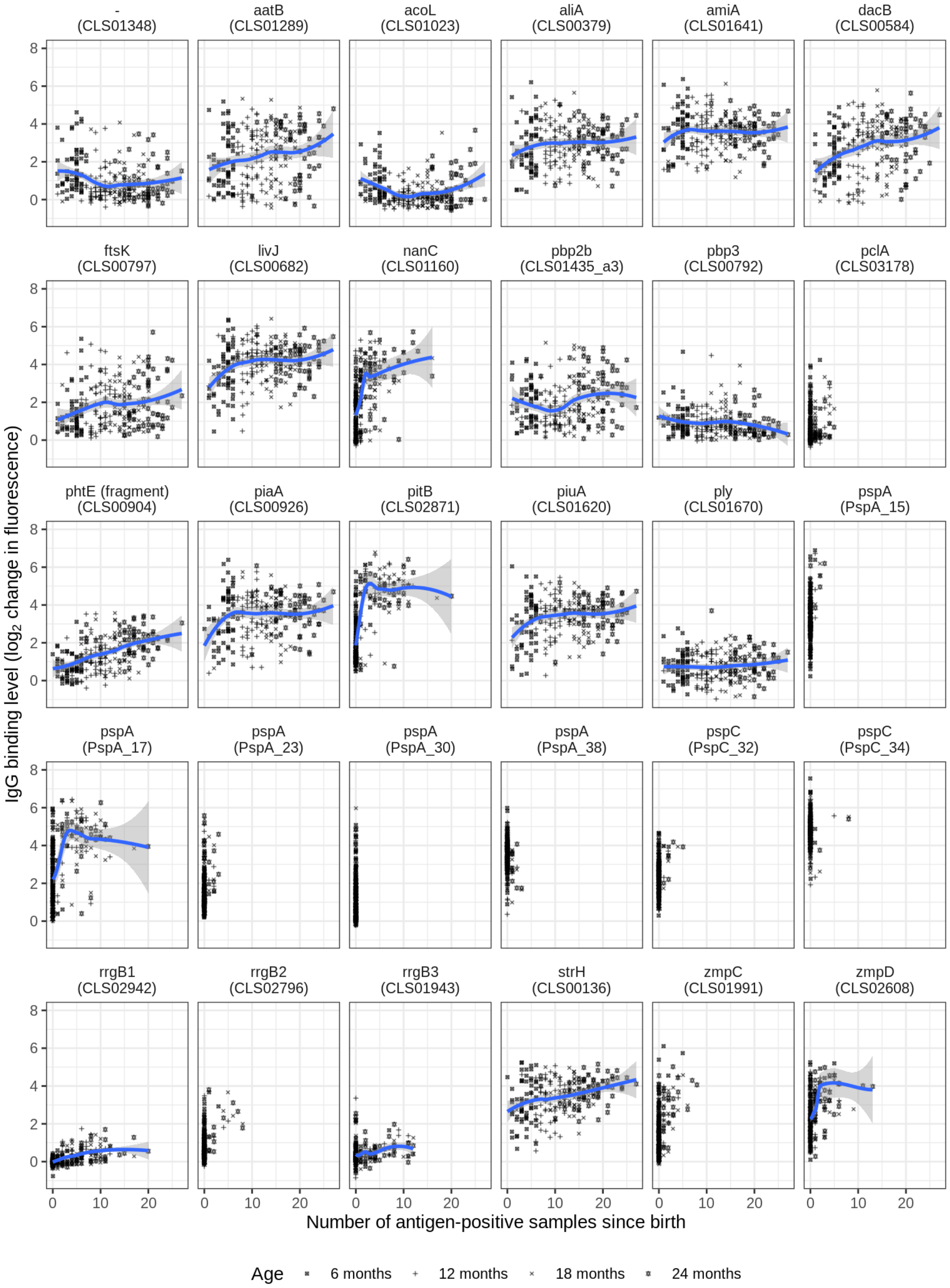

Supplementary Figure 52 Scatterplots showing the relationship between IgG binding to proteins, and the number of times a pneumococcus encoding the protein was detected in the nasopharynx of an individual. Each facet represents a different protein, selected either because IgG responses to these proteins correlated poorly with earlier timepoints (Fig. 4), modelling identified a substantial increase in IgG binding following exposure (Fig. 6), or the proteins have previously been proposed as candidates for inclusion in protein-based vaccines. The blue lines summarise the data through Loess smoothing, with the grey shaded region showing the 95% confidence interval.

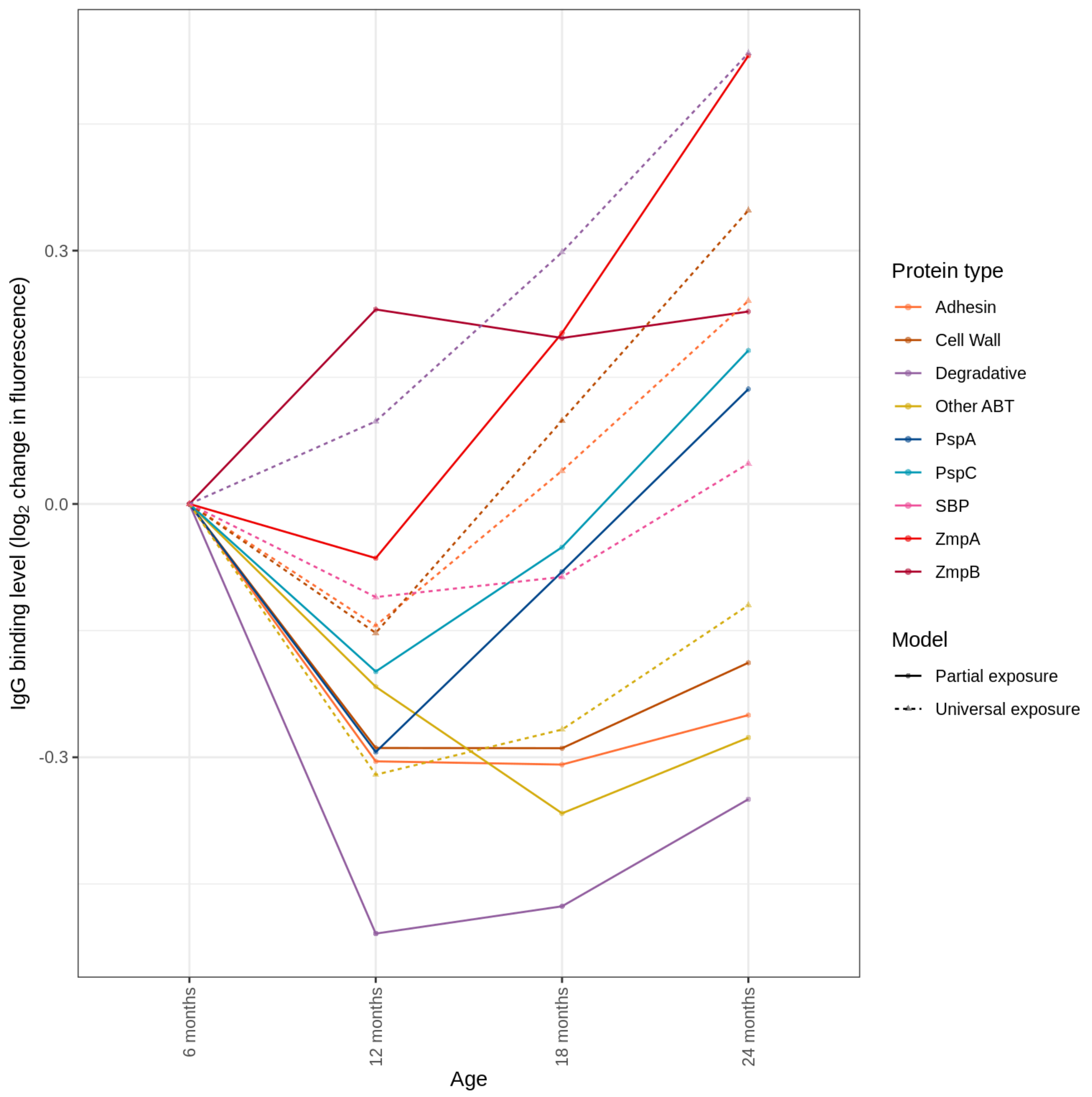

Supplementary Figure 53 Line graph showing the changes in IgG binding to ABTs with age estimated by the linear mixed-effects models fitted to proteins to which exposure at 12 mo was either universal or partial across the cohort. All lines were adjusted to have a vertical axis intercept of zero by subtracting the IgG binding estimate at 6 mo from all datapoints. The colour indicates the type of ABT. The line type shows the model type from which the trends were inferred. Only data from 6 mo or older are shown, as prior exposure to proteins could not be inferred for the maternal antibody profiles represented by the maternal birth or umbilical cord samples.

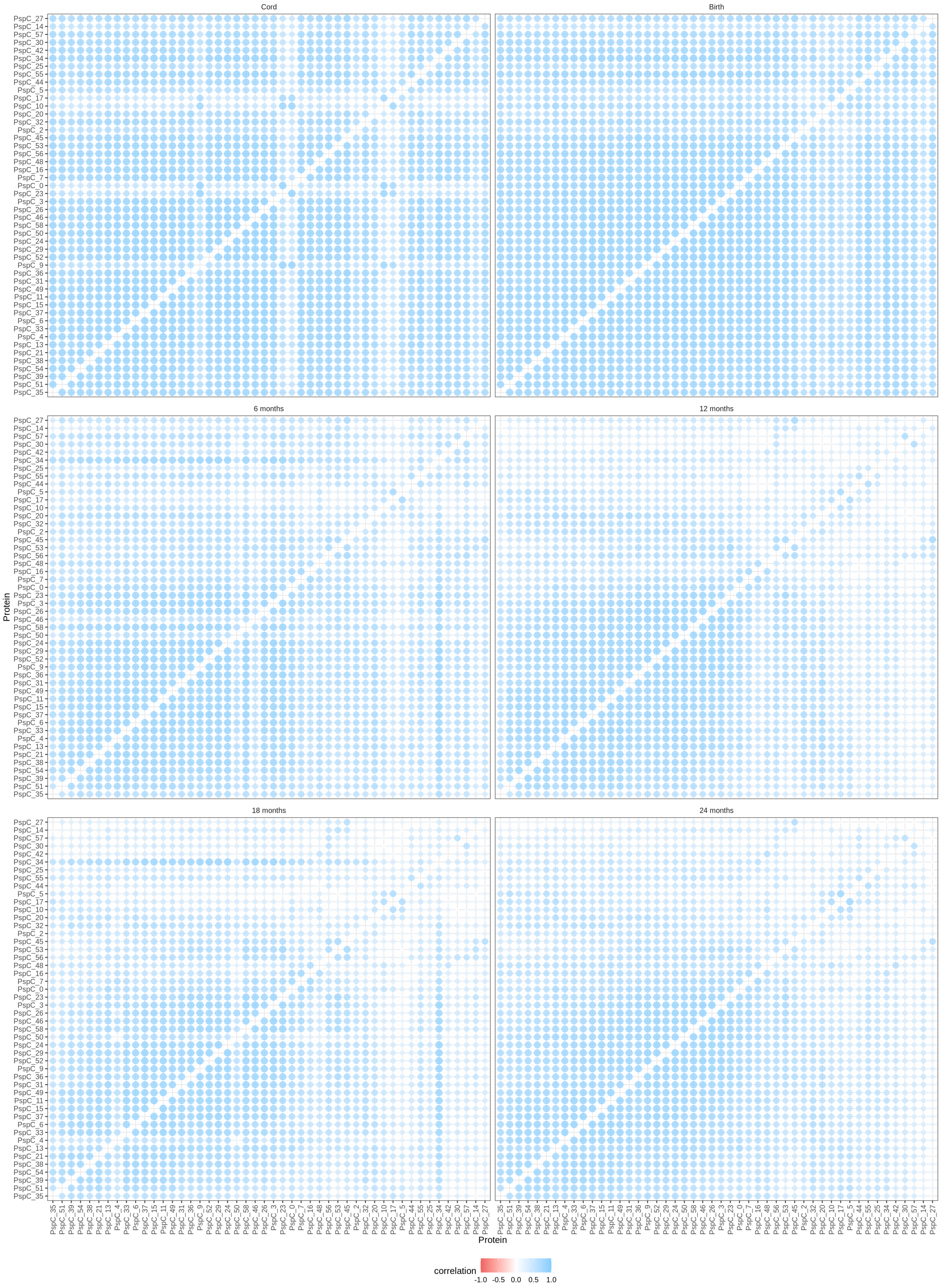

Supplementary Figure 54 Scatterplot showing the correlation between IgG binding to different PspC variants across individuals. The colour and size of each point shows the strength and direction of the correlation. Each plot shows equivalent data across different timepoints.

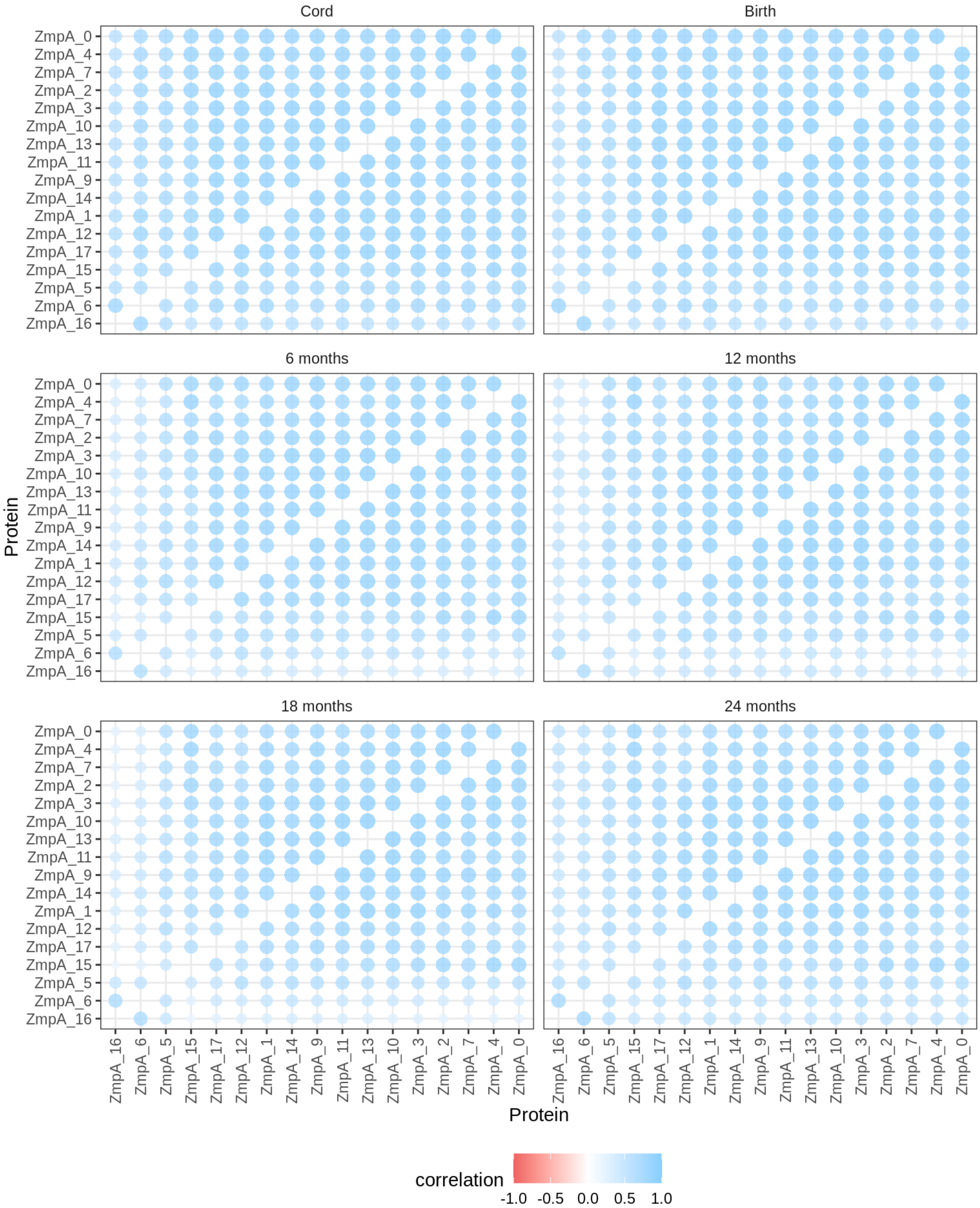

Supplementary Figure 55 Scatterplot showing the correlation between IgG binding to different ZmpA variants across individuals. The colour and size of each point shows the strength and direction of the correlation. Each plot shows equivalent data across different timepoints.

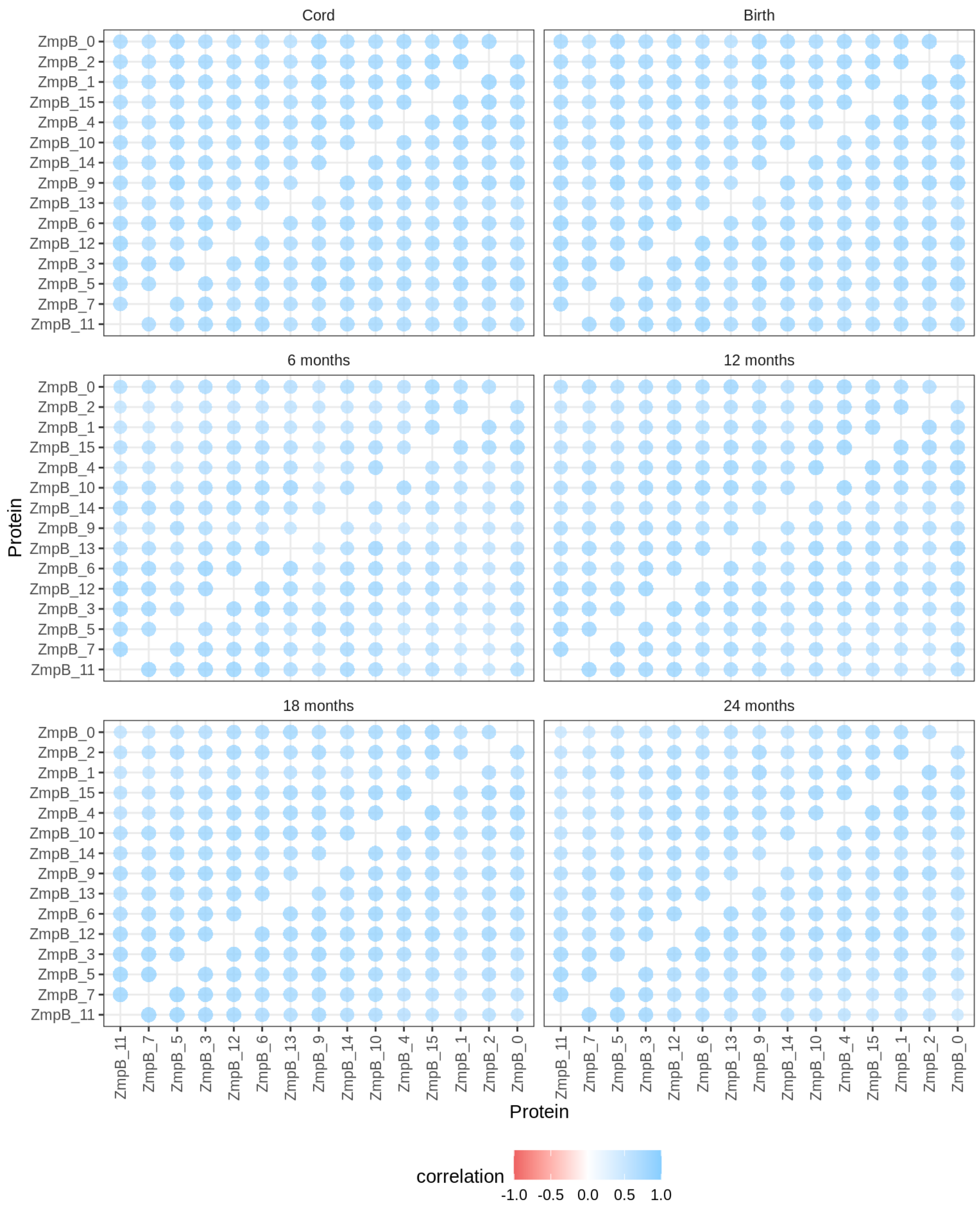

Supplementary Figure 56 Scatterplot showing the correlation between IgG binding to different ZmpB variants across individuals. The colour and size of each point shows the strength and direction of the correlation. Each plot shows equivalent data across different timepoints.

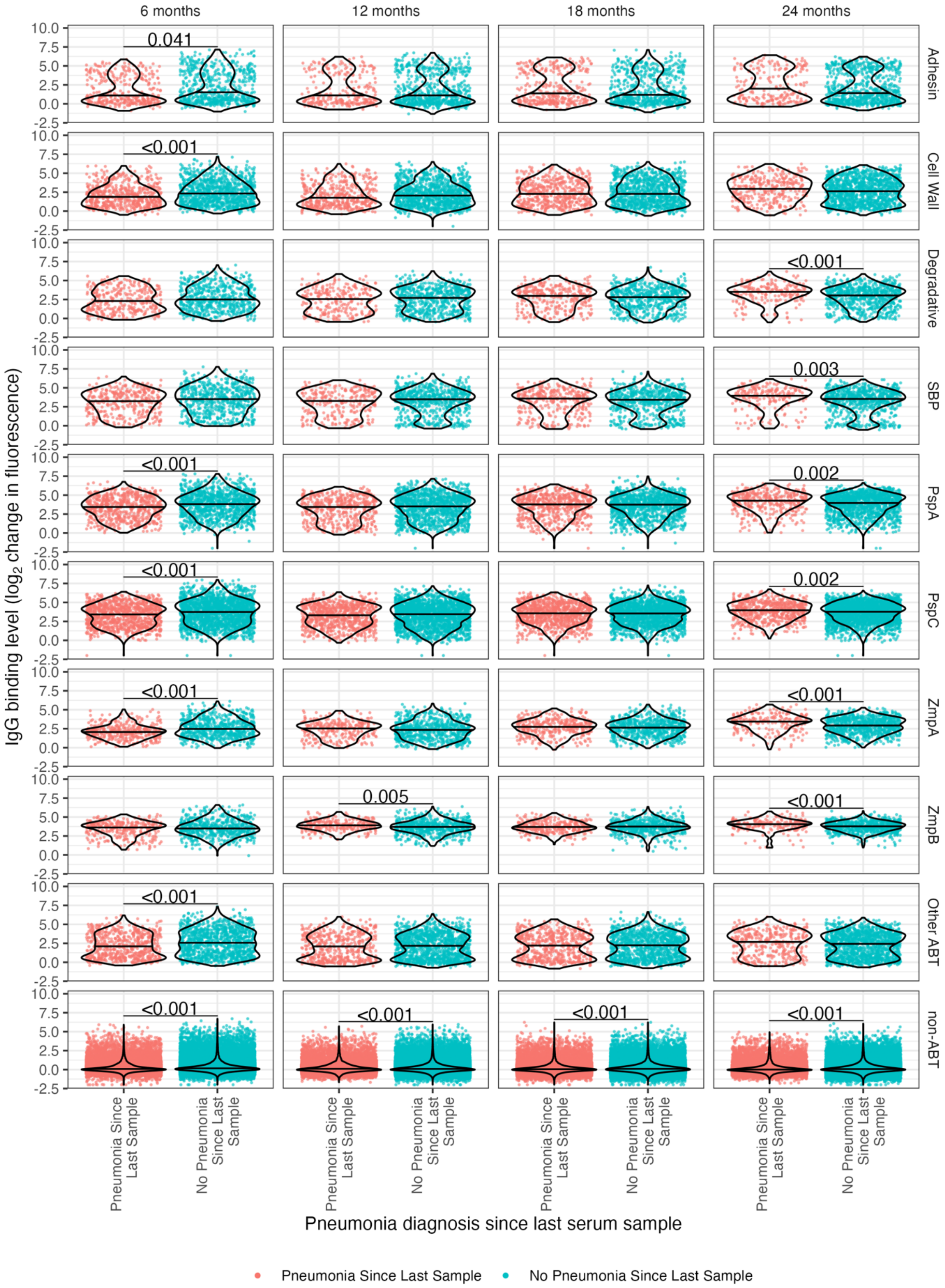

Supplementary Figure 57 Violin plots showing the differences in IgG binding to proteins in individuals who suffered at least one case of pneumonia in the interval since the last serological sample, relative to those in whom no such infection was diagnosed. Data are shown as in Fig. 5.

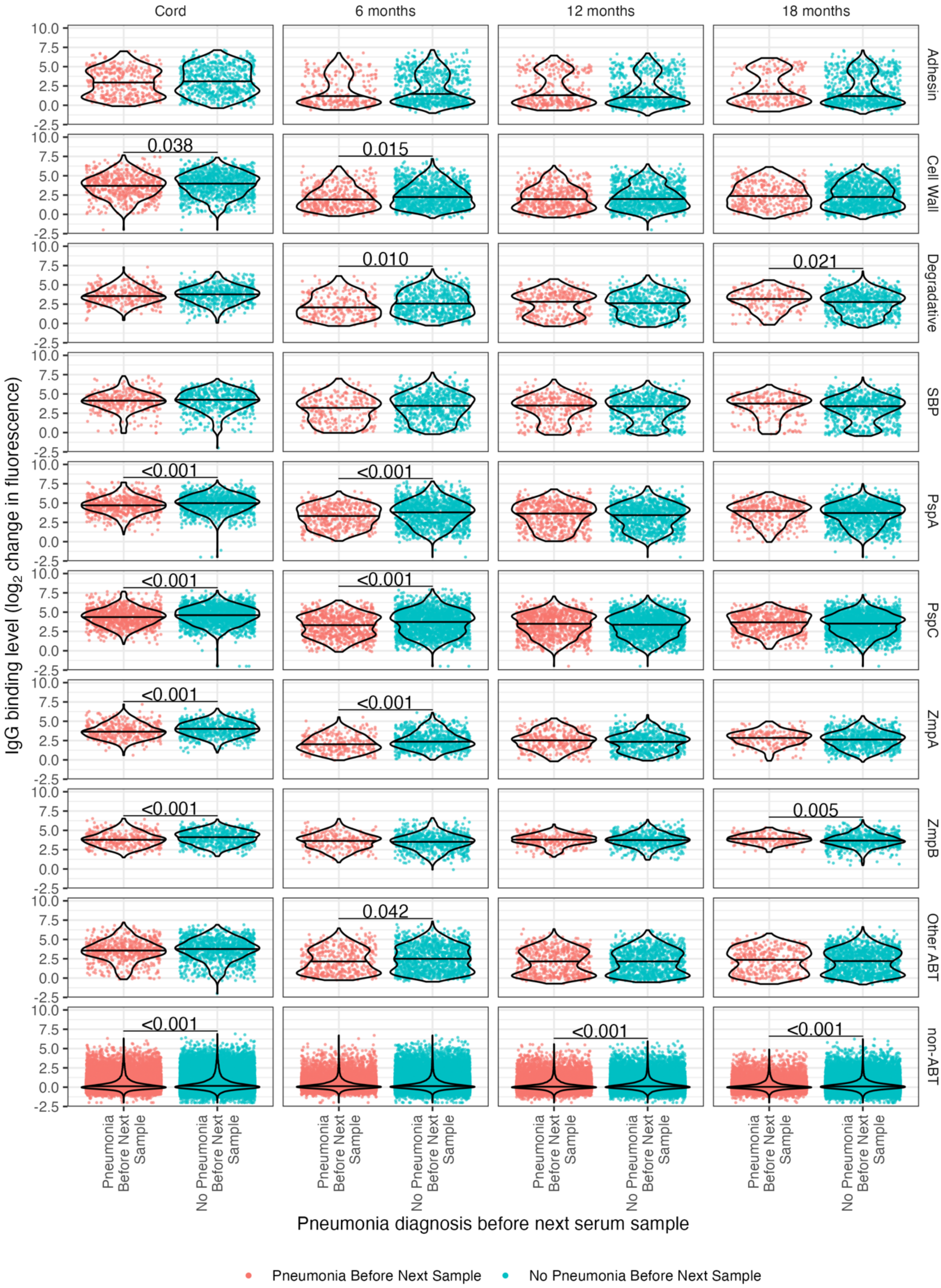

Supplementary Figure 58 Violin plots showing the differences in IgG binding to proteins in individuals who suffered at least one case of pneumonia in the period before the next serological sample, relative to those in whom no such infection was diagnosed. Data are shown as in Fig. 5.

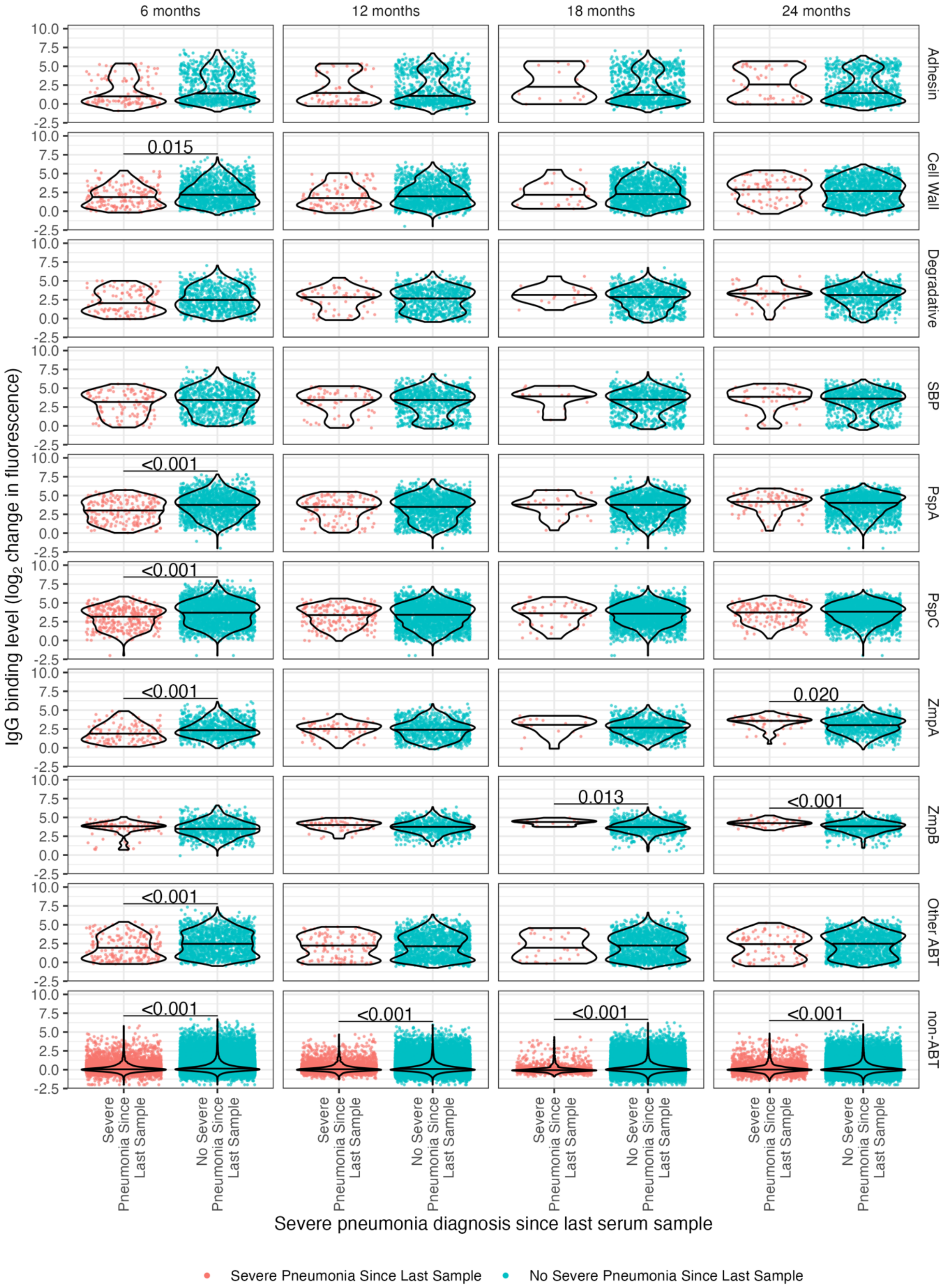

Supplementary Figure 59 Violin plots showing the differences in IgG binding to proteins in individuals who suffered at least one case of severe pneumonia in the interval since the last serological sample, relative to those in whom no such infection was diagnosed. Data are shown as in Fig. 5.

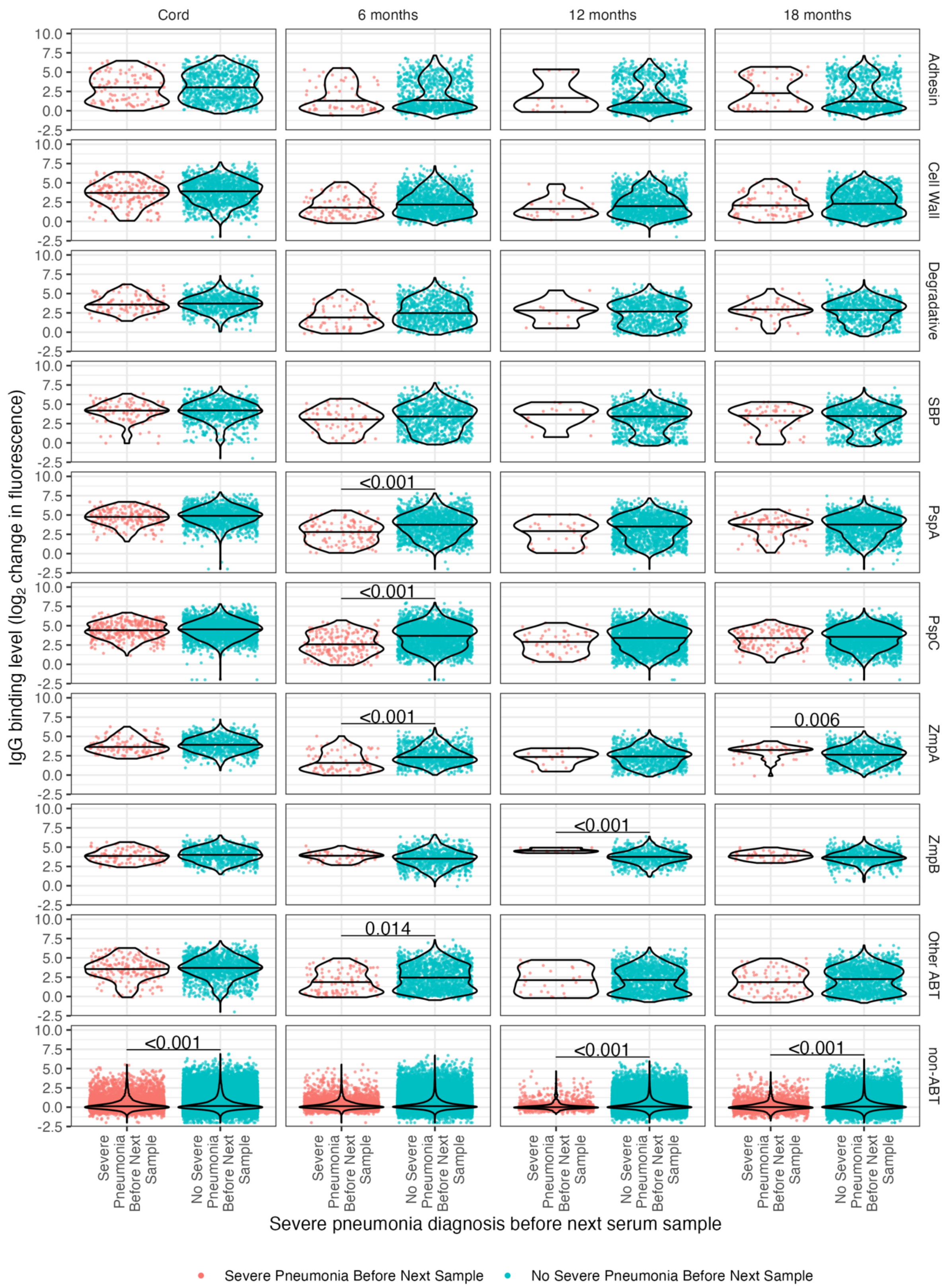

Supplementary Figure 60 Violin plots showing the differences in IgG binding to proteins in individuals who suffered at least one case of severe pneumonia in the period before the next serological sample, relative to those in whom no such infection was diagnosed. Data are shown as in Fig. 5.

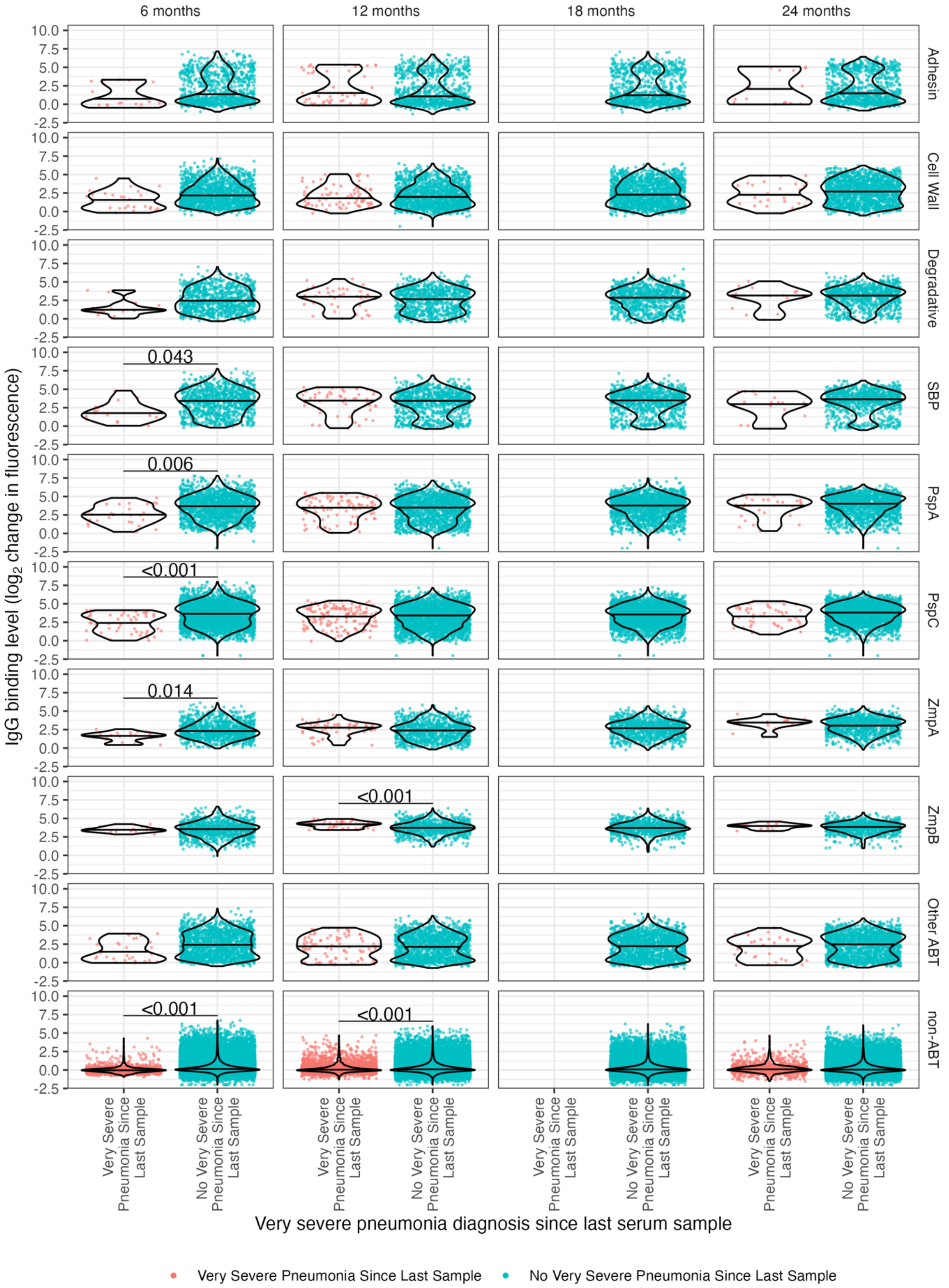

Supplementary Figure 61 Violin plots showing the differences in IgG binding to proteins in individuals who suffered at least one case of very severe pneumonia in the interval since the last serological sample, relative to those in whom no such infection was diagnosed. Data are shown as in Fig. 5.

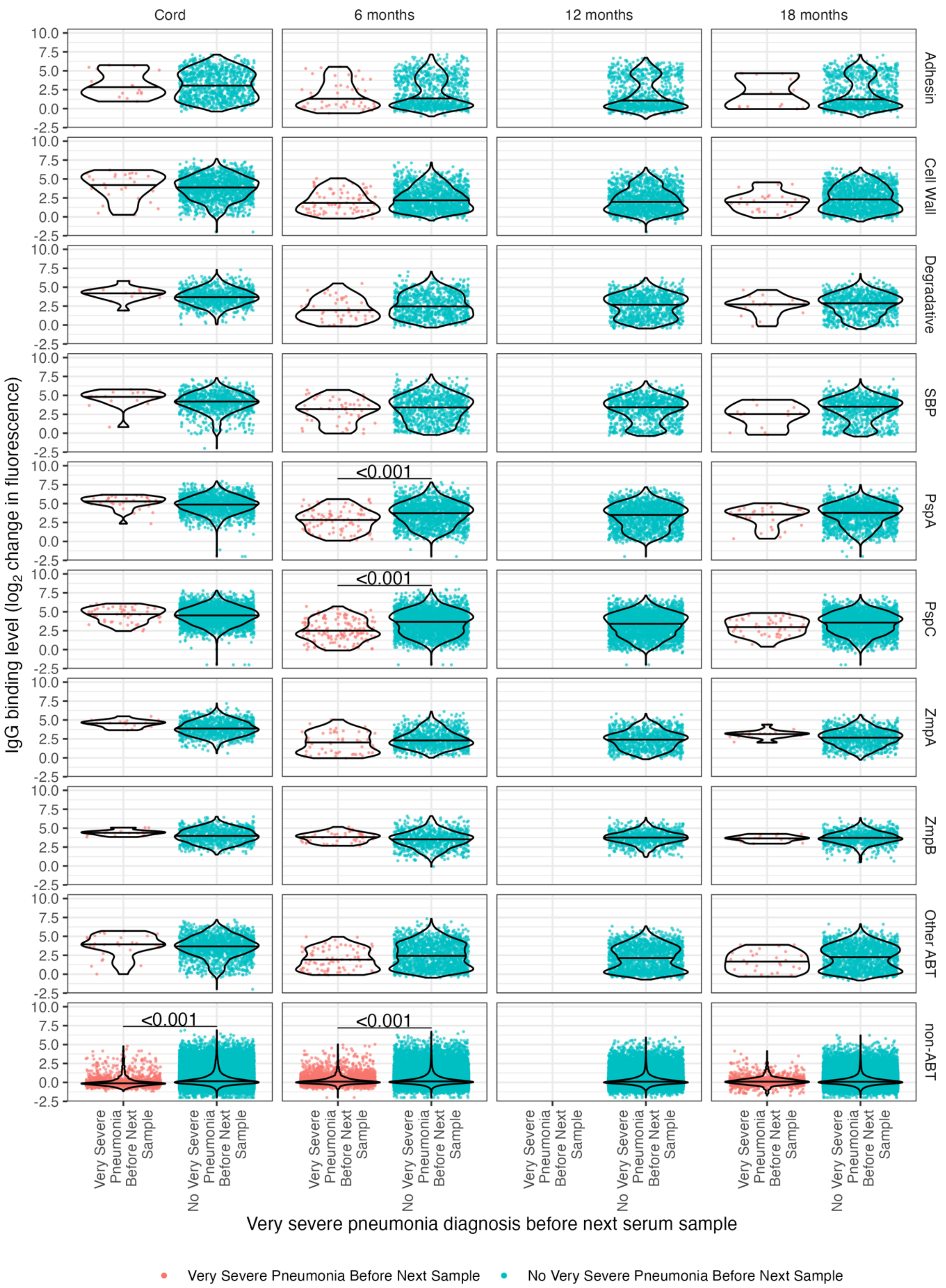

Supplementary Figure 62 Violin plots showing the differences in IgG binding to proteins in individuals who suffered at least one case of very severe pneumonia in the period before the next serological sample, relative to those in whom no such infection was diagnosed. Data are shown as in Fig. 5.

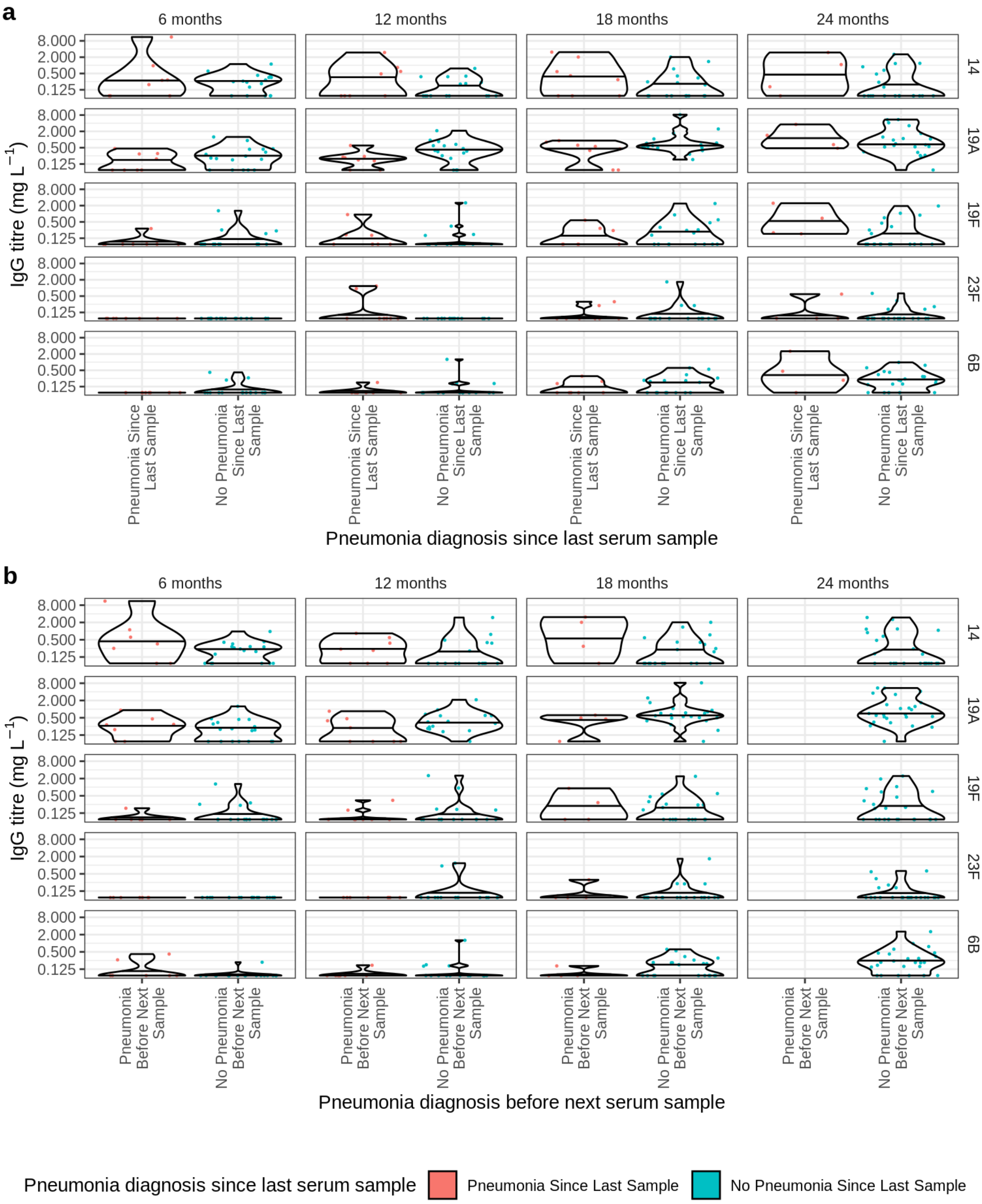

Supplementary Figure 63 Violin plots showing the relationship between anti-capsular IgG and disease. The titres of IgG recognising five pneumococcal capsules were previously measured with ELISA assays by Turner *et al*. These were combined with the epidemiological data on cases of pneumonia. The violin plots show the anti-capsular IgG levels in individuals who did, and did not, not suffer pneumonia (a) in the six months after the serum sample, and (b) the six months before the serum sample. These data provide little evidence of anti-capsular IgG levels providing substantial protection against pneumonia. This concords with it

being highly unlikely that such serotype-specific responses would significantly protect against the incidence of all-cause pneumonia.

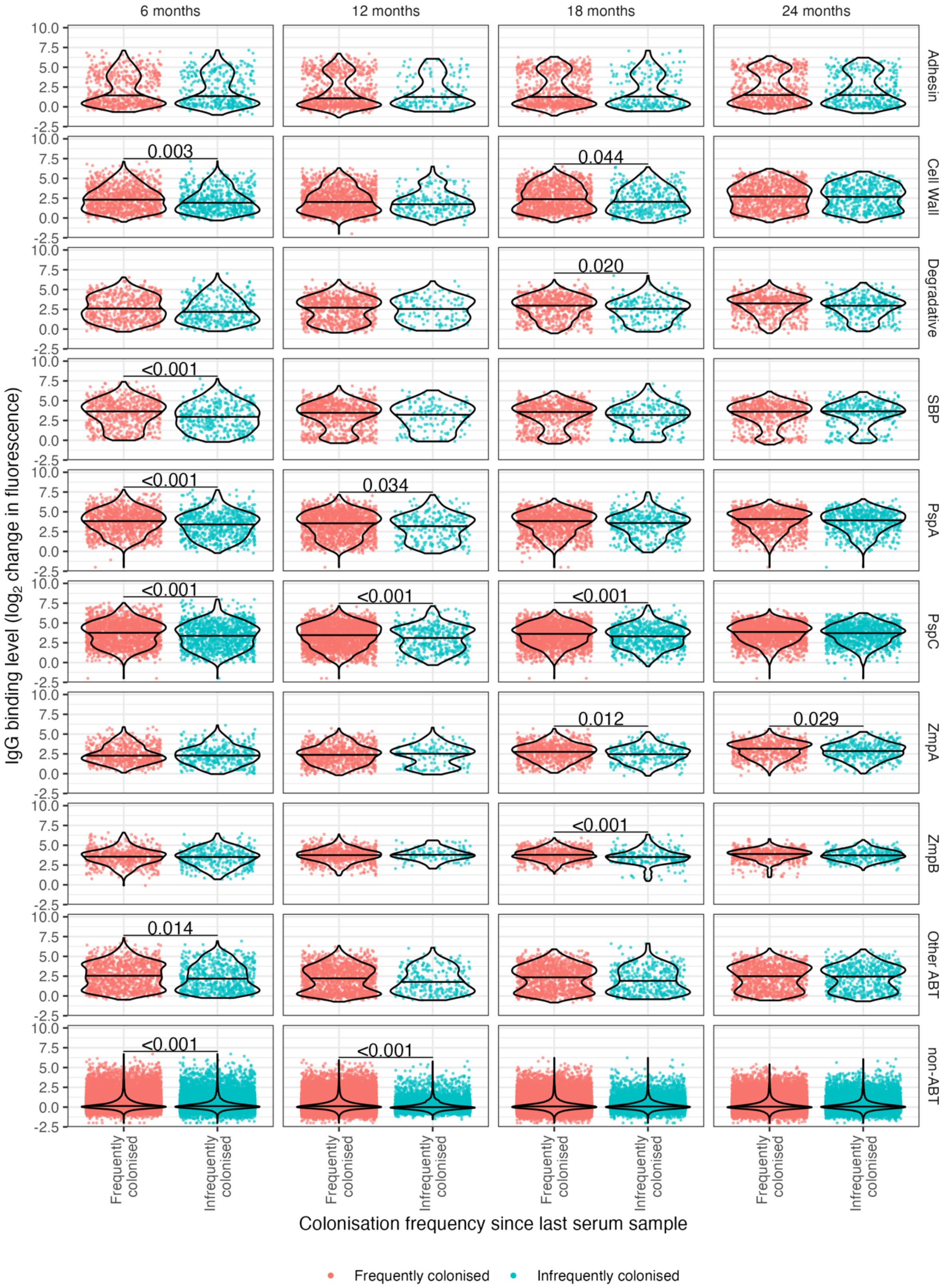

Supplementary Figure 64 Violin plots showing the differences in IgG binding to proteins in individuals who were frequently colonised by pneumococci in the interval since the last serological sample, relative to those who were infrequently colonised. Data are shown as in Fig. 5.

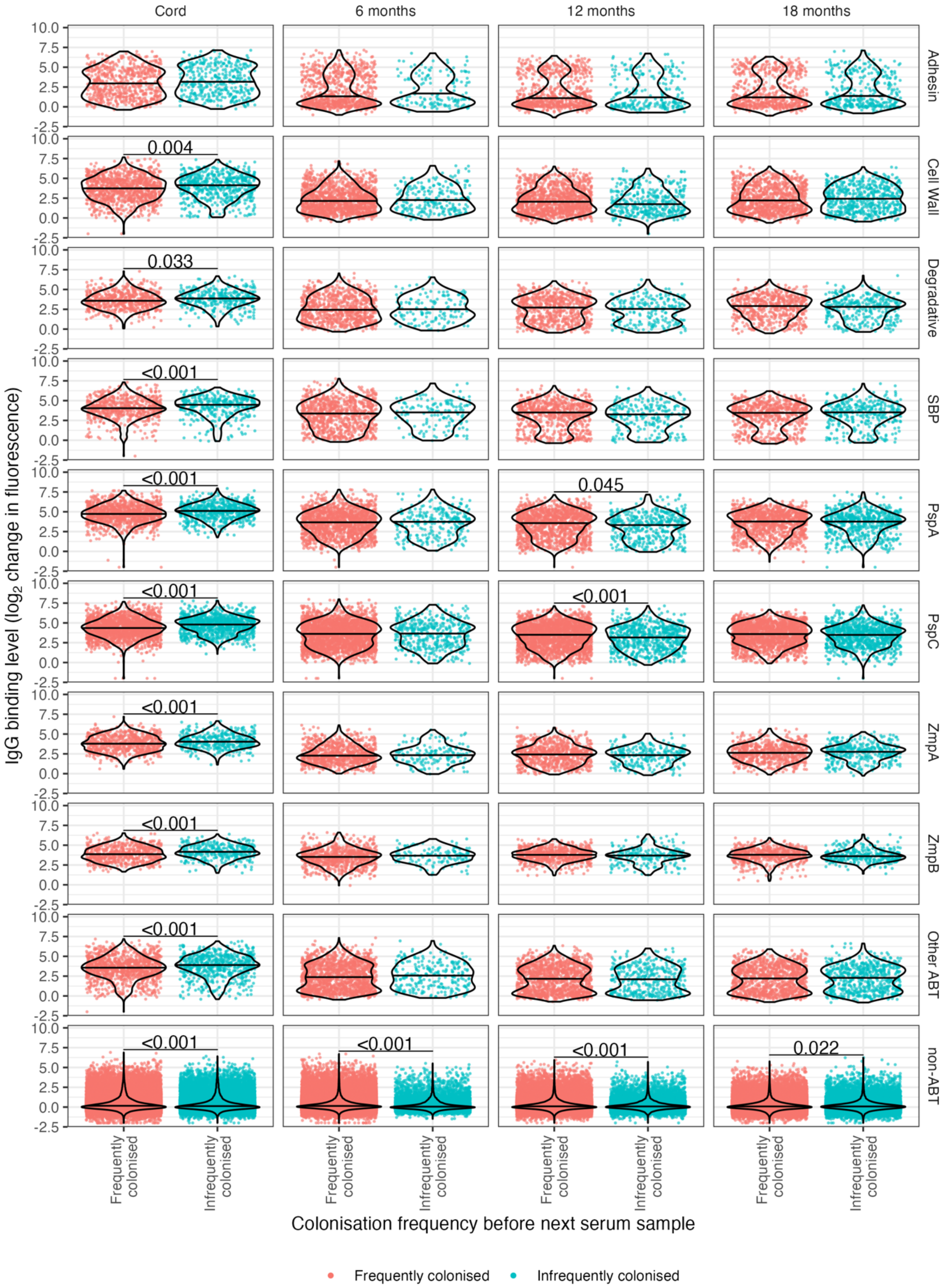

Supplementary Figure 65 Violin plots showing the differences in IgG binding to proteins in individuals who were frequently colonised by pneumococci in the period before the next serological sample, relative to those who were infrequently colonised. Data are shown as in Fig. 5.

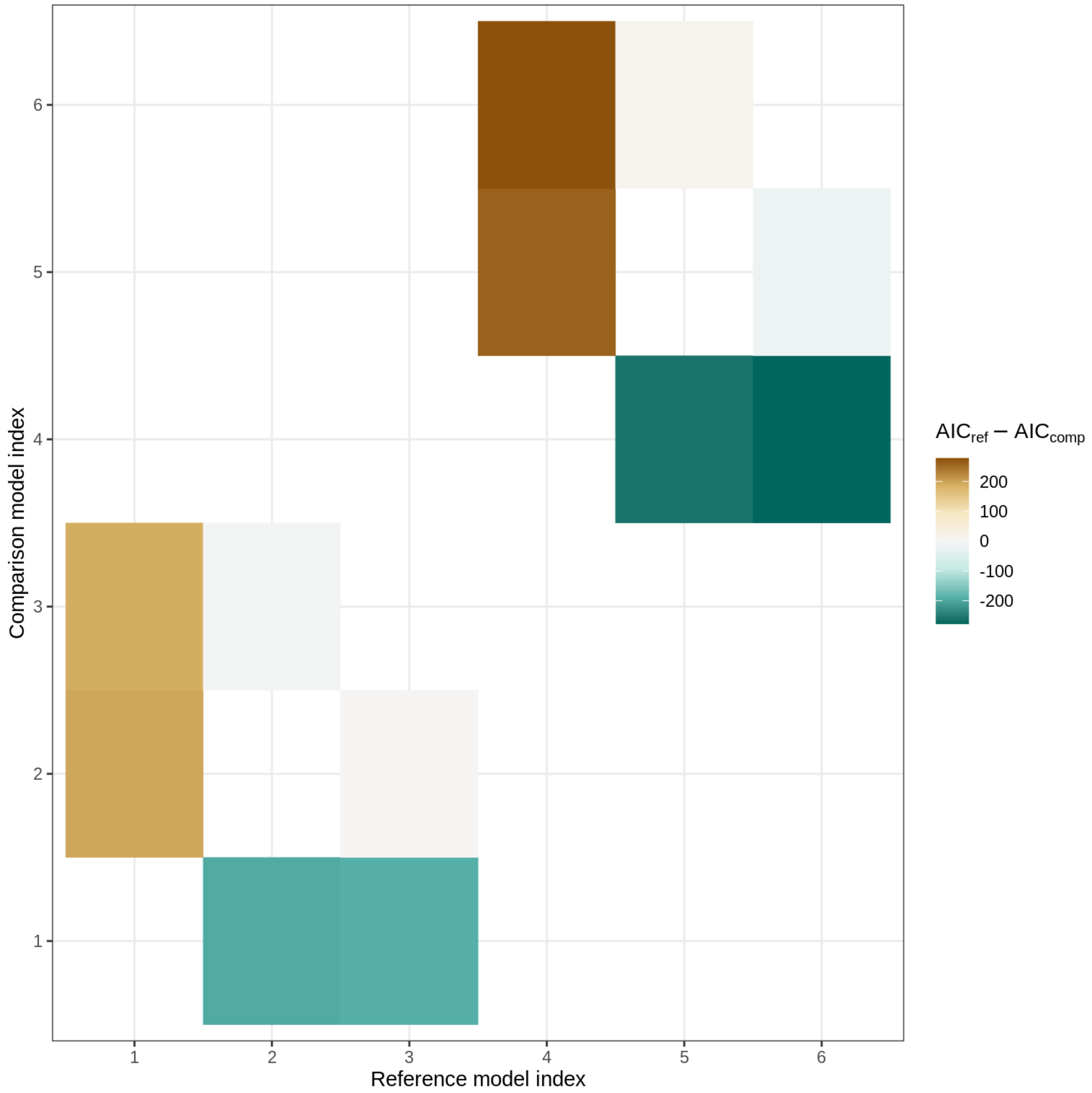

Supplementary Figure 66 Heatmap showing the difference in AIC between linear mixed effects models fitted to the IgG binding and clinical pneumonia episode data, partitioning ABTs by whether infants had been universally exposed to the proteins at 12 mo (models 1, 2 and 3) or whether the models included terms to account for the partial exposure of the cohort to the proteins (models 4, 5 and 6). The models are described in Supplementary Table 6. The colour of each cell shows $AIC_{reference} – AIC_{comparison}$, such that negative values correspond to the reference model having the lower AIC, and therefore being the most appropriate model for the data.

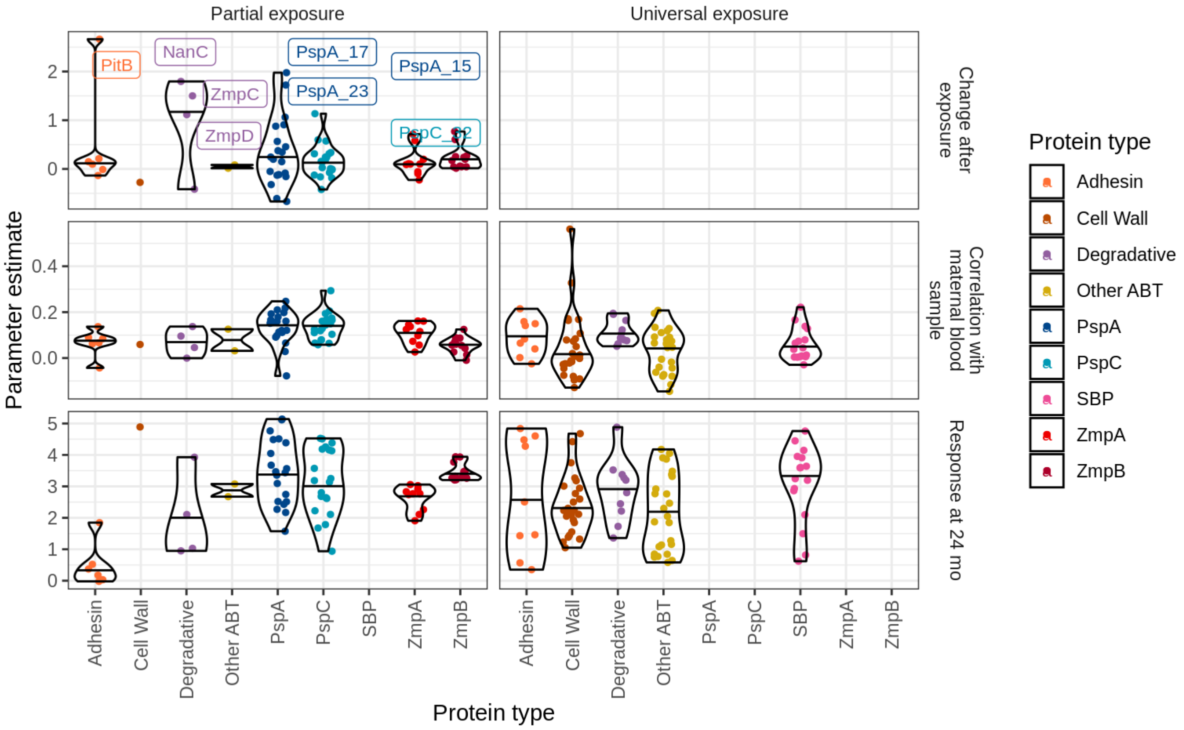

Supplementary Figure 67 Modelling the effects of exposure, maternal antibody levels and clinical pneumonia on IgG responses to ABTs. These parameter estimates are plotted as described for Fig. 6, although these models differ in featuring a fixed effect term accounting for the age-dependent effect of a clinical pneumonia episode on IgG binding.

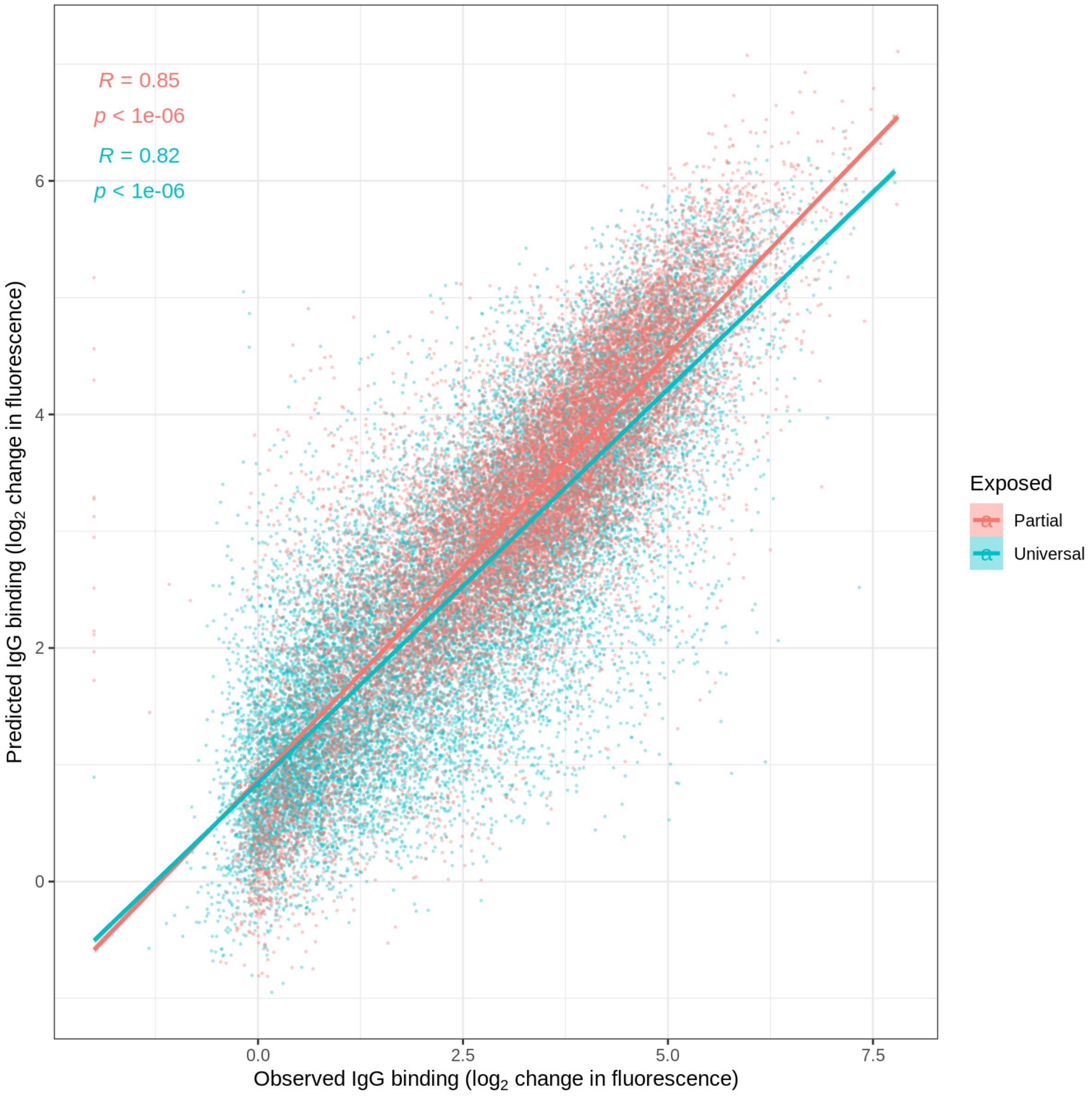

Supplementary Figure 68 Scatterplot showing the relationship between the observed IgG binding levels and those predicted by the best-fitting linear mixed-effects models including the effects of a clinical pneumonia episode on IgG binding. The two models were separately fitted to subsets of the data, split by whether exposure to ABTs was universal, or partial across the cohort, at 12 mo. Points are coloured by the type of model used to generate the prediction. The best-fitting linear relationship between the observed and predicted values Supare shown for each model type.

Supplementary Tables

Supplementary Table 1 Linear mixed-effects models used to analyse variation in IgG binding levels to proteins ($IgG_{Protein}$), measured as the base two logarithm of the change in mean fluorescence intensity on the array. The models considered the variables $Age_{Individual}$ (of the individual when the sample was taken); $Type_{Protein}$ (based on the functional classification in Fig. 2); Individual (referring to the member of the cohort), and $Cluster_{Individual,Age}$ (the immune cluster to which the individual was assigned at the corresponding age). The notation (1|variable) is used to denote random effects; all other terms were fixed effects. Asterisks denote interaction terms. The final columns show the Akaike Information Criterion (AIC) and the Bayesian Information Criterion (BIC) of the fit of the model to the data. The numerical index of the model corresponds to the plot of AIC values in Supplementary Fig. 18. Model 13 was selected for further analysis of the IgG responses, as it had the lowest AIC value.

Index	Model structure	AIC	BIC
1	$IgG_{Protein} \sim Age_{Individual} + Type_{Protein} + (1\|Individual)$	1618120	1618315
2	$IgG_{Protein} \sim Age_{Individual} + Type_{Protein} + Cluster_{Individual,Age} + (1\|Individual)$	1610942	1611193
3	$IgG_{Protein} \sim Age_{Individual} + Type_{Protein} + (1\|Cluster_{Individual,Age}) + (1\|Individual)$	1610984	1611189
4	$IgG_{Protein} \sim Age_{Individual} + Type_{Protein} + Cluster_{Individual,Age} * Type + (1\|Individual)$	1573339	1574103
5	$IgG_{Protein} \sim Age_{Individual} + (1\|Type_{Protein}) + (1\|Individual)$	1618207	1618310
6	$IgG_{Protein} \sim Age_{Individual} + (1\|Type_{Protein}) + Cluster_{Individual,Age} + (1\|Individual)$	1611029	1611189
7	$IgG_{Protein} \sim Age_{Individual} + (1\|Type_{Protein}) + (1\|Cluster_{Individual,Age}) + (1\|Individual)$	1611068	1611182
8	$IgG_{Protein} \sim Age_{Individual} + (1\|Type_{Protein}) + Cluster_{Individual,Age} * Type_{probe} + (1\|Individual)$	1573341	1574117
9	$IgG_{Protein} \sim Age_{Individual} * Type_{Protein} + (1\|Individual)$	1595479	1596186
10	$IgG_{Protein} \sim Age_{Individual} * Type_{Protein} + Cluster_{Individual,Age} + (1\|Individual)$	1588051	1588816
11	$IgG_{Protein} \sim Age_{Individual} * Type_{Protein} + (1\|Cluster_{Individual,Age}) + (1\|Individual)$	1588093	1588812
12	$IgG_{Protein} \sim Age_{Individual} * Type_{Protein} + Cluster_{Individual,Age} * Type_{probe} + (1\|Individual)$	1570527	1571805
13	$IgG_{Protein} \sim Age_{Individual} * Type_{Protein} * Cluster_{Individual,Age} + (1\|Individual)$	1569316	1571849

Supplementary Table 2 Linear mixed-effects models used to test the power of environmental and physiological differences to explain variation in IgG binding levels to proteins ($IgG_{Protein}$). The best-fitting model structure in Supplementary Table 1 was modified to replace an individual's immune cluster at a specific age with an age-independent, individual-specific variable: maternal ethnicity ($Ethnicity_{Mother}$); whether the child's mother smoked ($Smoking_{Mother}$); the sex of the child ($Sex_{Individual}$); the child's birth weight ($Weight_{Birth}$); whether the child's home was heated by a fire ($Fire_{Individual}$), or the fuel used by the house fire ($Fuel_{Individual}$). Asterisks denote interaction terms. The final columns show the AIC and BIC of the fit of the model to the data. The numerical index of the model corresponds to the plot of AIC values in Supplementary Fig. 20. Model 1 was the most appropriate structure to describe the data, based on both the AIC and BIC values.

Index	Model structure	AIC	BIC
1	$IgG_{Protein} \sim Age_{Individual} * Type_{Protein} * Cluster_{Individual,Age} + (1\|Individual)$	1569316	1571849
2	$IgG_{Protein} \sim Age_{Individual} * Type_{Protein} * Ethnicity_{Mother} + (1\|Individual)$	1592281	1595042
3	$IgG_{Protein} \sim Age_{Individual} * Type_{Protein} * Smoking_{Mother} + (1\|Individual)$	1593818	1595210
4	$IgG_{Protein} \sim Age_{Individual} * Type_{Protein} * Sex_{Individual} + (1\|Individual)$	1593707	1595099
5	$IgG_{Protein} \sim Age_{Individual} * Type_{Protein} * Weight_{Birth} + (1\|Individual)$	1593561	1594953
6	$IgG_{Protein} \sim Age_{Individual} * Type_{Protein} * Fire_{Individual} + (1\|Individual)$	1594907	1596300
7	$IgG_{Protein} \sim Age_{Individual} * Type_{Protein} * Fuel_{Individual} + (1\|Individual)$	1593035	1595112

Supplementary Table 3 Isolation of pneumococci from nasopharyngeal swabs taken from the cohort of households studied in this analysis, including both mothers and children. Samples are categorised by whether they were taken as part of routine monthly sampling of healthy individuals, or taken during an episode of pneumonia. Microbiological culturing of the bacteria that grew from each swab was used to assess whether pneumococci were present.

Nasopharyngeal sample type	Positive for pneumococcus	Negative for pneumococcus
Healthy	2145	389
Pneumonia	147	11
Severe pneumonia	11	5
Very severe pneumonia	9	1

Supplementary Table 4 Genomic data associated with the nasopharyngeal samples taken from individuals in the studied cohort. Many samples had been characterised previously through sequencing of individual isolates, or deep sequencing. Alternatively, data from other pneumococci in the same household, or from other hosts in this "immunology cohort", or from the wider Maela study, could be matched to samples not directly characterised by sequencing, using information on both their inferred serotype and GPSC.

Genomic Data	Frequency
No pneumococcal carriage	406
Single isolate data	357
Deep sequencing data	495
Single isolate and deep sequencing data	241
Matched serotype or GPSC within mother-child pair	1069
Matched serotype or GPSC within immunology cohort	18
Matched serotype or GPSC within Maela cohort	16
No genome data	116

Supplementary Table 5 Linear mixed-effects models used to analyse variation in IgG binding levels to ABTs, accounting for maternal antibody levels and history of exposure to proteins during carriage. The same notation is used as in Supplementary Table 1. The models feature the additional terms ABT (as these models were not fitted to non-ABTs); Type_{ABT} (referring to the functional classification shown in Fig. 2), and $\text{Maternal}_{\text{Individual,ABT}}$ (referring to the IgG binding to the ABT in the maternal birth sample from the corresponding individual). Models 1 and 2 were fitted to ABTs to which all individuals were exposed by 12 mo. Models 3, 4 and 5 were fitted to the complementary set of ABTs, to which the cohort was partially exposed at 12 mo. All models were fitted to data collected at sampling ages of 6, 12, 18 and 24 mo. The AIC values are compared in Supplementary Fig. 49. Using the AIC, model 2 was selected over model 1 for ABTs to which the cohort was universally exposed by 12 mo, and model 3 was selected to represent the ABTs to which the cohort was partially exposed by 12 mo.

Index	Model structure	AIC	BIC
1	$\text{IgG}_{\text{ABT}} \sim \text{ABT} + \text{Age}_{\text{Individual}} * \text{Type}_{\text{ABT}} + (1\|\text{Individual})$	58936	59749
2	$\text{IgG}_{\text{ABT}} \sim \text{ABT} + \text{Age}_{\text{Individual}} * \text{Type}_{\text{ABT}} + \text{Maternal}_{\text{Individual,ABT}} * \text{ABT} + (1\|\text{Individual})$	58878	60368
3	$\text{IgG}_{\text{ABT}} \sim \text{ABT} + \text{Age}_{\text{Individual}} * \text{Type}_{\text{ABT}} + \text{Exposure}_{\text{SinceBirth}} * \text{ABT} + \text{Maternal}_{\text{Individual,ABT}} * \text{ABT} + (1\|\text{Individual})$	45244	47213
4	$\text{IgG}_{\text{ABT}} \sim \text{ABT} + \text{Age}_{\text{Individual}} * \text{Type}_{\text{ABT}} + \text{Exposure}_{\text{FirstSixMonths}} * \text{ABT} + \text{Maternal}_{\text{Individual,ABT}} * \text{ABT} + (1\|\text{Individual})$	46206	48151
5	$\text{IgG}_{\text{ABT}} \sim \text{ABT} + \text{Age}_{\text{Individual}} * \text{Type}_{\text{ABT}} + \text{Exposure}_{\text{SinceLastSample}} * \text{ABT} + \text{Maternal}_{\text{Individual,ABT}} * \text{ABT} + (1\|\text{Individual})$	45874	47843

Supplementary Table 6 Linear mixed-effects models used to analyse how variation in IgG binding levels to ABTs related to the diagnosis of pneumonia over the preceding six months, accounting for maternal antibody levels and history of exposure to proteins during carriage. The same notation is used as in Supplementary Table 5. The models feature the additional term $\text{Diagnosis}_{\text{Pneumonia}}$, which indicated whether an individual was associated with at least one clinical pneumonia diagnosis in the preceding six months. Models 1, 2 and 3 were fitted to ABTs to which all individuals were exposed by 12 mo. Models 4, 5 and 6 were fitted to the complementary set of ABTs, to which the cohort was partially exposed at 12 mo. All models were fitted to data collected at sampling ages of 6, 12, 18 and 24 mo. The AIC values are compared in Supplementary Fig. 66. The AIC recommended model 2 for ABTs to which there was universal exposure across the cohort at 12 mo, and model 6 for ABTs to which there was partial exposure across the cohort at 12 mo. However, this would result in difficulties when comparing the relationship between pneumonia episodes and antibody levels between the two subsets of ABTs. This was because the association of IgG levels with pneumonia episodes is type-independent in model 2, but type-dependent in model 6. Therefore, models 2 and 5 were selected using the BIC, as this generated comparable analyses across all ABTs.

Index	Model structure	AIC	BIC
1	$\text{IgG}_{\text{ABT}} \sim \text{ABT} + \text{Age}_{\text{Individual}} * \text{Type}_{\text{ABT}} + \text{Maternal}_{\text{Individual,ABT}} * \text{ABT} + (1\|\text{Individual})$	58878	60368
2	$\text{IgG}_{\text{ABT}} \sim \text{ABT} + \text{Age}_{\text{Individual}} * \text{Type}_{\text{ABT}} + \text{Maternal}_{\text{Individual,ABT}} * \text{ABT} + \text{Age}_{\text{Individual}} * \text{Diagnosis}_{\text{Pneumonia}} + (1\|\text{Individual})$	58682	60204
3	$\text{IgG}_{\text{ABT}} \sim \text{ABT} + \text{Age}_{\text{Individual}} * \text{Type}_{\text{ABT}} + \text{Maternal}_{\text{Individual,ABT}} * \text{ABT} + \text{Age}_{\text{Individual}} * \text{Diagnosis}_{\text{Pneumonia}} * \text{Type} + (1\|\text{Individual})$	58688	60337
4	$\text{IgG}_{\text{ABT}} \sim \text{ABT} + \text{Age}_{\text{Individual}} * \text{Type}_{\text{ABT}} + \text{Exposure}_{\text{SinceLastSample}} * \text{ABT} + \text{Maternal}_{\text{Individual,ABT}} * \text{ABT} + (1\|\text{Individual})$	45244	47213
5	$\text{IgG}_{\text{ABT}} \sim \text{ABT} + \text{Age}_{\text{Individual}} * \text{Type}_{\text{ABT}} + \text{Exposure}_{\text{SinceLastSample}} * \text{ABT} + \text{Maternal}_{\text{Individual,ABT}} * \text{ABT} + \text{Age}_{\text{Individual}} * \text{Diagnosis}_{\text{Pneumonia}} + (1\|\text{Individual})$	44983	46983
6	$\text{IgG}_{\text{ABT}} \sim \text{ABT} + \text{Age}_{\text{Individual}} * \text{Type}_{\text{ABT}} + \text{Exposure}_{\text{SinceLastSample}} * \text{ABT} + \text{Maternal}_{\text{Individual,ABT}} * \text{ABT} + \text{Age}_{\text{Individual}} * \text{Diagnosis}_{\text{Pneumonia}} * \text{Type} + (1\|\text{Individual})$	44966	47186

Supplementary Table 7 Linear mixed-effects models used to analyse the relationship between IgG binding levels to ABTs and both clinical pneumonia episodes and frequency of colonisation. Two types of model structure were used: one applied to ABTs to which the cohort was universally exposed at 12 mo (derived from model 2 in Supplementary Table 6), and the other applied to ABTs to which the cohort was partially exposed at 12 mo (derived from model 5 in Supplementary Table 6). Each model structure was fitted to four different diagnoses: high-frequency colonisation ($Diagnosis_{FrequentColonisation}$), pneumonia ($Diagnosis_{Pneumonia}$), severe pneumonia ($Diagnosis_{SeverePneumonia}$) and very severe pneumonia ($Diagnosis_{VerySeverePneumonia}$). Models were separately fitted to estimate IgG levels after an individual was diagnosed with the condition since the preceding serum sample (using data from 6, 12, 18 and 24 mo samples), or to estimate the IgG levels before an individual was diagnosed prior to the next serum sample (using umbilical cord and 6, 12 and 18 mo samples). Hence sixteen models were fitted in total. The notation is otherwise the same as in Supplementary Table 6.

Diagnosis	Model Type	Model Structure
Frequent colonisation	Universal exposure	$\mathrm{IgG_{probe}} \sim \mathrm{ABT} + \mathrm{Age_{Individual}} * \mathrm{Type_{ABT}} + \mathrm{Maternal_{Individual,ABT}} * \mathrm{ABT} + \mathrm{Age_{Individual}} * \mathrm{Diagnosis_{FrequentColonisation}} + (1\|\mathrm{Individual})$
Frequent colonisation	Partial exposure	$\mathrm{IgG_{probe}} \sim \mathrm{ABT} + \mathrm{Age_{Individual}} * \mathrm{Type_{ABT}} + \mathrm{Exposure_{SinceLastSample}} * \mathrm{ABT} + \mathrm{Maternal_{Individual,ABT}} * \mathrm{ABT} + \mathrm{Age_{Individual}} * \mathrm{Diagnosis_{FrequentColonisation}} + (1\|\mathrm{Individual})$
Pneumonia	Universal exposure	$\mathrm{IgG_{probe}} \sim \mathrm{ABT} + \mathrm{Age_{Individual}} * \mathrm{Type_{ABT}} + \mathrm{Maternal_{Individual,ABT}} * \mathrm{ABT} + \mathrm{Age_{Individual}} * \mathrm{Diagnosis_{Pneumonia}} + (1\|\mathrm{Individual})$
Pneumonia	Partial exposure	$\mathrm{IgG_{probe}} \sim \mathrm{ABT} + \mathrm{Age_{Individual}} * \mathrm{Type_{ABT}} + \mathrm{Exposure_{SinceLastSample}} * \mathrm{ABT} + \mathrm{Maternal_{Individual,ABT}} * \mathrm{ABT} + \mathrm{Age_{Individual}} * \mathrm{Diagnosis_{Pneumonia}} + (1\|\mathrm{Individual})$
Severe pneumonia	Universal exposure	$\mathrm{IgG_{probe}} \sim \mathrm{ABT} + \mathrm{Age_{Individual}} * \mathrm{Type_{ABT}} + \mathrm{Maternal_{Individual,ABT}} * \mathrm{ABT} + \mathrm{Age_{Individual}} * \mathrm{Diagnosis_{SeverePneumonia}} + (1\|\mathrm{Individual})$
Severe pneumonia	Partial exposure	$\mathrm{IgG_{probe}} \sim \mathrm{ABT} + \mathrm{Age_{Individual}} * \mathrm{Type_{ABT}} + \mathrm{Exposure_{SinceLastSample}} * \mathrm{ABT} + \mathrm{Maternal_{Individual,ABT}} * \mathrm{ABT} + \mathrm{Age_{Individual}} * \mathrm{Diagnosis_{SeverePneumonia}} + (1\|\mathrm{Individual})$
Very severe pneumonia	Universal exposure	$\mathrm{IgG_{probe}} \sim \mathrm{ABT} + \mathrm{Age_{Individual}} * \mathrm{Type_{ABT}} + \mathrm{Maternal_{Individual,ABT}} * \mathrm{ABT} + \mathrm{Age_{Individual}} * \mathrm{Diagnosis_{VerySeverePneumonia}} + (1\|\mathrm{Individual})$
Very severe pneumonia	Partial exposure	$\mathrm{IgG_{probe}} \sim \mathrm{ABT} + \mathrm{Age_{Individual}} * \mathrm{Type_{ABT}} + \mathrm{Exposure_{SinceLastSample}} * \mathrm{ABT} + \mathrm{Maternal_{Individual,ABT}} * \mathrm{ABT} + \mathrm{Age_{Individual}} * \mathrm{Diagnosis_{VerySeverePneumonia}} + (1\|\mathrm{Individual})$
