# Supplementary material for: Genomic and panproteomic analysis of the development of infant immune responses to antigenically-diverse pneumococci
Source: Nat Commun. 2024 Jan 8;15:355. doi: 10.1038/s41467-023-44584-2 (PMC10774285; doi:10.1038/s41467-023-44584-2)
Supplement: Supplementary file 3 — Description of Additional Supplementary Files [file 41467_2023_44584_MOESM3_ESM.pdf]

Description of Additional Supplementary Files

Supplementary Data 1 : Epidemiological data associated with serological samples. Birth weights were measured in grammes.

Supplementary Data 2 : Accession codes for genomic datasets associated with each immunological dataset.

Supplementary Data 3 : Timing and severity of episodes of clinical pneumonia in the cohort
